# Melatonin Repairs the Lipidome of Human Hepatocytes Exposed to Cd and Free Fatty Acid‐Induced Lipotoxicity

**DOI:** 10.1111/jpi.70047

**Published:** 2025-04-07

**Authors:** Anna Migni, Desirée Bartolini, Giada Marcantonini, Roccaldo Sardella, Mario Rende, Alessia Tognoloni, Maria Rachele Ceccarini, Francesco Galli

**Affiliations:** ^1^ Department of Pharmaceutical Sciences University of Perugia Perugia Italy; ^2^ Department of Medicine and Surgery University of Perugia Perugia Italy

**Keywords:** cadmium, free fatty acids, hepatocytes, lipidomics, lipotoxicity, melatonin, NAFLD

## Abstract

Hepatocyte lipotoxicity is central to the aetiology of nonalcoholic fatty liver disease (NAFLD), a leading cause of liver failure and transplantation worldwide. Long‐lasting toxic pollutants have increasingly been considered as environmental risk factors of NAFLD. These include cadmium (Cd), a metal that synergizes with other cellular toxicants and metabolic stimuli to induce fat build‐up and lipotoxicity. Recent studies demonstrated that melatonin (MLT) holds great potential as repairing agent in this form of hepatocyte lipotoxicity. In this study, the molecular hints of this MLT effect were investigated by lipidomics analysis in undifferentiated HepaRG cells, a human pre‐hepatocyte cell line, exposed to Cd toxicity either alone or combined with prototypical free fatty acids (FFA), namely the saturated species palmitic acid and the monounsaturated oleic acid (OA and PA, respectively), to simulate the cellular lipotoxicity conditions of fatty liver disease. Cd exposure synergized with FFAs to induce cellular steatosis, and PA produced higher levels of lipotoxicity compared to OA by leading to increased levels of H_2_O_2_ production and apoptotic death. These effects were associated with changes of the cellular lipidome, which approximate those of NAFLD liver, with differentially expressed lipids in different classes that included triacylglycerols (TG), di‐ and mono‐acylglycerols, phospholipids (PL), sphingolipids, acylcarnitines and FA; characteristic differences were observed in all these classes comparing the combinations of Cd exposure with PA or OA treatments. MLT significantly reduced the effects of either individual or combinatorial treatments of Cd and FFAs on lipotoxicity hallmarks, also repairing most of the alterations of the cellular lipidome, including those of the chain length and number of double bonds of acyl residues esterified to TG and PL classes. These findings and their bioinformatics interpretation suggest a role for the earliest acyl elongase and desaturase steps of FA metabolism in this repairing effect of MLT; biochemistry studies validated such interpretation identifying a specific role for SCD1 activity. This lipidomics study shed light on the cytoprotective mechanism of MLT in Cd and FFA‐induced hepatocyte lipotoxicity, highlighting a repairing effect of this molecule on the cellular lipidome, which may hold therapeutic potential in fatty liver diseases.

## 1 Introduction

Melatonin (MLT) is a cytoprotective agent efficient in preventing the effects of a range of toxic stimuli [1]. These include the exposure to potentially toxic elements (PTE) as cadmium (Cd) [[2, 3] and references therein], an increasingly concerning threat since of its environmental diffusion and proposed role in chronic ailments such as cancer and some endocrine and metabolic diseases [4, 5, 6]. Alike other PTE [7, 8], Cd toxicity derives from its ability to increase the production of reactive oxygen species (ROS) by inducing damages to mitochondria and other cellular organelles involved in molecular oxygen activation; these ROS‐inducing effects of Cd can promote oxidative stress and metabolic dysfunction in different tissues and cell types [2, 3, 9, 10, 11], including the liver [5, 6, 12, 13, 14, 15]. Recent studies propose a specific mechanism of hepatocyte damage by this PTE, consisting in the induction of cellular fat build‐up and lipotoxicity [12, 16]; these are key pathogenic events of nonalcoholic fatty liver disease and steatohepatitis (NAFLD and NASH, respectively). These liver diseases are often encountered in metabolic disease and obese patients [17, 18] and may lead to ectopic fat accumulation and lipotoxicity also in other tissues, such as pancreatic, muscle and arterial. This contributes to the development of a condition of multimorbidity encompassing insulin resistance, atherosclerotic cardiovascular disease and an increased risk of cancers, particularly of the gastrointestinal tract, these include hepatocellular carcinoma as a direct consequence of the carcinogenetic effects of hepatocellular lipotoxicity and oxidative stress [19].

Together these aspects suggest a role of Cd exposure in the pathogenesis of liver diseases aside of, and in combination with, other causes of steatosis and lipotoxicity of the hepatocyte; these include an increased flux of dietary and/or endogenous free fatty acids (FFA), which represents a para‐physiological event that may transform to pathologic by evolving to chronic state, as observed for example in overnourished subjects as well as in visceral obesity and metabolic patients [12, 17].

Recent studies by some of us demonstrated the potential of MLT as repairing or cytoprotective agent in human hepatocytes exposed to Cd and FFA‐induced lipotoxicity [20]. In the present study, a high‐resolution LC‐Q/TOF lipidomics procedure was utilized to characterize this MLT function at the molecular and mechanistic level. This lipidomics study may help to shed more light on the therapeutic potential of this molecule in hepatocyte lipotoxicity and fatty liver disease.

## 2 Materials and Methods

### 2.1 Cell Model and Treatments

HepaRG cells (Thermo Scientific, MA, USA) were cultured in William's E medium (Sigma‐Aldrich, St. Louis, MI, USA) containing 1% GlutaMAX (Invitrogen, Carlsbad, CA, USA), 10% fetal bovine serum (FBS, GIBCO, Life Technologies, Carlsbad, CA, USA), 5 μg/mL of human insulin (Sigma‐Aldrich), and 50 μM hydrocortisone 21‐hemisuccinate (Sigma‐Aldrich, St. Louis, MI, USA). Experiments were carried out between cell passage 10 and 15. The cells were maintained in culture at 37°C with a humidified atmosphere of 5% CO_2_. Cell treatments were as shown in Figure 1A and consisted of a 24‐h pretreatment step with 50 μM cadmium chloride (CdCl_2_; 202908, Sigma‐Aldrich, St. Louis, MI, USA) followed by a 48‐h treatment with FFA in the presence or absence of 50 nM MLT (N‐Acetyl‐5‐methoxytryptamine, M5250, Sigma‐Aldrich) to study its cytoprotective or repairing activity during hepatocyte lipotoxicity according to preclinical evidence obtained before and reported in [20]. Oleic acid (OA, O1008, Sigma‐Aldrich, St. Louis, MI, USA) dissolved in DMSO and palmitic acid (PA, P5585, BioXtra, ≥ 99%), prepared as reported in [20, 21], were the FFA species used individually or in combination (each at 200 µM final concentration) to treat HepaRG cells after Cd pretreatment. These two FFA species produce different levels of steatosis and lipotoxicity in HepaRG cells and other cell lines, with saturated species PA that presents lower fat build up but higher cytotoxicity effects than the monounsaturated OA (described in [20, 21] and references therein).

Figure 1Cytoprotective effect of MLT in HepaRG human liver cells exposed to CdCl_2_ and FFA‐induced lipotoxicity. (A) Experimental protocol and treatments. Cell treatments consisted of a 24‐h pretreatment step with 50 μM cadmium chloride followed by a 48‐h treatment with the FFA species OA and PA (200 µM final concentration each), in the presence or absence of 50 nM MLT. (B) Light microscopy images of Oil Red O (ORO) and hematoxylin stained cells to identify lipid droplets and cell nucleus, respectively (40X magnification). (C) Cellular lipids were measured by FACS‐scan using the fluorescent probe Nile‐Red‐FITC. (D) H_2_O_2_ levels were assessed in the cell medium as a measure of cellular ROS production during lipotoxicity. (E) Early and late apoptosis levels were studied by FACS‐scan using the fluorescent probes Annexin‐V and PI. One‐way ANOVA test: *p < 0.05; **p < 0.01; ***p < 0.0001 (control test without treatments vs. all treatments); ^#^ *p* < 0.05; ^##^ *p* < 0.01 (CdCl_2_ vs. CdCl_2_ + FFAs); ^*p* < 0.01 (Cd+MLT vs Cd); §*p* < 0.05 (CdCl_2_ +FFAs vs CdCl_2_ + FFAs+ MLT).

**Figure:**
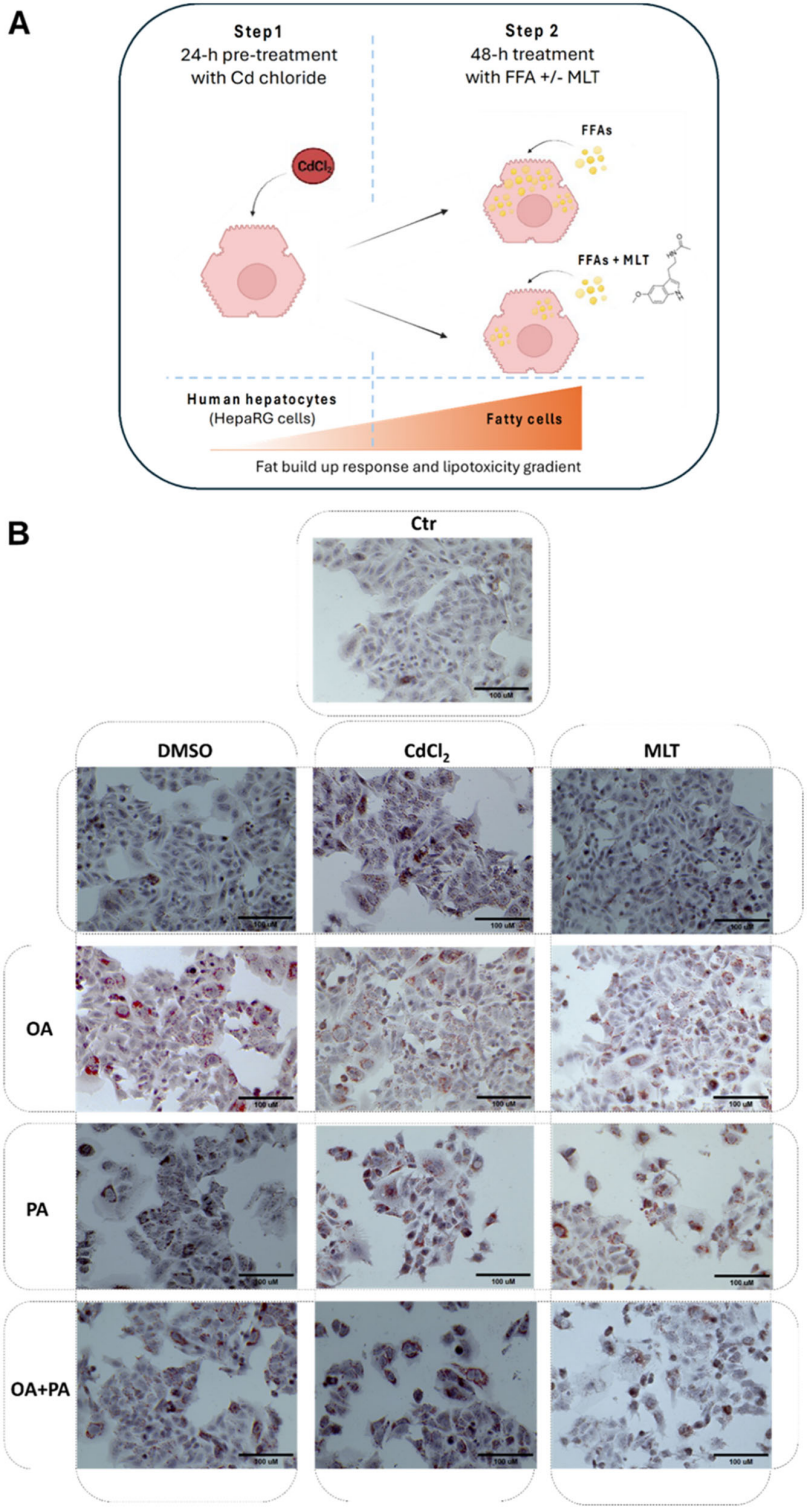

**Figure:**
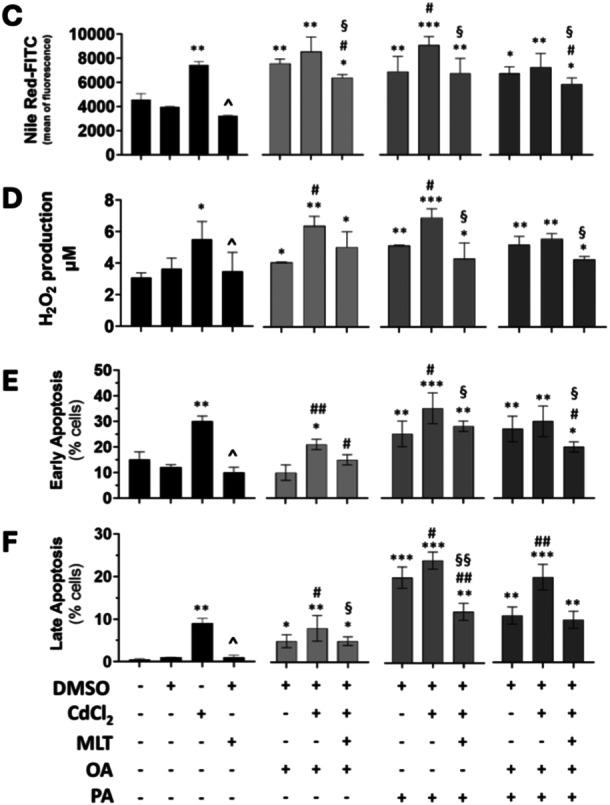

### 2.2 Lipid Droplets Analysis

Cellular lipids were measured using Oil Red O (ORO) staining according to the procedure described in [21]. In brief, after treatments, HepaRG cells were fixed with 10% neutral buffered formalin (Leica) for 30 min and then were washed twice with sterile bi‐distilled water and incubated with 60% isopropanol (Sigma‐Aldrich, St. Louis, MI, USA) for 5 min. The cells were stained with ORO solution for 2 min, followed by four washes with sterile bi‐distilled water. Next, the cells were stained with hematoxylin solution (Sigma‐Aldrich, St. Louis, MI, USA) for 1 min. After another wash with sterile bi‐distilled water, the red lipid droplets were observed with a light microscope. using an EVOSTM XL Core imaging system (Thermo Fisher) for qualitative analysis. For quantitative neutral lipid droplets analysis were used Cell‐Based Assay Nile Red Solution (Cayman Chemicals) by FACS (BD Accuri C6 Plus System, BD Biosciences, San Jose, CA, USA).

### 2.3 H_2_O_2_ Production

Extracellular H_2_O_2_ levels were measured using a colorimetric assay kit (Elabscience, Houston, TX, USA) following the manufacturer's procedure. The absorbance was measured at 405 nm using a DTX880 Multimode Detector microplate reader (Beckman Coulter).

### 2.4 Apoptosis Assay

Annexin V‐FITC/PI Apoptosis kit (E‐CK‐A21, Elabscience, Houston, TX, USA) at cytofluorometer was used to assess early and late apoptosis levels as reported in [20].

### 2.5 Lipid Extraction

The cell pellet (1 × 10^6^ cells) was resuspended in 1 mL of ultrapure water and then transferred to a 15 mL glass tube and spiked with 15 µL of the internal standards mix for lipidomics analysis (330707‐1EA, SPLASH® LIPIDOMIX MASS SPEC STANDARD, AVANTI). Total lipids were extracted from all samples following the Bligh and Dyer method [22] with minor modifications. Briefly, 3.75 mL of dichloromethane/methanol 1:2 (v:v) were added to samples, and after vortexing these were incubated for 30 min in ice. Then 1.25 mL of dichloromethane and 1.25 mL of ultrapure water were pipetted in the tubes and the samples were vortexed for 1 min and centrifuged at 1,000 rpm and 20°C for 5 min to obtain two phases. Finally, the organic bottom phase was collected in a 20 mL glass tube and dried down under a gentle nitrogen stream. The extraction procedure was repeated twice to increase lipid fraction recovery. The extracted samples were resuspended in 150 µL of MS‐grade methanol (Sigma‐Aldrich, USA) for the analysis.

### 2.6 LC‐MS Analysis of the Cellular Lipidome

#### 2.6.1 LC Conditions

For untargeted lipidomic analysis, the liquid chromatography was carried out with an Agilent 1290 Infinity LC system (Agilent Technologies Inc. Waldbronn, Germany) equipped with a Zorbax Eclipse Plus C18 column (1.8 μm, 2.1 × 50 mm, 95 Å; Agilent, USA).

The column and the autosampler temperature were kept at 50°C and 4°C, respectively, during the analysis, and the injection volume was 5 µL. Lipid separation was achieved by using a binary gradient of water/methanol (9:1; v:v) as elution phase A and acetonitrile/methanol/isopropanol (2:3:5; v:v:v) as phase B; ammonium acetate (Sigma‐Aldrich, USA) was added to both the phases at 10 mM final concentration. The flow rate was maintained at 450 μL/min and the gradient was 0–1 min isocratic 50% A, 1–4 min linear increase up to 24% A, 4–14 min linear from 24% to 14% A, 15 min linear from 24% to 3% A, and 15–18 min isocratic 97% B followed by an equilibrating step at 50% A for 5 min.

#### 2.6.2 MS Conditions

Each sample was analysed in triplicate and data acquisition was obtained in full scan mode in both positive and negative ionization. Fragmentation experiments were carried out on pooled quality control (QC) samples (equimolar mix of all samples) using iterative (4 cycles) automated (auto)‐MS/MS acquisition mode in both the ionization modes.

MS detection was carried out with an Agilent 6560 IM‐QTOF mass spectrometer equipped with Agilent Dual Jet Stream Source; ionization in positive and negative mode was performed using the following parameters: V_cap_ 3000 V, fragmentor 300, gas temperature 200°C, drying gas flow 10 L/min, nebulizer gas pressure 50 psi, sheath gas temperature 300°C, and sheath gas flow 12 L/min. Mass range for acquisition mode MS1 was 100–1700 m/z, whereas for auto MS2 was 40–1700 m/z with a scan rate of 3 spectra/sec for both methods. Collision energy for MS2 experiments was 20 eV.

Data quality was ensured according to [23] by sample randomization for lipid extraction and data acquisition, three injections of blank followed by three injections of the internal standard mix to equilibrate the LC system before run the samples, injections of QC sample every five samples to check for signal deviation for time, mass accuracy checking, signal abundance and retention time shift of internal standards in every samples.

### 2.7 MS Data Processing and Statistical Analysis

Lipid identification and annotation was carried out by Lipid Annotator Software (Agilent, USA) by matching the mass spectra obtained from MS2 acquisition of QC sample against the *in‐silico* LipidBlast database. For this purpose, all features with a mass deviation ≥ 10 ppm, fragment score ≤ 30 and total score ≤ 60 compared to the reference database, were discarded. Matched results were exported to MassHunter PCDL Manager (Agilent, USA) and MS1 raw data files were processed using MassHunter Profinder 10.0 Software (Agilent, USA). After peak alignment to a reference file of internal standard mix, peak extraction was performed by matching the PCDL databases to the raw files of samples. In this regard, match tolerance for mass and retention time was set at +/−10.00 ppm and 0.3 min, respectively. Contribution to overall score was determined by mass (100%), retention time (100%), isotope abundance (60%) and spacing (50%) scores. Compounds with the overall score < 75% were discarded. Extracted ion chromatograms (EIC) peaks with an absolute height ≥ 1000 counts were smoothed (Gaussian function) and integrated (Agile2). Quality control of extracted features was manually performed by checking peak shape and integration, and mass spectra following the annotation rules of LIPID MAPS consortium (https://lipidmaps.org/).

Normalization and filtering were performed using Mass Profiler Professional (MPP) Software (Agilent). Lipid species, normalized for the deuterated standards, were filtered according to the minimum absolute abundance (5000 counts) and to sample frequency (only lipids present in 75% of samples were maintained).

Log2 normalized intensities for each compound were corrected for the CTL data to directly compare all the different treatments each other; these were plotted using GraphPad Prism 9.0.2 to build a heatmap.

Principal component analysis (PCA) and partial least squares‐discriminant analysis (PLS‐DA) were carried out using MetaboAnalyst 6.0 (https://www.metaboanalyst.ca/). Lipid species with a variable importance in projection index (VIP) greater than one were used to highlight the overall effect of MLT on the cellular lipidome.

False discovery rate (FDR)‐corrected moderated *t*‐test (adjusted *p* value < 0.01, FC cutoff = 1.5) was used to perform lipid expression pairwise comparisons between different cell treatments; significantly modulated lipids were visualized using GraphPad Prism 9.0.2. by volcano plots and as sum composition by plotting the number of double bonds and carbons in dots charts for each lipid class.

### 2.8 Pathway Analysis

After KEGG ID conversion, significantly modulated lipids by MLT were subjected to pathway and metabolic set enrichment analysis based on global test algorithm using MetaboAnalyst 6.0 (https://www.metaboanalyst.ca/).

Pathway impact and enrichment ratio were computed by hits (observed metabolite) and expected metabolites annotated on specific KEGG pathways and the Relational Database of Metabolic Pathways (RaMP‐DB), respectively.

Enrichment method and topology measure were performed using hypergenomic test and relative‐betweenness centrality, respectively.

### 2.9 Post‐Hoc Verification of Lipidomics Results: Stearoyl‐CoA Desaturase (SCD1) Investigation

The role of SCD1 (or Δ9‐desaturase) in the repairing and cytoprotective effects of MLT was investigated inducing the transient inhibition of *SCD1* gene expression by SCD1 Human siRNA Oligo Duplex (Locus ID 6319; OriGene Technologies Inc. Rockville, MD, USA). Briefly, after Cd pretreatment the cells were transfected with the oligo duplex for 5 h at 50 nM final concentration. SCD1 protein expression was assessed to verify the silencing procedure by immunoblot; briefly, 10 μg of total cellular proteins denatured in loading buffer containing dithiothreitol (DTT) were separated by gradient (4–12%) sodium dodecyl sulfate‐polyacrylamide gel electrophoresis (SDS‐PAGE) and then transferred to a nitrocellulose membrane for the sequential incubation with blocking buffer for 1 h (TBS containing 5% dry milk and 0.1% Tween‐20) and then overnight with an anti‐SDC1 primary antibody diluted 1:1000 vol/vol (#2438, Cell Signalling Technology, Inc). After repeated washes in TBS containing 0.1% Tween‐20, the nitrocellulose sheet was incubated for 1 h at room temperature with a horseradish peroxidase‐conjugated anti‐rabbit secondary antibody (#7074, Cell Signalling Technology, Inc) diluted 1:2000 vol/vol in blocking buffer, and protein detection was performed using an enhanced luminol‐based chemiluminescent (ECL) detection system (ECL Clarity, Bio‐Rad). The effects of SCD1 silencing on cellular lipids and H_2_O_2_ levels were assessed as described earlier in section 2.2 and 2.3, and cell viability was assessed by MTT test as reported in [20].

The effect of SCD1 enzymatic activity on the fat build up response to lipotoxicity treatments and MLT was also investigated using the pharmacological inhibitor A‐939572 (Cayman Chemical) at 6 nM final concentration in the treatment medium.

## 3 Results

### 3.1 MLT Reduces the Levels of Cellular Steatosis and Lipotoxicity Effects in Heparg Cells Exposed to Cd and FFA

Cd and FFA were studied for their fat build‐up and lipotoxicity effects in HepG2 human pre‐hepatocytes as separate or combinatorial treatments. The experimental protocol adopted in these studies (Figure 1A) was designed to simulate the pathophysiology of Cd exposure in human hepatocytes and its association with cellular lipotoxicity as a specific aetiology factor in fatty liver diseases. In this context, FFA were combined with Cd since an increased flux of cellular lipids, and particularly of FFAs of endogenous and/or dietary origin, is the main cause of hepatocyte lipotoxicity as disease mechanism in human NAFLD [17]. In this protocol we studied both the monounsaturated species OA and the saturated species PA which are known to induce different levels and types of cellular steatosis, thus resulting in different levels of lipotoxicity and loss of liver cellular mass and function [20, 21, 24, 25].

Cd and FFA studied as separate treatments induced similar levels of cellular lipids in HepaRG cells (Figure 1B and 1C), whereas the levels of lipotoxicity indicators (Figure 1D‐F), and particularly of late apoptotic cell death (Figure 1F), followed the peeking order PA > OA + PA ≅ Cd ≥ OA, thus confirming a more benign phenotype of OA compared to PA and Cd treatments also in this cell model [20].

In combinatorial treatments (Figure 1A), FFA synergized with Cd to increase both the fat build‐up response of HepaRG cells (Figure 1B and 1C), and the levels of lipotoxicity hallmarks H_2_O_2_ and apoptosis (Figure 1D and 1E‐F, respectively). PA treatment produced higher cell death levels than OA treatment when combined with Cd exposure, and apparently OA mitigated this lipotoxicity effect of PA in combined treatments (e.g. OA + PA treatment combined with Cd pretreatment; Figure 1D and 1E‐F, respectively).

Melatonin significantly reduced the levels of cellular steatosis and lipotoxicity indicators (i.e. extracellular H_2_O_2_ and apoptosis) in HepaRG cells exposed to Cd either alone or combined with FFA treatments (Figure 1B‐F). Since MLT was administered to the cells after Cd exposure and together with FFA treatment (Figure 1A), these findings confirm that the cytoprotective properties of MLT in this in vitro model of hepatocyte lipotoxicity [20] is based on a repairing effect of this molecule on the cellular alterations induced by the lipotoxicity process.

### 3.2 MLT Repairs the Cellular Lipidome of Heparg Cells Exposed to Cd and FFA Induced Lipotoxicity

The untargeted lipidomics method used in this study provided a compound database (PCDL) of 654 annotated lipids belonging to different lipid classes identified in quality control (QC) samples, with 448 compounds identified in positive and 206 in negative acquisition mode (total ion counts chromatograms of QC and lipid species identified with these two ionization modes are shown in Supporting Information Figure S1 and S2, respectively). After peak extraction, normalization and filtering, 293 annotated features were reported in 75% of samples, and a complete list of identified lipids ranked by lipid class, relative averaged normalised Log2 intensities, mass, retention time and ion species used for identification, is shown in Supporting Information Table S1.

#### 3.2.1 Lipid Fingerprints and Multivariate Analysis of Lipid Species Modulated During Lipotoxicity and MLT Treatment

##### 3.2.1.1 Heatmap Chart Representation

**Figure 2:**
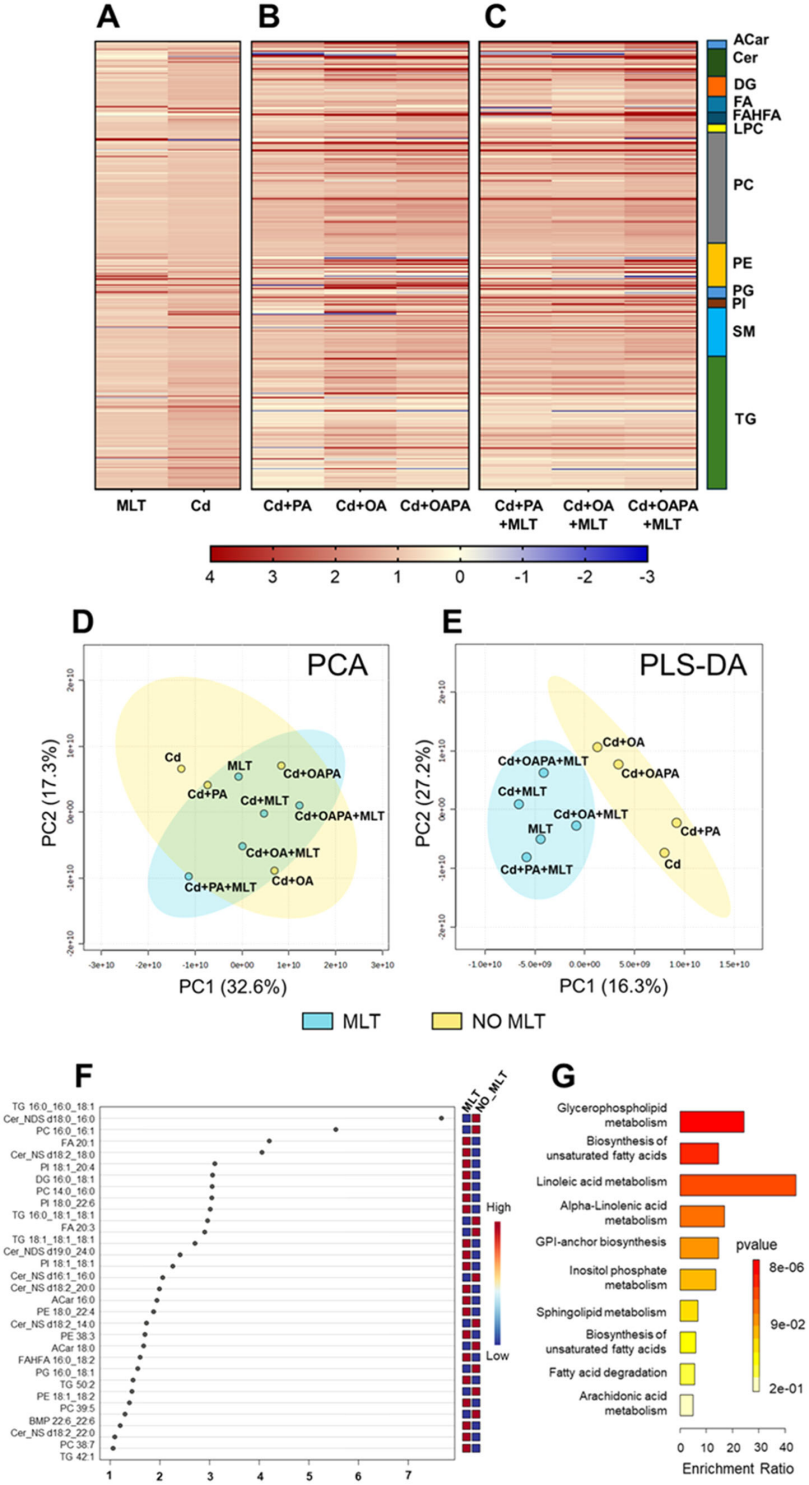
Effect of MLT on the lipidome of HepaRG cells exposed to Cd and FFA‐induced lipotoxicity. (A‐C) heatmap charts of the expression of lipid metabolites ranked by lipid classes (ordinate); data are shown as chromatographic peak intensities corrected for control test data (cells exposed to the treatment vehicle DMSO only) for direct comparison of the different treatments investigated in this study (abscissa). Cd and FFA were used alone or in different combinations to induce lipotoxicity and MLT was utilized as cytoprotective agent administered in combination with FFA after Cd exposure (Figure 1A). Different colours, ranging from blue (lower value) to red (higher value), indicate the level of lipid abundance expressed as Log2 median values of experiments and LC‐Q/TOF analyses that were both run in triplicate. (D, E) Differences between matrices of lipidomics data presented in these heatmaps were assessed by unsupervised and supervised multivariate analysis using PCA and PLS‐DA methods, respectively. Green dots correspond to treatments without MLT, whereas red dots to MLT treatments. (F) Lipid species with VIP score> 1 identified by PLS‐DA and included in PC1. The coloured boxes on the right indicate the relative level of the corresponding metabolite in cellular treatments with or without MLT (blue = lower; red = higher). (G) Pathways enrichment analysis. Lipid pathways with significant enrichment associated to lipid species (VIP > 1) that are modulated by MLT cytoprotective function in hepatocyte lipotoxicity. The p‐value is colour coded as shown in the legend and the enrichment ratio is reported in abscissa.

The median intensity of lipid metabolites identified in the different cell treatments are reported ranked by lipid class in the heatmap charts of Figure 2A‐C after normalization for the corresponding features in CTL samples (untreated cells). These heatmap images demonstrate that i) Cd exposure and its combination with FFA treatments produce distinct lipid fingerprints in HepaRG cells (Figure 2A, B), which identifies lipotoxicity as an heterogenous process with respect to cellular lipidome composition; ii) however, according to cellular steatosis data shown in Figure 1B and 1C, Cd + OA treatment was associated with a higher number of upregulated lipids compared to Cd + PA treatment (Figure 2B) mainly involving TG species, a characteristic lipid metabolism response that make OA a less lipotoxic FFA compared to PA [25]; iii) MLT treatment when considered alone (Figure 2A), produced only minor changes in the cellular lipidome, and iv) its cytoprotective effect was associated with changes in cellular lipids that partially reverted those of Cd (Figure 2A) and Cd + FFA treatments (Figure 2C vs 2B).

##### 3.2.1.2 Multivariate Analysis

**Figure 3:**
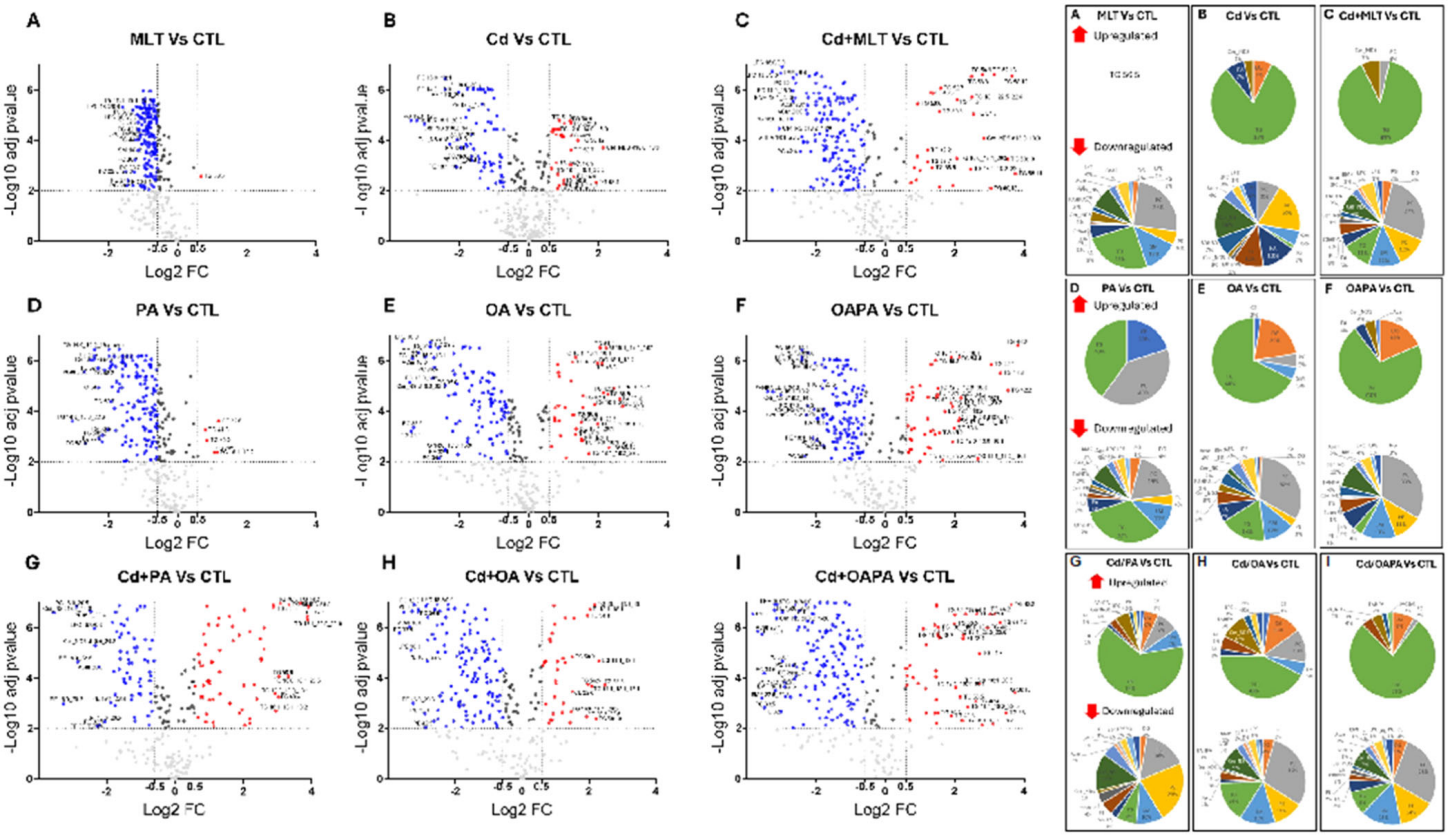
Differentially expressed lipid metabolites in HepaRG cells exposed to Cd toxicity and MLT treatment (A‐C), and in Cd and FFA‐induced lipotoxicity (D‐I). Volcano plot charts (scatter plot charts on the left) show differentially expressed lipids (adj *p*‐value < 0.01; FC cutoff = 1.5) identified in the various treatments by comparison with the corresponding lipid features of control cells (cells exposed to the treatment vehicle DMSO only). Relative changes in the corresponding lipid classes are shown in the pie charts on the right. Legend to Volcano plot symbols: Blue = significantly downregulated features (FC < ‐ 1.5). Red = significantly upregulated features (FC > 1.5). Dark grey = significantly modified species with ‐ 1.5 < FC < 1.5. Light grey = non‐significantly modified species.

PCA (Figure 2D) is a multivariate analysis method widely utilized for statistical evaluation and interpretation of omics data that was implemented in this study to further explore the changes of the cellular lipidome during lipotoxicity treatments. This data analysis model identified differences in the lipid components differentially modulated by the combinations of Cd pretreatment with PA or OA treatments, which mainly distribute throughout the PC1 axis; however, the model provided poor resolution between the variables associated with the MLT cytoprotective effect and lipotoxicity treatments (shown by the overlapping of the elliptic clusters in Figure 3D). When PLS‐DA was utilized to address class separation by MLT effect (Figure 2E), discriminating variables in each component of the multivariate model were identified and differential levels of lipid features that contribute to such discrimination are shown in Figure 2F with the top‐30 lipids extracted by VIP score (VIP > 1); these included:

i. ceramides (Cer) and TG that are the main classes of lipids that respond to MLT treatment (40% of VIP > 1 species). These lipid features are strongly associated with hepatocyte lipotoxicity ([17] and references therein);
ii. PC, phosphatidylinositols (PI) and acylcarnitines (Acar) that were upregulated, suggesting a role for MLT in enhancing glycerophospholipid metabolism and β‐oxidation processes of the hepatocyte to counteract their changes during the earliest steps of NAFLD [17];
iii. phosphatidylglycerols (PG) and their derived species bis(monoacylglycerol)phosphate (BMP), namely BMP 22:6/22:6, that are down and upmodulated, respectively. It is worth noting that the BMP class is specifically distributed at the level of lysosomes [26], a subcellular compartment with key role in preventing hepatocyte lipotoxicity by leading to LD formation and confinement of the neutral lipid excess [27].
iv. increased levels of fatty acid (FA) 20:1 (included in top10 VIP metabolites) and decreased levels of FA 20:3. The changes observed in these unsaturated species demonstrate that MLT affects FA metabolism facilitating PA and OA uptake and elongation.

These VIP score data were used to identify lipid metabolism pathways modulated by MLT (Figure 2G, KEGG‐based identification); these included: linoleic acid metabolism, glycerophospholipid metabolism, alpha‐linolenic acid metabolism, glycosylphosphatidylinositol (GPI)‐anchor biosynthesis, biosynthesis of unsaturated fatty acids, inositol phosphate metabolism, sphingolipid metabolism, fatty acid degradation and arachidonic acid metabolism.

#### 3.2.2 Pairwise Comparison (Univariate) Analysis of Lipid Metabolites

##### 3.2.2.1 Individual Treatments With Cd, FFAs and MLT

**Figure 4:**
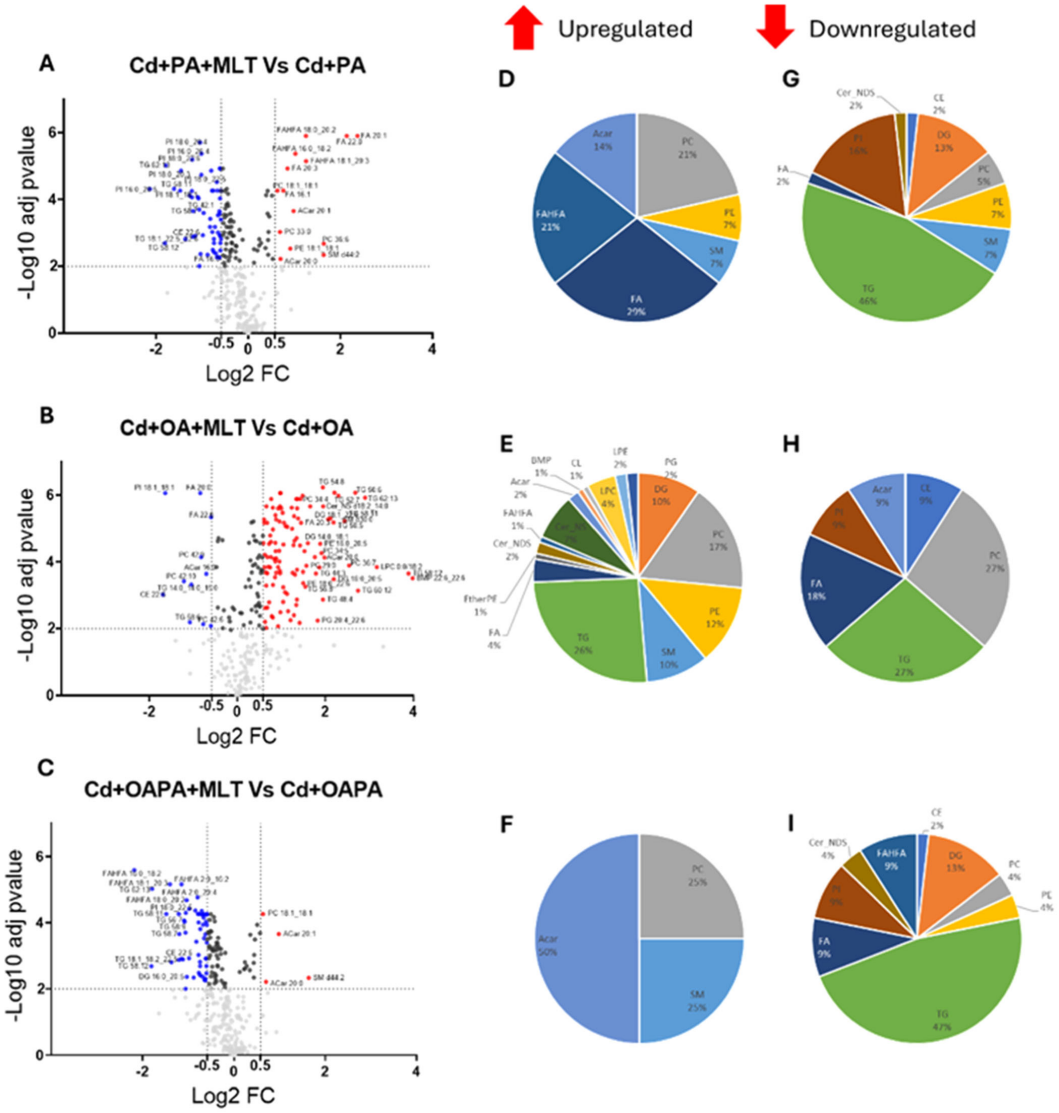
Differentially expressed lipids associated with the cytoprotective effect of MLT in HepaRG cells exposed to Cd and FFA‐induced lipotoxicity. (A‐C) Volcano plots of differentially expressed lipids (adj *p*value < 0.01. FC cutoff = 1.5) and pie charts of the corresponding lipid classes represented as significantly upregulated (D‐F) and downmodulated (G‐I) features. Legend to Volcano plot symbols: Blu = significantly downregulated features (FC < ‐ 1.5). Red = significantly upregulated features (FC > 1.5). Dark grey = significantly modified species with ‐ 1.5 < FC < 1.5. Light grey = non‐significantly modified species.

Moderated *t*‐test was used as univariate analysis method to identify specific changes of the cellular lipidome induced by the different lipotoxicity treatments and MLT cytoprotective effect (Figure 3, 4 and Supporting Information Tables S2‐4). The number of significantly modified lipids (adjusted *p* value < 0.01, FC = 1.5) and the top‐20 modulated species identified from pairwise comparisons are shown in Supporting Information Table S2 and S3‐4, respectively.

Characteristic changes of the cellular lipidome were identified for each treatment with the individual lipotoxicity agents Cd, PA and OA, as well as with MLT, and these changes apparently confirm those identified in the heatmap charts and multivariate analysis data presented earlier in section 3.2.1 and Figure 2.

**Figure 5:**
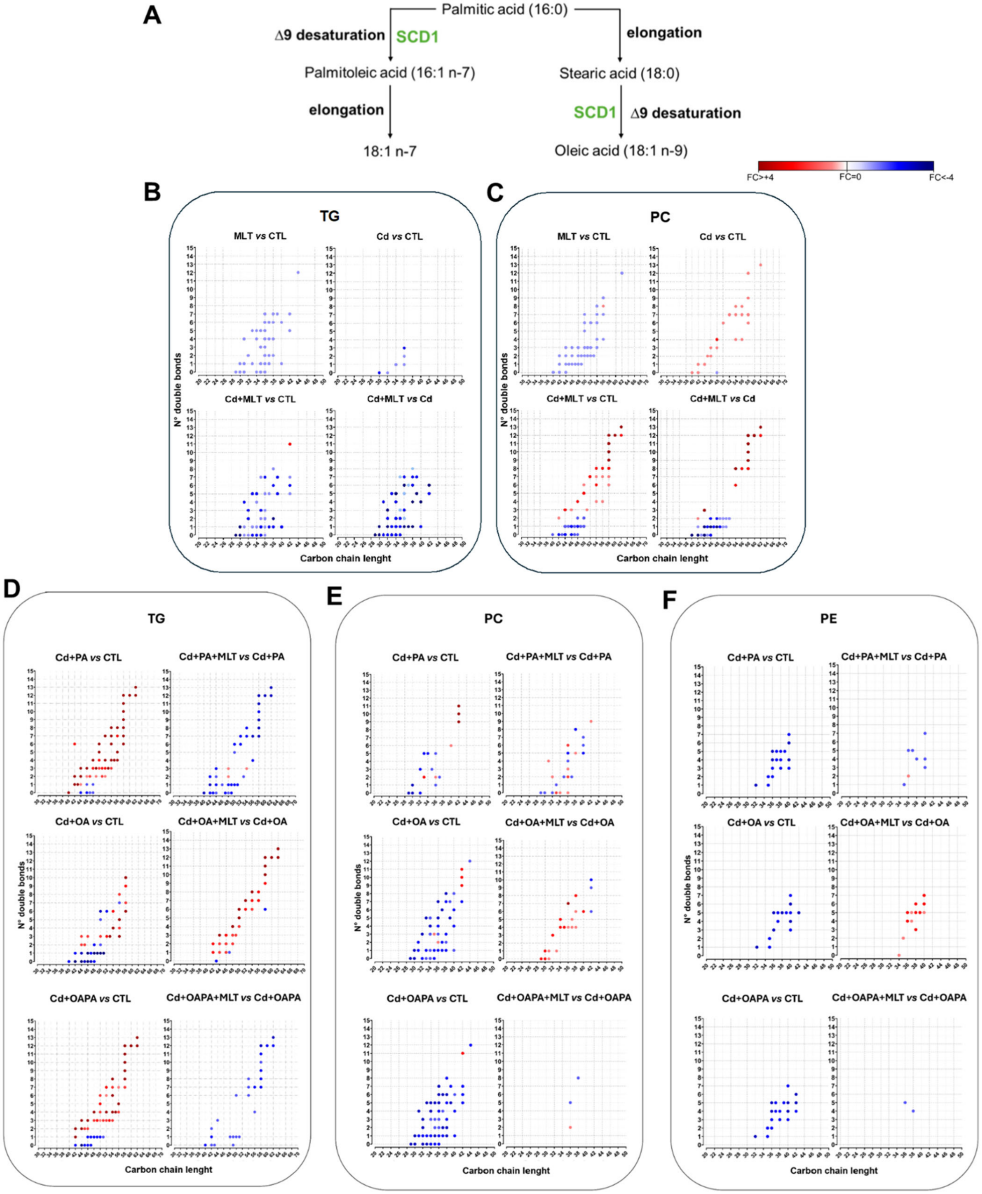
Effect of MLT on the carbon chain length and number of double bonds of acyl residues in triglycerides and main phospholipid species of HepaRG cells exposed to Cd and FFA‐induced lipotoxicity. (A) elongation and desaturation steps of FA metabolism involving PA and OA species. (B‐C) effect of MLT treatment on control and Cd‐exposed HepaRG cells. (D‐F) effect of MLT treatment on combined exposures to Cd and FFA‐induced lipotoxicity.

Significantly modified features are shown in the Volcano plot charts of Figure 3 and in Supporting Information Table S3; MLT treatment itself (i.e. in the absence of lipotoxicity agents) produced minor changes in the lipidome of HepaRG cells; these were represented by slight reductions (considering FC data shown in Figure 3A and Supporting Information Table S3A) in the differential expression of several metabolites (Supporting Information Table S2) in all the main lipid classes (Figure 3 ‐ pie charts), as well as of C chain length and total number double bonds in the acyl residues of the identified TG and PC features (Figure 5B‐C). Whereas, when Cd exposure was studied as individual treatment, mainly TG species were upregulated (16 features out of the top‐20 shown in Supporting Information Table S3D) along with some saturated ceramides and FAs, and these changes of the lipidome were associated with downregulations of several unsaturated ceramide, PS and FA species (Supporting Information Table S3C). The C chain length and number of double bonds were not affected in TG species, but increased to some extent in PC species (Figure 5B‐C).

Individual treatments with OA and OAPA induced remarkable upregulations of TG and diacylglycerols (DG), and the downregulation of phospholipids (mostly PC and PE) and sphingomyelins (SM), also modulating several minor species and classes of the cellular lipidome (Figure 3E, F and Supporting Information Table S3I‐L). Conversely, PA treatment showed the highest levels of differentially expressed lipids in the various classes (Supporting Information Table S2), but the most significant ones were mainly represented by downregulated TG species (Figure 3D and Supporting Information Table S3G); these features, and particularly the differential modulation of TGs, clearly distinguish the response of human hepatocytes' metabolism to this saturated FA from that of the monounsaturated OA, further highlighting differences in their lipotoxicity mechanisms [25].

##### 3.2.2.2 Combined Exposure to Cd and FFA Treatments

The combination of Cd and FFA treatments was associated with higher levels of differentially expressed features in the various classes of cellular lipids compared to individual treatments (Figure 3G‐I and Supporting Information Figure S3, Supporting Information Table S2 and S3M‐R).

Besides to TG class that showed the higher number of differentially expressed features, combinatorial treatments of Cd and FFA upregulated DG and Cer and downregulated SM, Acar, phospholipids (mainly PC and PE) and lisophospholipids [lysophosphocholines (LPC) and lysophosphoethanolamine (LPE)]. These changes provide a lipidomics signature to the lipotoxicity data presented earlier in section 3.1 and Figure 1, and replicate those observed in vivo in the lipidome of NAFLD patients' liver ([28, 29] and references therein).

However, Volcano plot (Figure 3G, H) and Venn diagrams data (Supporting Information Figure S3) identify specific effects of Cd exposure on the cellular lipidome when this alternatively combines with OA or PA treatments. In fact, Cd + OA and Cd + PA, similarly to the corresponding individual treatments with OA and PA, have in common a small number of differentially modulated lipid metabolites (Supporting Information Figure S3). More in detail, all the top‐20 upregulated lipids in Cd+PA treatment were TG species (with absolute log2 FC values ranging from 5.8 to 2.9; Figure 3G, H and Supporting Information Table S3M, N), while only 9 features of Cd+OA treatment were TG (with log2 FC values ranging from 2.7 to 1.2; Supporting Information Table S3O, P), and the relative abundance of features with higher number of double bonds and carbon chain length increased in the presence of PA, particularly in TG species, whereas in OA treatment the intensity of both TG and PC features with lower double bond number and chain length decreased (Figure 5D‐E). These results describe the combination of Cd and PA exposure as critical for the lipid metabolism of human hepatocytes, leading to excess TG and LC‐FA accumulation. These defects can be explained by the reduced availability of the cellular pool of FAs to β‐oxidation processes and PL metabolism to maintain an optimal energy status and membrane composition of the cell, which are characteristic cues of NAFLD (vide infra).

##### 3.2.2.3 MLT Effects on Cd Exposure and Its Combination With FFA Treatments

As a general effect, MLT treatment modified and partially reverted most of the changes that Cd exposure produced on the cellular lipidome as either individual event (Figure 3C) or combined with FFA treatments (Figure 4 and Supporting Information Table S4). In the individual exposure to Cd, MLT increased the capability of the cells to accumulate TG (Figure 3C), which can be protective in lipotoxicity [25], also increasing the relative abundance of lipid features bearing a lower number of double bonds and length of the C chain (Figure 5B). Again, PCs containing very long‐chain (VLC) and polyunsaturated FA markedly increased by the effect of MLT treatment on Cd exposure, while the expression of PC residues with lower number of double bonds and chain length decreased (Figure 5C). Furthermore, Volcano plot data demonstrate that MLT downregulates the same classes of lipids either in the presence or absence of Cd exposure (Figure 3A, C), and these classes differed from those affected by the individual exposure to Cd as far as the type of metabolites involved and expression levels are concerned (comparison with Figure 3B). Together these results demonstrate that the repairing effect of MLT in human hepatocyte exposed to Cd toxicity involves an enhanced metabolism of FA and TG synthesis. The importance of these effects in preventing lipotoxicity through LD formation is well established [17, 25].

Moreover, specific changes of the cellular lipidome differentiated the cytoprotective effect of MLT when Cd exposure was combined with PA and OA (Figure 4 and Supporting Information Table S4). In the Cd + OA model, MLT induced a higher number of upregulated features compared to Cd + PA and even to Cd+OAPA lipotoxicity treatments (Figure 4B vs 4A, C, respectively); these included species of TG (mainly composed of polyunsaturated long‐chain FA), PL and SM classes (Figure 4B, E, H vs 3A, D, G respectively, Supporting Information Table S1B, Table S2); also, MLT had a remarkable induction effect on the relative intensity of acyl residues at the different levels of C chain length and number of double bonds, especially the highest ones in the case of TG (right panels of Figure 5D‐F). These findings highlight a synergistic effect of MLT and OA in “repairing” the lipid metabolism and the cell damage of Cd exposed hepatocytes by the enhanced availability of substrates for lipid biosynthesis pathways and energy metabolism; these effects are of potential therapeutic relevance in hepatic lipotoxicity, and apparently, PA interferes with them.

Other characteristic aspects of this MLT effect identified by univariate analysis in Cd+OA treatment, included BMP 22:6/22:6 upregulation (Supporting Information Table S4B); according to SPL‐DA multivariate analysis data shown in Figure 2F, this was the top‐ranking upregulated feature that characterize the response to MLT treatment in Cd‐exposed hepatocytes.

Furthermore, in Cd+PA treatment, i.e. the condition that in our experimental model produced the highest levels of lipotoxicity (Figure 1), MLT significantly decreased DG and the PL species PI, while upregulated mainly FA, fatty acyl esters of hydroxy fatty acid (FAHFA) and some PL and SL features characterized by the presence of unsaturated residues (Figure 4A, D, G and Supporting Information Table S4A). This increased abundance of unsaturated FA residues is a response to MLT treatment that was invariably observed in the upregulated lipid metabolites of both the Cd+PA and Cd+OA treatments (Supporting Information Tab. S4A and S4B, respectively), and these results are in agreement with chain length and unsaturation data presented earlier and in Figure 5.

Together these findings suggest that the repairing effect of MLT may depend on elongase and acyl‐desaturase steps of FA metabolism that are typically impaired in hepatocyte lipotoxicity to sustain immunometabolic and inflammatory complications of NAFLD [30].

#### 3.2.3 Pathway Analysis of MLT Cytoprotective Function

**Figure 6:**
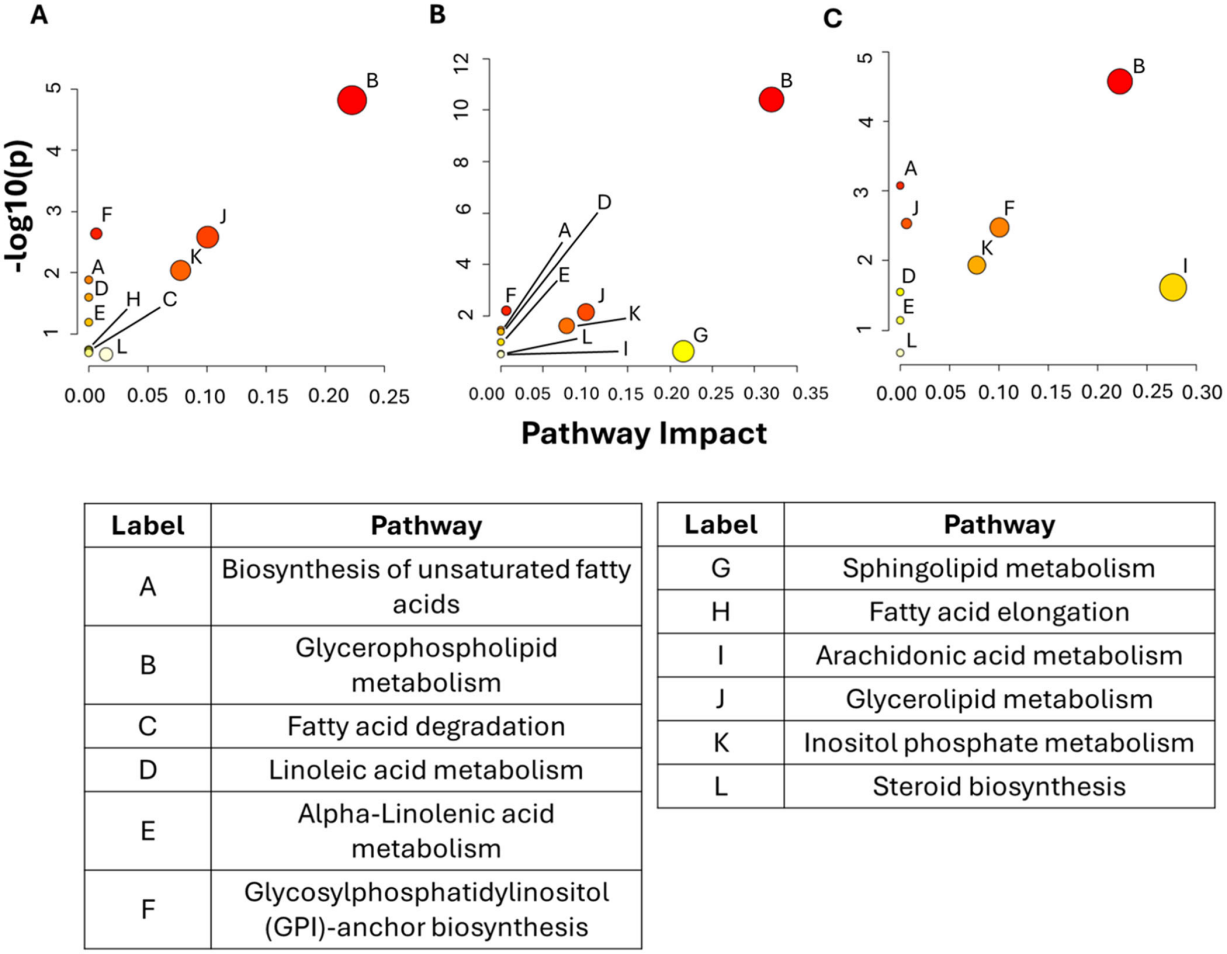
KEGG lipid pathways modulated by MLT in combinatorial (Cd + FFAs) lipotoxicity treatments. KEGG pathways significantly involved in the cytoprotective effect of MLT in combinatorial lipotoxicity treatments (A. Cd+PA, B. Cd+OA, C. Cd+OAPA) are represented by dots plot chart according to pathway impact (x axis) and *p*‐value (y axis) data of the analysis model.

Significantly modulated lipids by MLT were evaluated through data enrichment analysis to further explore the repairing and cytoprotective mechanism of this molecule in Cd and FFA‐induced hepatocyte lipotoxicity. KEGG lipid pathways associated with MLT effect (Figure 6) included glycerophospholipid metabolism that showed the greatest impact and significance in the three lipotoxicity models investigated in this study (namely Cd+PA, Cd+OA, and Cd+OAPA), followed by the biosynthesis of unsaturated fatty acids, metabolism of linoleic and alpha‐linoleic acids, glycerolipid and steroid metabolism, and inositol phosphate metabolism. Minor impact and lower significance were observed for FA degradation and elongation pathways that discriminated the effect of MLT in Cd+PA treatment from those of other lipotoxicity treatments (Figure 6A), for arachidonic acid metabolism that was involved in Cd+OA and Cd+OAPA (shown in Figure 6B and 6C, respectively), and then for sphingolipid metabolism that was significantly modulated by MLT in Cd+OA treatment only (Figure 6B).

**Figure 7:**
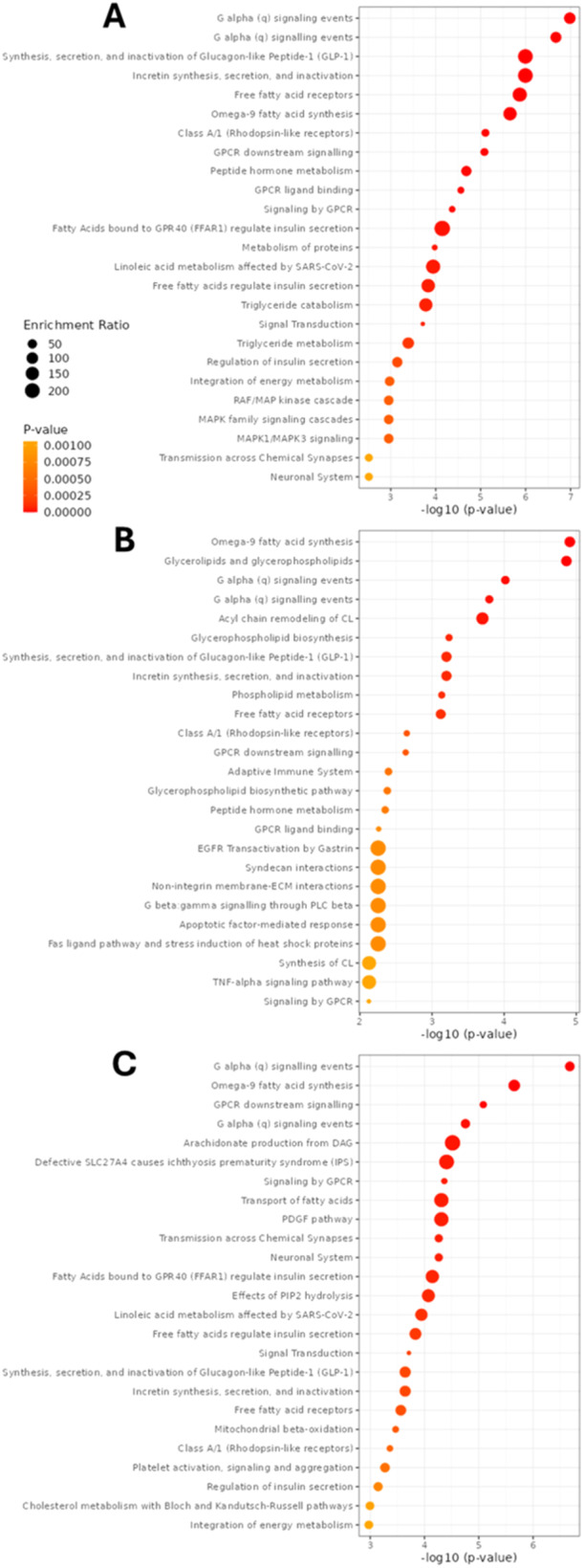
Biological functions identified by enrichment analysis as associated with the cytoprotective effect of MLT in Cd and FFA‐induced lipotoxicity of HepaRG cells. Lipotoxicity treatments were (A) Cd+PA, (B) Cd+OA and (C) Cd+OAPA (performed according to the experimental protocol of Figure 1A). The dot plot chart presents the significance (*p*‐value) of the associations by incremental colour scale and distribution through the abscissa, while the symbol size represents the enrichment ratio according to figure legend values.

Biofunctions and cellular pathways significantly associated with the cytoprotective effect of MLT in Cd and FFA‐induced lipotoxicity are shown in Figure 7 and included among the top‐ranking different G alpha signalling annotations (namely, G alpha signalling events, GPCR ligand binding and downstream signalling, and signalling by GPCR), omega‐9 fatty acid synthesis, insulin metabolism and function, and the synthesis, secretion and inactivation of incretin and glucagon‐like peptide 1, as well as different annotations associated with FA, TG and energy metabolism regulation.

#### 3.2.4 SCD1 Activity Influences the Repairing Effect of MLT on FA Metabolism of HepaRG Cells Exposed to Cd and PA Lipotoxicity

**Figure 8:**
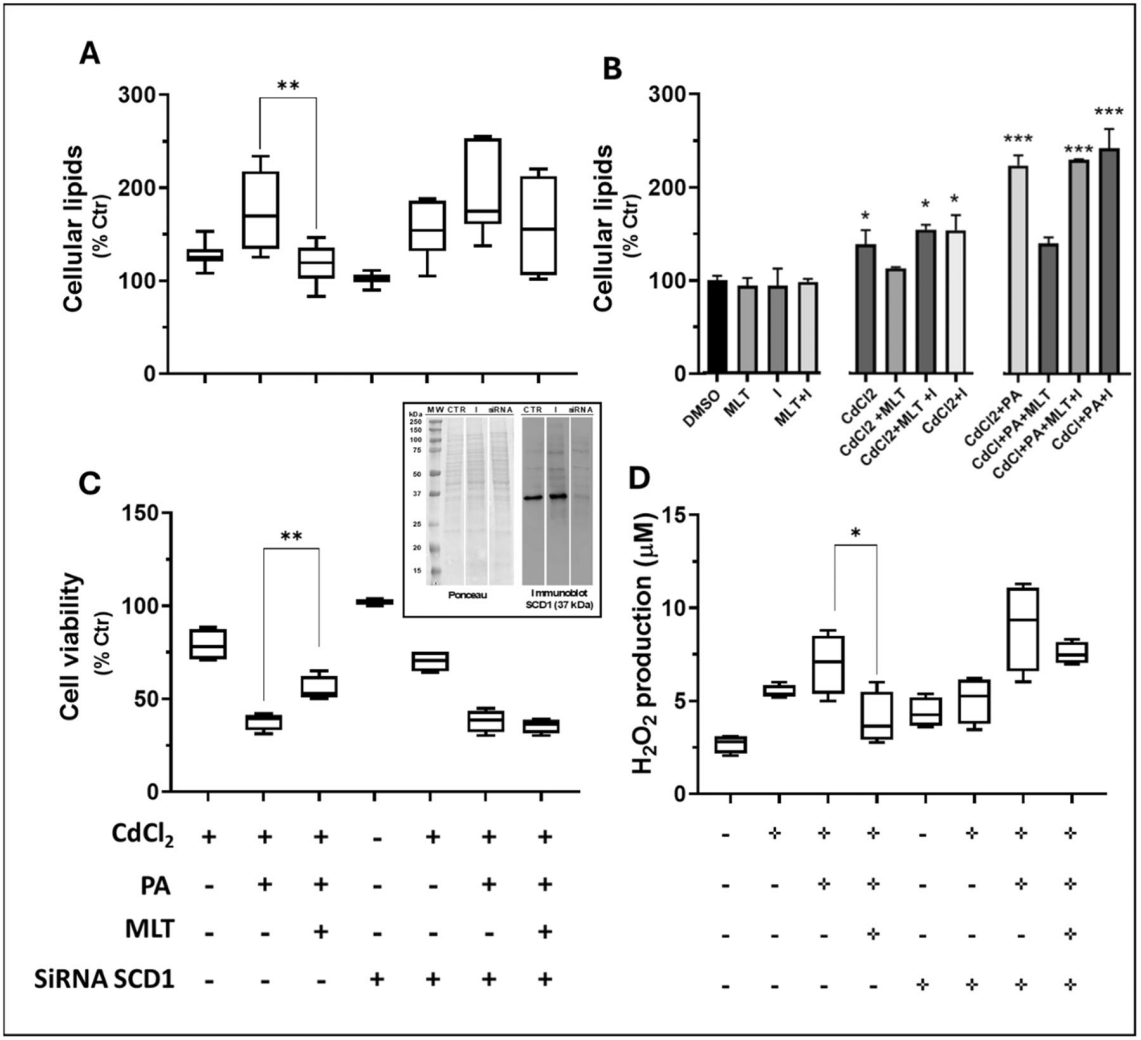
MLT repairing and cytoprotective properties in Cd and PA‐induced lipotoxicity of HepaRG cells depend on SCD1 activity. (A, B) The role of SCD1 (or Δ9‐desaturase) on the fat build up response to Cd and PA exposure (measured by Oil Red‐O staining) was investigated using a siRNA procedure for transient inhibition of *SCD1* gene expression and the pharmacological inhibitor of SCD1 enzymatic activity A‐939572 (identified with the label “I” in chart B). (C, D) The effect of *SCD1* silencing was also studied on the lipotoxicity indicators cell viability and H_2_O_2_ production levels. The efficacy of the siRNA transfection protocol was verified by immunoblot (insert to panel C).

Lipidomics data (sections 3.2.1–2.3) suggest that Cd and its combination with PA exposure impair FA biosynthesis in HepaRG cells, which is repaired by MLT. To verify these data we studied the first and critical desaturation step in the biosynthesis of LC and VLC FA, namely SCD1 enzymatic activity (Figure 5A); SCD1 activity is crucial for the saturated 16 C atom species PA to enter the elongation and desaturation steps that promote its conversion to the 18:1 species OA for the utilization in n‐9 FA biosynthesis or β‐oxidation processes of the cell, or alternatively for the biosynthesis of n‐7 FA [24, 31]. The results demonstrated that both the gene silencing and pharmacological inhibition of SCD1 activity interfere with the effect of MLT in reducing the fat build up response to Cd and PA exposure (Figure 8A, B); *SCD1* gene silencing also reduced the efficacy of MLT in preventing Cd+PA‐induced lipotoxicity (Figure 8C, D). The Oil Red O staining images corresponding to these data are shown in Supporting Information Figure S4.

## 4 Discussion

This study first reports on the changes of the cellular lipidome that MLT induces during its repairing function against Cd‐induced lipotoxicity in human hepatocytes [20]. Since hepatocyte lipotoxicity is central to NAFLD aetiology [17], this repairing effect of MLT may hold great therapeutic potential.

Cd exposure induces fat build‐up and lipotoxicity effects in HepaRG hepatocytes by increasing H_2_O_2_ production and the apoptotic signalling of these cells (Figure 1). Changes of the cellular lipidome were also observed (Figure 3 and Supporting Information Table S3), which resemble those found in the liver of mice exposed to Cd toxicity [28] and NAFLD patients [29].

Important enough was the observation that both the overall steatogenic effect and the specific changes of the cellular lipidome consequent to Cd exposure were significantly corrected by MLT, leading to an enhanced metabolism of cellular FA, particularly through elongation and unsaturation processes, and TG synthesis. Along with lipid droplet formation, these lipid metabolism effects are known to help preventing lipotoxicity [17, 24, 25, 27]. These results and the fact that differentially expressed lipids in MLT treatment itself are represented by a small number of slightly downregulated features (Figure 3 and Supporting Information Table S3), pinpoint this molecule in the category of physiological modulators of lipid metabolism and stress relievers of the hepatocyte.

Lipidomics data and the cellular hallmarks investigated in this study unequivocally demonstrate a synergistic effect of Cd exposure and FFA treatments in inducing steatosis and lipotoxicity in human hepatocytes. In this context, PA promoted a worse scenario compared to OA. These FAs are physiological nutrients and endogenous metabolites with key role in hepatocyte lipotoxicity and fatty liver disease [21, 24, 31] that cannot be disregarded during Cd toxicity studies in liver cells. However, saturated and unsaturated FFA species, such as PA and OA, have different capability to induce lipotoxicity [24, 32, 33] and in this respect our results demonstrated that combining Cd with FFA, particularly with the saturated species PA, markedly altered the cellular lipidome ultimately turnig hepatocyte steatosis from a relatively benign fat storage process – that is, that favoured by OA treatment (Figure 1) ‐ to a stressful and eventually cytopathic condition of lipotoxicity, i.e. a condition characterized by an increased flux of reactive oxygen species, such as H_2_O_2_, and apoptotic death (Figure 1) [20]. In the clinical frames of NAFLD, this may reduce the liver cell mass and its function, thus contributing to liver failure (discussed in detail elsewhere [17]). Cd+OA and Cd+PA treatments showed characteristic and markedly different lipidomic signatures; likewise individual treatments with OA and PA, these two combinatorial treatments had in common few differentially modulated lipid metabolites (Supporting Information Figure S3) and upregulated lipids in Cd+PA treatment were essentially TG species with higher number of double bonds and carbon chain length; apparently, these TG species did not provide sufficient protection to the cell once stored in lipid droplets [25]. Conversely, Cd+OA treatment produced a lower upregulation of TG species compared to Cd+PA, and the intensity of both TG and PC features with lower double bond number and chain length decreased (Figure 5D‐E). These results and cell lipotoxicity data (Figure 1) identify the combination of Cd with PA exposure as a critical stressogenic event for the human hepatocytes, while OA appears to alleviate Cd toxicity increasing the availability of cellular FAs for cell protection processes; that may include β‐oxidation and energy production processes, lipid biosynthesis pathways, PL metabolism and membrane homeostasis [24, 31].

In this respect, both the lipidome repairing and cytoprotective effects of MLT in Cd exposure are favoured by OA, which provides a characteristic lipid signature and correction of FA metabolism during MLT treatment (Figure 4 and 5D‐F). Lipidomics data indicate that OA may assist MLT in modulating the cytoprotective modification of acyl residues composition in TG and PL species allowing optimal levels of chain length and number of double bonds (Figure 5D‐F); this provides components to restore membrane lipids and energy substrates to respond to stress stimuli and to control homeostatic processes, including the flux of mitochondrial substrates and ROS production [34, 35]. Conversely, PA appears to interfere with the MLT repairing and cytoprotective functions. This saturated species needs specific desaturation and elongation steps to enter the cell metabolism with formation of other metabolites as stearic acid (SA; 18:0), OA (18:1n‐9), or palmitoleic (16:1n‐7) and *cis*‐vaccenic acid (18:1n‐7) (Figure 5A); apparently, these processes expose the cell to an excess of long‐chain saturated lipids, such as the same PA and its derivative SA, which trigger the stress response and death processes of the liver cell (reviewed in [17, 24, 25, 32, 33]) (Figure 1).

Data analysis and interpretation of lipidomics results identified lipid pathways significantly associated with these MLT effects in Cd and FFA induced lipotoxicity (Figure 6); the former and apparently more significant association was that with glycerophospholipid metabolism, which is reportedly central in the Cd‐induced alterations of lipid metabolism at the hepatic and systemic levels [28, 36], followed by the biosynthesis of unsaturated fatty acids, and the metabolism of linoleic and alpha‐linoleic acids, of glycerolipids, steroids, and inositol phosphate. It is worth noting that minor impacts and levels of significance, but higher discrimination potential among lipotoxicity treatments, were observed for the annotation “FA degradation and elongation pathways” that discriminates the effect of MLT in Cd+PA treatment from that observed in other lipotoxicity treatments, also providing a straight interpretation to the experimental results on C chain length and number of double bonds shown in Figure 5; whereas sphingolipid metabolism was significantly modulated by MLT in Cd+OA treatment only.

More suggestive of the therapeutic potential of MLT were the biofunctions identified by enrichment analysis (Figure 7). These included GPCR ligand binding and downstream signalling as first annotation. G protein‐coupled receptors have bile acids and FFA as main ligands and have consistently been described to play a key role in NAFLD and metabolic diseases, as well as in liver fibrosis (reviewed elsewhere in [37]); omega‐9 fatty acid synthesis was another significant biofunction, which is in agreement with the observed restoration effect of MLT on the PA‐induced alterations of the cellular lipidome characterized by a better metabolism of this saturated species through the elongation and desaturation steps of FA metabolism [31] (Figure 4 and 5). Others included insulin metabolism and function, and the synthesis, secretion and inactivation of incretin and glucagon‐like peptide 1, which may support a role for MLT in the clinical management of NAFLD and as ancillary treatment of its metabolic complications that characterize metabolic‐NAFLD or MAFLD [38].

Since lipidomics data highlighted a key role of FA metabolism in the repairing effect that MLT exerts on the lipidome of human hepatocytes exposed to Cd and PA lipotoxicity, specific studies were performed to verify the involvement of SCD1 enzymatic activity in this effect. This enzyme catalyzes the first desaturation step of FA biosynthesis, which represents the point of entrance for PA into the elongation and desaturation processes of FA metabolism [31, 39] (Figure 5A). Preliminary evidence in literature identified an induction response of the *SCD1* gene in hepatocarcinoma HepG2 cells treated with micromolar dosages of MLT during the exposure to an excess of OA [40]. Our findings in non‐tumoral HepG2 hepatocytes exposed to low nanomolar concentration of MLT do not confirm this induction response (not shown), but the transient inhibition of *SDC1* expression by siRNA transfection interfered with MLT efficacy in correcting cellular steatosis and lipotoxicity indicators of Cd + PA exposed cells (Figure 8). Also, the pharmacological inhibition of SDC1 enzymatic activity hindered the effect of MLT on cellular steatosis. These results provide preliminary but solid evidence of a causal role of SCD1 enzyme activity in the repairing mechanism of MLT in human hepatocytes, which surely deserves further characterization.

In conclusion, Cd synergizes with FFAs, and particularly with the saturated species PA, to induce hepatocyte lipotoxicity; this toxicity effect was associated with changes of the cellular lipidome resembling those reported in literature for experimental and human NAFLD. MLT repairs these alterations of the lipidome In Vitro, which may hold therapeutic potential in Cd exposure and hepatocyte lipotoxicity.

## Author Contributions

Anna Migni, Desirée Bartolini and Francesco Galli conceived and designed the study and drafted the initial manuscript. Anna Migni, Desirée Bartolini, Giada Marcantonini, Alessia Tognoloni and Maria Rachele Ceccarini performed most of the experiments and data processing. Francesco Galli, Roccaldo Sardella and Mario Rende reviewed and revised the paper. All authors approved the final manuscript as submitted.

## Conflicts of Interest

The authors declare no conflicts of interest.

## Supporting information

Supplementary_material_REVISION2.

## Data Availability

The data that support the findings of this study are available from the corresponding author upon reasonable request.
