# Supplementary material for: Melatonin Repairs the Lipidome of Human Hepatocytes Exposed to Cd and Free Fatty Acid‐Induced Lipotoxicity
Source: J Pineal Res. 2025 Apr 7;77(3):e70047. doi: 10.1111/jpi.70047 (PMC11975211; doi:10.1111/jpi.70047)
Supplement: Supplementary file 1 — Supplementary_material_REVISION2. [file JPI-77-e70047-s001.pdf]

Supplementary charts

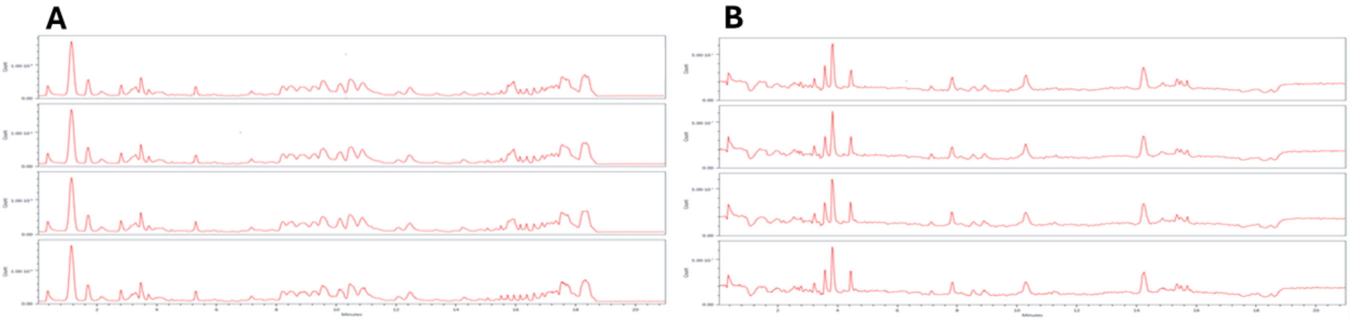

Suppl. Figure S1. Total Ion Current (TIC) chromatograms of QC sample acquired in positive and negative ionization modes.
TIC chromatograms of QC sample acquired in positive (A) and negative (B) ionization mode.

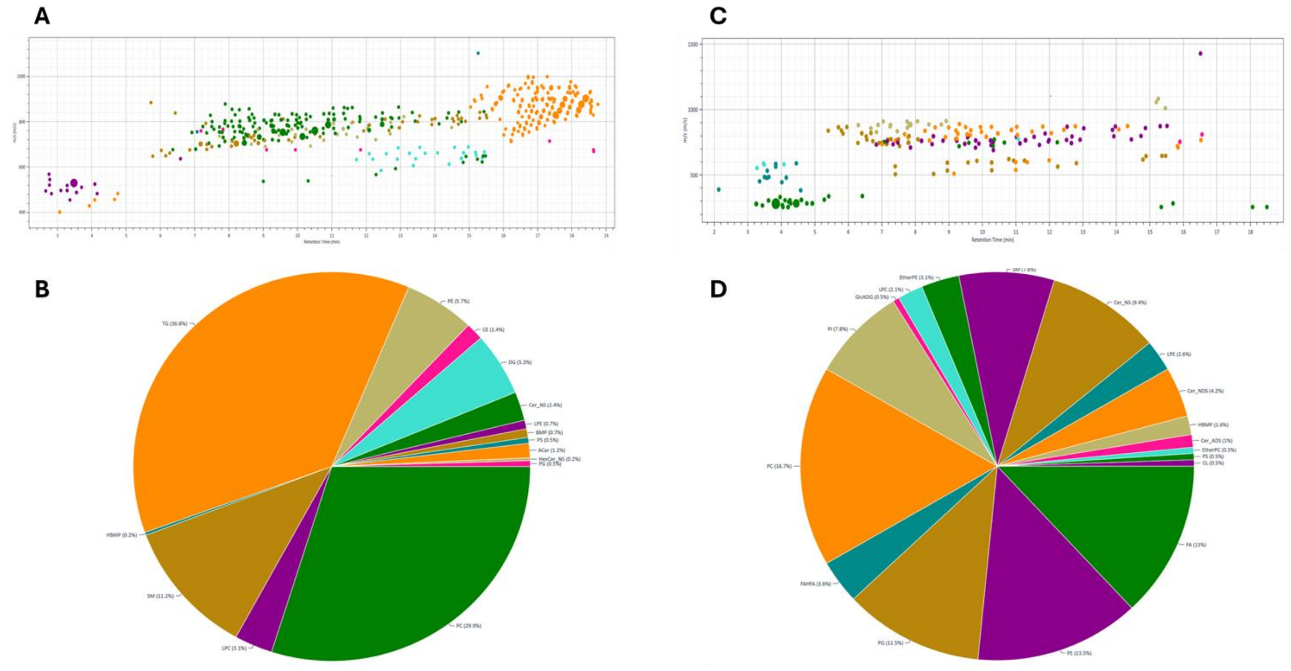

Suppl. Figure S2. Kendrick plots and pie charts of fully identified lipid species and classes in QC sample.
Identified features in QC sample. shown in different colours according to lipid class belonging. acquired in positive (A, B) and negative (C, D) ionization mode are visualized by a mz/Rt Kendrick plot and pie chart.

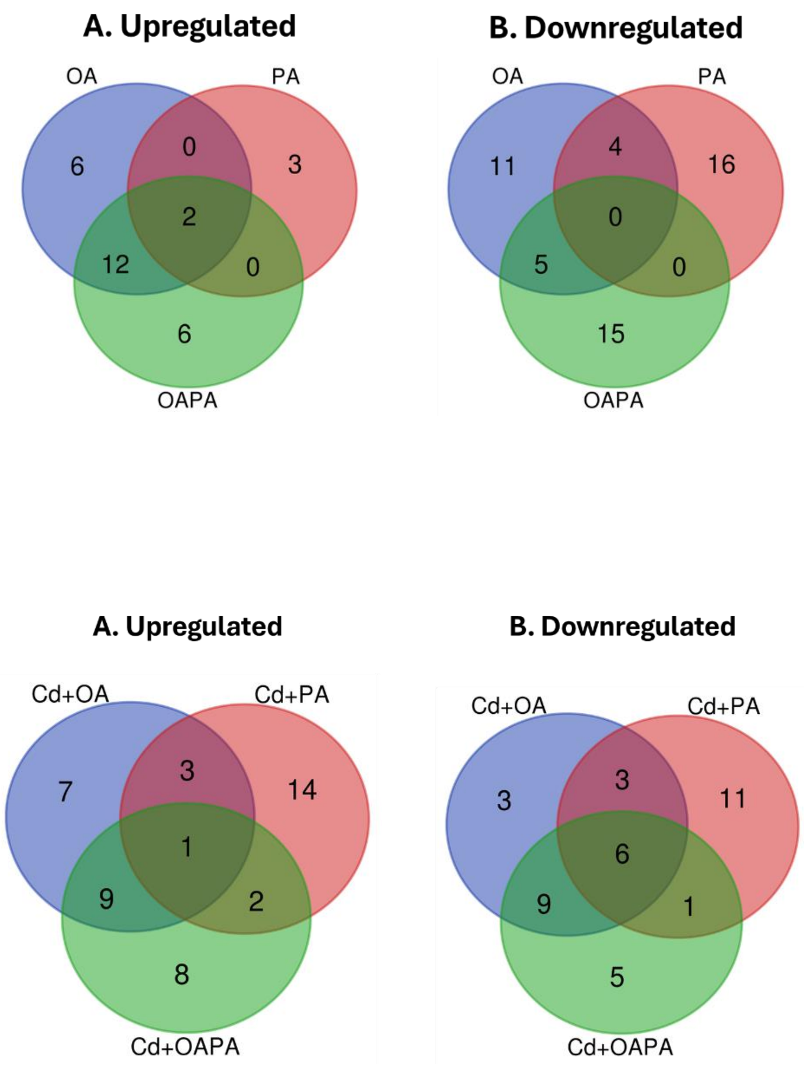

Suppl. Figure S3. Venn Diagrams of significantly TOP 20 modified lipid classes in individual FFAs treatments and Cd+FFA combinatorial treatments. Venn diagrams show the concurrence in the top-20 significantly upregulated and downregulated lipid features FFAs treatments (upper charts: red= PA Vs CTL, blue=OA Vs CTL, green=OAPA Vs CTL) or Cd+FFA combinatorial treatments (lower charts: red= Cd+PA Vs CTL, blue=Cd+OA Vs CTL, green= Cd+OAPA Vs CTL) as resulting from the comparison with control cells (treated with the vehicle DMSO only).

Suppl. Table S1. Identified lipids ranked by lipid class

Compound	CTL	MLT	OA	PA	Cd	OAPA	Cd_OA	Cd_PA	Cd_OAP	Cd_MLT	d_OA_MI	Cd_PA_MLT	Cd_OAP	Lipid Clas	Mass	Retention Time	Ion Species
ACar 16:0	-1.229983	-1.959084233	-4.5937	-2.3681	-0.9834	-2.6554	-3.8613	-2.2895	-5.3342	-2.8025	-4.5657	-3.0684325	-5.6954	ACar	399.3343	3.0599992	[M*]+ [M+H]+ [M+K]+ [M+NH4]+ [M+Na]+
ACar 18:0	-1.03964488	-1.963779433	-5.4176	-2.8062	-1.8748	-2.4409	-4.4143	-2.5454	-5.255	-3.037	-3.7323	-3.0790475	-5.3423	ACar	427.3658	3.9219987	[M*]+ [M+H]+ [M+K]+ [M+NH4]+ [M+Na]+
ACar 20:1	-2.975971933	-3.815937067	-4.6166	-4.6857	-3.8109	-2.2278	-4.0502	-4.609	-6.6511	-5.2164	-3.7535	-3.9967925	-5.6675	ACar	453.381	4.0749984	[M+H]+ [M+K]+ [M+NH4]+ [M+Na]+
ACar 20:0	-3.2326794	-4.3141168	-8.8294	-5.9219	-4.8906	-4.6049	-7.9243	-5.4548	-8.1578	-5.5285	-5.9435	-5.2470818	-7.4558	ACar	455.3963	4.6510005	[M+H]+ [M+K]+ [M+Na]+
BMP 18:1_22:6	-3.061583833	-3.685989367	-3.9018	-3.9906	-2.8151	-2.7805	-4.8869	-4.8111	-5.9609	-5.6591	-4.2866	-5.6484616	-6.1792	BMP	820.5238	6.410999	[M*]+ [M+H]+ [M+K]+ [M+NH4]+ [M+Na]+
BMP 22:6_22:6	-3.046896067	-3.6061649	-5.117	-3.8034	-2.9923	-4.0764	-8.7642	-4.3541	-6.605	-4.4032	-4.78	-4.297671	-7.5564	BMP	866.5078	5.6979985	[M*]+ [M+H]+ [M+K]+ [M+NH4]+ [M+Na]+
CE 22:6	-2.140000067	-1.705591833	-1.3782	-0.9709	-1.2441	-1.9535	-0.6433	-1.1876	-2.5574	-1.9488	-2.3343	-1.906592	-3.7613	CE	696.5815	17.354998	[M+H]+ [M+K]+ [M+NH4]+ [M+Na]+
Cer_NDS d18:0_16:0	0.016524633	-0.764618557	-1.2313	-0.7126	0.0412	-0.9046	-0.7669	0.7172	-0.4937	-0.3729	-0.1571	-0.0719884	-1.1821	Cer_NDS	539.5289	11.347994	[M+CH3COO]+ [M+HCOO]+ [M-H]-
Cer_NDS d18:0_18:0	-1.975837067	-2.773345967	-1.994	-2.4804	-1.2264	-2.3977	0.5264	0.9572	-0.2061	0.7189	0.9659	1.22211077	-0.6218	Cer_NDS	567.56	12.984992	[M+CH3COO]+ [M+HCOO]+ [M-H]-
Cer_NDS d18:0_22:0	-0.877966567	-1.6102492	-1.4146	-1.4492	-0.5099	-1.9487	0.03	0.8017	-0.4176	-0.1245	0.2957	0.60323779	-1.0526	Cer_NDS	623.6221	15.489007	[M+CH3COO]+ [M+HCOO]+ [M-H]-
Cer_NDS d16:0_26:2	2.0253989	1.125182467	0.1255	1.303	1.7931	0.9914	-0.4526	0.8248	-0.6046	-0.3648	0.2311	0.61985843	-0.9889	Cer_NDS	647.6234	15.448	[M+CH3COO]+ [M+HCOO]+ [M-H]-
Cer_NDS d18:0_24:0	-0.482421253	-1.2320633	-0.9191	-0.8565	-0.3707	-1.9885	0.3744	0.7959	-0.4616	0.1377	0.8659	0.36467552	-1.0081	Cer_NDS	651.6543	15.807005	[M+CH3COO]+ [M+HCOO]+ [M-H]-
Cer_NDS d19:0_24:0	-0.3996067	-1.103128433	-0.8171	-0.7354	-0.3025	-1.8307	0.4485	0.8362	-0.333	0.1834	-0.6244	-1.0267366	-0.9269	Cer_NDS	665.6694	15.807005	[M+CH3COO]+ [M+HCOO]+ [M-H]-
Cer_NS d18:2_14:0	-1.502790433	-2.265904733	-2.8219	-2.4384	-1.4904	-1.8172	-5.1952	-2.928	-4.7898	-4.6979	-3.2423	-3.3544801	-4.2201	Cer_NS	507.4658	7.418	[M+CH3COO]+ [M+HCOO]+ [M-H]-
Cer_NS d16:1_16:0	-0.073835374	-0.89738528	-0.8923	-0.7351	0.0959	-0.474	-1.8546	-0.5206	-1.8984	-1.9198	-0.8602	-1.0626965	-1.8585	Cer_NS	509.4817	8.566001	[M+CH3COO]+ [M+HCOO]+ [M-H]-
Cer_NS d17:1_16:0	-0.514029187	-1.269485467	-1.2164	-0.9874	-0.243	-0.8264	-1.3963	-0.2881	-1.4487	-1.5621	-0.5248	-0.4405378	-1.6472	Cer_NS	523.4976	9.402005	[M+CH3COO]+ [M+HCOO]+ [M-H]-
Cer_NS d18:2_16:0	2.710838933	1.832805633	1.1106	1.9193	2.8048	2.0777	0.3659	1.9305	0.6718	0.5548	1.3401	1.42292847	0.5473	Cer_NS	535.4978	8.974	[M+CH3COO]+ [M+HCOO]+ [M-H]-
Cer_NS d18:1_16:0	2.054364533	1.383946733	1.9052	1.8429	2.3693	2.1789	1.8982	2.7867	1.8843	1.4316	2.4273	2.30644927	1.8038	Cer_NS	537.5135	10.299003	[M+CH3COO]+ [M+HCOO]+ [M-H]-
Cer_NS d18:2_17:0	-2.799972533	-3.377826067	-3.1152	-3.0721	-2.5788	-2.8095	-3.6296	-2.7716	-4.0056	-4.2298	-2.884	-3.0397955	-4.0478	Cer_NS	549.5129	9.858001	[M+CH3COO]+ [M+HCOO]+ [M-H]-
Cer_NS d19:1_16:0	-1.729218167	-2.432565067	-3.3657	-1.903	-1.3372	-2.1625	-3.6096	-0.992	-2.1948	-2.2562	-1.9594	-1.3152459	-2.4231	Cer_NS	551.5288	10.941995	[M+CH3COO]+ [M+HCOO]+ [M-H]-
Cer_NS d18:2_18:0	-0.09483274	-0.80484708	-2.0515	-1.5696	-0.1128	-0.5101	-1.8095	-0.5296	-1.7605	-1.3728	-1.1894	-0.7206631	-1.8615	Cer_NS	563.5288	10.799996	[M+CH3COO]+ [M+HCOO]+ [M-H]-
Cer_NS d18:1_18:0	-0.571379973	-1.2094402	-1.7021	-1.5371	-0.1803	-0.4972	-0.8624	0.2904	-0.6292	-0.6861	-0.313	0.20062319	-0.7667	Cer_NS	565.5446	12.252997	[M+CH3COO]+ [M+HCOO]+ [M-H]-
Cer_NS d18:2_20:0	-0.78514735	-1.6132005	-3.8959	-2.1623	-0.614	-0.998	-3.8965	-1.3633	-2.8116	-2.3415	-2.1838	-1.6254997	-3.1662	Cer_NS	591.56	12.8099985	[M+CH3COO]+ [M+HCOO]+ [M-H]-
Cer_NS d16:1_22:0	-0.504519143	-1.404293067	-2.7921	-1.696	-0.2722	-1.5808	-2.4308	-0.6931	-2.0211	-1.9161	-1.8032	-0.8450699	-2.3036	Cer_NS	593.5754	14.329001	[M+CH3COO]+ [M+HCOO]+ [M-H]-
Cer_NS d18:2_22:0	-2.610943467	-3.380255367	-6.0466	-3.9347	-2.607	-3.491	-6.1793	-3.4412	-5.6097	-5.049	-5.3657	-4.4297237	-6.1229	Cer_NS	619.5898	14.823999	[M*]+ [M+H]+ [M+K]+ [M+NH4]+ [M+Na]+
Cer_NS d18:1_22:0	-3.975542067	-4.7290986	-5.4295	-4.617	-3.7812	-4.7619	-4.9135	-3.5632	-4.9355	-5.7397	-4.4868	-4.6949107	-5.3249	Cer_NS	621.6046	15.381999	[M*]+ [M+H]+ [M+K]+ [M+NH4]+ [M+Na]+
Cer_NS d18:2_23:0	-1.459650667	-2.354164767	-2.6439	-2.2418	-1.5258	-2.4359	-3.2061	-2.0857	-3.4264	-3.3463	-2.7447	-1.9825656	-4.4492	Cer_NS	633.6072	15.213006	[M+CH3COO]+ [M+HCOO]+ [M-H]-
Cer_NS d18:2_24:1	-2.164454133	-3.008728667	-3.001	-2.9456	-2.2685	-2.0288	-4.8456	-3.1542	-4.7701	-4.8326	-4.0055	-3.9109383	-5.1928	Cer_NS	645.6048	14.911004	[M*]+ [M+H]+ [M+K]+ [M+NH4]+ [M+Na]+
CL 80:10	-2.5916144	-2.8947983	-3.5197	-3.1143	-2.6245	-3.072	-3.8453	-3.0154	-3.7866	-3.0905	-3.1207	-2.9993083	-4.3008	CL	1557.0754	11.3099985	[M+CH3COO]+ [M-H]-
DG 14:0_18:1	-3.5561886	-3.751910533	-3.5911	-3.7712	-3.2667	-3.3267	-5.7458	-4.9224	-4.5358	-4.1367	-4.1436	-4.3703187	-5.1037	DG	566.4897	12.405	[M+H]+ [M+K]+ [M+NH4]+ [M+Na]+
DG 16:0_18:1	-0.55460231	-1.2629242	-0.7643	-1.5372	-0.5208	-0.1599	-1.8288	-1.5634	-1.7795	-2.2233	-0.5859	-1.8811709	-2.2047	DG	594.5211	14.420005	[M*]+ [M+H]+ [M+K]+ [M+NH4]+ [M+Na]+
DG 16:0_20:5	-3.776284533	-4.419800433	-4.0477	-4.5633	-3.2695	-4.0915	-5.4386	-3.1947	-3.983	-3.7048	-3.246	-3.7607244	-5.0165	DG	614.4891	11.695005	[M*]+ [M+H]+ [M+K]+ [M+NH4]+ [M+Na]+
DG 16:0_20:4	-2.392239233	-2.942479467	-2.7885	-3.2309	-2.0982	-2.7014	-3.1758	-1.824	-2.9352	-2.7059	-2.221	-2.3514417	-3.6797	DG	616.5045	12.894996	[M*]+ [M+H]+ [M+K]+ [M+NH4]+ [M+Na]+
DG 18:1_18:2	-4.0133571	-4.666477833	-2.0263	-3.7676	-3.7362	-2.2939	-2.7274	-2.7115	-2.4791	-4.1593	-1.6786	-3.1455587	-3.0701	DG	618.5211	13.433997	[M*]+ [M+H]+ [M+K]+ [M+NH4]+ [M+Na]+
DG 18:1_18:1	-1.9594434	-2.708012267	0.4889	-2.7462	-1.5462	0.2296	0.3317	-2.3095	-1.0144	-3.2389	0.4197	-2.8373884	-0.9459	DG	620.5372	14.767002	[M*]+ [M+H]+ [M+K]+ [M+NH4]+ [M+Na]+
DG 18:0_18:1	-3.2824059	-3.8672505	-3.1631	-3.9678	-2.9383	-2.616	-3.7663	-3.7036	-4.068	-4.6318	-3.1055	-4.4579208	-4.4862	DG	622.5519	15.393003	[M*]+ [M+H]+ [M+K]+ [M+NH4]+ [M+Na]+
DG 18:1_19:1	-6.905831	-7.1633863	-6.66	-6.9722	-6.0781	-6.0756	-7.502	-6.7816	-7.2235	-8.0081	-6.3744	-7.4482791	-7.9217	DG	634.5534	15.200006	[M*]+ [M+H]+ [M+K]+ [M+NH4]+ [M+Na]+
DG 16:0_22:6	-3.659439733	-3.438472167	-3.7079	-3.6866	-3.2527	-3.6826	-3.4203	-3.3353	-3.5742	-3.3398	-3.2375	-2.992033	-4.0961	DG	640.5051	12.484002	[M*]+ [M+H]+ [M+K]+ [M+NH4]+ [M+Na]+
DG 18:1_20:4	-2.6313254	-3.1474489	-1.7781	-3.2444	-2.0725	-1.903	-1.5794	-1.5382	-2.0648	-2.5985	-1.0384	-1.9127795	-2.5393	DG	642.5206	13.253999	[M*]+ [M+H]+ [M+K]+ [M+NH4]+ [M+Na]+
DG 18:1_20:3	-6.781093267	-7.1894184	-5.2275	-7.1802	-6.0293	-5.6528	-5.5868	-5.8689	-6.0865	-6.3754	-4.5438	-6.0058619	-6.6905	DG	644.536	14.067995	[M*]+ [M+H]+ [M+K]+ [M+NH4]+ [M+Na]+
DG 18:0_20:4	-1.378678633	-1.665469467	-0.6205	-1.6162	-1.1224	-0.6513	-1.0598	-0.5456	-1.5406	-1.4701	-0.5485	-1.0323118	-1.9194	DG	644.5361	14.838996	[M*]+ [M+H]+ [M+K]+ [M+NH4]+ [M+Na]+
DG 18:0_20:3	-4.4980062	-4.808669333	-4.8882	-4.9721	-4.3159	-4.1131	-5.4617	-4.6136	-5.5663	-5.2685	-4.5474	-4.8710562	-6.2475	DG	646.5525	15.187005	[M*]+ [M+H]+ [M+K]+ [M+NH4]+ [M+Na]+
DG 18:1_20:1	-5.570141533	-6.0663649	-4.2109	-5.9236	-5.1771	-4.1418	-5.7913	-6.3487	-6.478	-7.0823	-5.379	-7.7598054	-6.6522	DG	648.5682	15.448	[M*]+ [M+H]+ [M+K]+ [M+NH4]+ [M+Na]+
DG 18:1_22:6	-3.633304567	-4.057781867	-2.7325	-5.2232	-2.7575	-2.4034	-3.6352	-2.6277	-2.9267	-3.3905	-1.568	-2.5507221	-3.9122	DG	666.5204	12.920007	[M*]+ [M+H]+ [M+K]+ [M+NH4]+ [M+Na]+
DG 18:1_22:5	-4.605067667	-5.210342433	-3.1034	-5.0001	-4.0941	-3.3014	-3.7922	-3.3015	-3.9236	-4.6446	-2.7103	-3.8235137	-4.5325	DG	668.5365	13.549995	[M*]+ [M+H]+ [M+K]+ [M+NH4]+ [M+Na]+
DG 18:0_22:6	-4.146798533	-4.782011033	-5.1663	-5.0489	-4.0062	-4.1967	-4.9096	-3.637	-4.8206	-4.7052	-4.0982	-4.6688849	-5.3402	DG	668.5366	14.570005	[M*]+ [M+H]+ [M+K]+ [M+NH4]+ [M+Na]+
DG 18:0_22:5	-4.201464967	-4.599298467	-4.0985	-4.6795	-3.8999	-4.0046	-4.4668	-3.3783	-4.78	-4.5826	-4.15	-3.990462	-5.3704	DG	670.5519	14.978001	[M*]+ [M+H]+ [M+K]+ [M+NH4]+ [M+Na]+
EtherPE 16:1e_20:5	-2.491935733	-3.5324484	-3.3311	-3.4295	-2.7346	-3.1817	-3.646	-3.3609	-4.254	-3.7267	-3.0595	-3.6997903	-4.3719	EtherPE	721.5053	9.329001	[M-H]-
EtherPE 16:1e_20:4	-0.88042958	-1.234980267	-0.3257	-1.1242	-0.7698	-0.9645	-0.4756	-0.794	-1.3558	-1.1464	-0.5514	-1.071867	-1.4895	EtherPE	723.5224	10.346002	[M+HCOO]+ [M-H]-
EtherPE 18:2e_20:4	-1.5195624	-1.769779233	-1.336	-1.6077	-1.3841	-1.8592	-1.5994	-1.4202	-2.079	-1.9182	-1.4674	-1.6695125	-2.1085	EtherPE	749.5424	10.668	[M+CH3COO]+ [M+HCOO]+ [M-H]-
EtherPE 18:2e_22:6	-3.6967659	-4.240508	-3.7695	-4.2984	-3.901	-4.2452	-4.5455	-4.5589	-4.9341	-4.7587	-4.3085	-4.5209914	-5.1534	EtherPE	773.5348	10.369997	[M+CH3COO]+ [M+HCOO]+ [M-H]-
EtherPE 18:1e_22:6	-3.477661733	-4.038945167	-3.7925	-4.3538	-3.561	-3.9836	-5.4044	-3.8918	-4.7777	-4.6379	-4.048	-4.1840178	-5.5724	EtherPE	775.5524	11.924004	[M+CH3COO]+ [M+HCOO]+ [M-H]-
FA 16:1	0.64367167	-0.034706752	1.0996	-0.2635	0.7165	0.6001	0.4547	0.9146	0.1438	-0.2728	1.4368	1.56937917	-0.5523	FA	254.2249	2.921999	[M+CH3COO]+ [M+HCOO]+ [M-H]-
FA 16:0	0.650180183	0.635749183	0.2321	0.609	0.784	0.2014	0.8376	0.8276	0.2407	1.8749	0.4174	0.04023171	0.2306	FA	256.2407	4.2030015	[M+CH3COO]+ [M+HCOO]+ [M-H]-
FA 17:1	-0.918493263	-1.4941724	-1.0678	-1.5143	-0.0368	-1.2721	-1.0889	-0.2339	-1.2998	-1.6733	-0.4277	0.58007813	-2.3995	FA	268.2404	3.3850005	[M+CH3COO]+ [M+HCOO]+ [M-H]-
FA 17:0	-1.5114619	-1.233812947	-1.6014	-2.1704	-1.1431	-3.5089	-0.533	0.0637	-0.8833	-1.9459	-1.2008	2.25154943	-2.5247	FA	270.2562	3.8970015	[M+CH3COO]+ [M+HCOO]+ [M-H]-
FA 18:1	4.561214433	3.957038233	5.2936	3.7998	5.0476	5.1549	5.3578	4.7487	4.8723	3.0653	5.4231	5.15806267	4.2179	FA	282.2566	3.837999	[M+CH3COO]+ [M+HCOO]+ [M-H]-
FA 18:0	1.417666767	1.391419067	1.1144	1.3156	1.4684	1.123	1.4672	1.577	0.9303	2.4651	1.2339	1.0536251	1.0499	FA	284.2722	4.9259987	[M+CH3COO]+ [M+HCOO]+ [M-H]-
FA 19:1	-0.96893628	-1.7455063	-2.0107	-1.5139	-0.2956	-1.5997	-2.3167	-1.3432	-2.2638	-2.4319	-1.4515	0.89507549	-3.5334	FA	296.2717	4.227	[M+CH3COO]+ [M+HCOO]+ [M-H]-
FA 20:3	0.202962877	-0.68658512	-0.9268	-0.8283	0.2411	-0.3025	-0.956	0.3148	-1.15	-0.7901	0.5861	0.38122495	-1.8251	FA	306.2563	3.6239996	[M+CH3COO]+ [M+HCOO]+ [M-H]-
FA 20:1	0.001914978	-0.924474727	0.4728	-0.6396	0.6359	0.5245	-0.4109	-0.5561	-1.4366	-2.7776	-0.2875	1.7075297	-1.985	FA	310.2878	4.605	[M+CH3COO]+ [M+HCOO]+ [M-H]-
FA 20:0	-0.594874693	-1.550662367	-1.4872	-1.6404	-0.4644	-1.6263	-0.1839	-0.2293	-1.4453	-1.7525	-1.026	3.03559937	-2.1088	FA	312.303	5.294002	[M+CH3COO]+ [M+HCOO]+ [M-H]-
FA 22:4	-1.209916467	-2.2266369	-0.6461	-1.5729	-0.9844	-1.5653	-1.056	-0.4178	-2.0893	-2.451	-0.6896	-0.5149396	-2.7951	FA	332.2719	3.967	[M+CH3COO]+ [M+HCOO]+ [M-H]-
FA 22:1	-2.494531	-3.250689833	-2.7222	-3.0722	-2.3066	-2.7465	-2.9862	-2.8697	-3.8388	-3.9152	-3.1344	-2.2592093	-4.0684	FA	338.3189	5.4270015	[M+CH3COO]+ [M+HCOO]+ [M-H]-
FA 22:0	-1.554410933	-2.267029467	-2.5618	-2.4199	-1.2958	-2.2146	-1.3184	-1.0736	-2.247	-2.5213	-1.9132	0.95534388	-2.5914	FA	340.3344	6.4360013	[M-H]-
FAHFA 2:0_16:2	-3.438992233	-3.602172867	-3.7597	-3.9846	-3.202	-3.6487	-3.5117	-2.6967	-3.6297	-4.6884	-3.4377	-2.7833691	-4.7783	FAHFA	310.2122	2.555	[M+CH3COO]+ [M+HCOO]+ [M-H]-
FAHFA 2:0_20:4	-3.998939833	-4.67768	-3.8748	-4.1049	-3.3466	-3.7228	-4.0991	-3.0286	-3.9473	-4.4938	-2.8422	-2.9370811	-4.9852	FAHFA	362.2439	3.2559984	[M+CH3COO]+ [M-H]-
FAHFA 16:0_18:2	-2.032056167	-2.6684602	-2.6675	-3.009	-0.9445	-3.4161	-2.2558	-1.2233	-2.5866	-2.42	-2.2445	-0.3069744	-4.7713	FAHFA	534.463	3.585	[M+CH3COO]+ [M-H]-
FAHFA 18:1_20:3	-2.759767467	-3.651780433	-0.5784	-4.0401	-1.7081	-0.8708	-0.3959	-2.3746	-1.4236	-5.2997	-0.2963	-1.2272008	-2.8218	FAHFA	586.4946	3.437999	[M+CH3COO]+ [M-H]-
FAHFA 18:0_20:2	-1.6979637	-3.0586955	-2.6527	-3.2368	-1.8464	-3.0454	-2.2427	-1.5552	-3.3642	-3.63	-2.4307	-0.4108238	-4.3454	FAHFA	590.5259	4.4510026	[M+CH3COO]+ [M+HCOO]+ [M-H]-
LPC 15:0/0:0	-7.958033233	-8.219439333	-8.2362	-8.0345	-8.0772	-8.3073	-8.0967	-7.9967	-8.0833	-8.1101	-7.9833	-8.124768	-8.0917	LPC	481.3158	2.8150008	[M*]+ [M+H]+ [M+K]+ [M+NH4]+ [M+Na]+
LPC 16:1/0:0	-8.101488667	-9.290021	-9.1674	-9.0062	-8.5626	-8.9156	-7.7017	-8.336	-9.1047	-9.9327	-7.7521	-8.3695233	-9.0243	LPC	493.316	2.6370008	[M*]+ [M+H]+ [M+K]+ [M+NH4]+ [M+Na]+
LPC 16:0/0:0	-2.7792962	-4.1555277	-6.4331	-4.1376	-3.6492	-4.9991	-5.327	-4.1663	-5.6557	-5.7518	-4.4441	-4.3326765	-6.094	LPC	495.3319	3.175001	[M*]+ [M+H]+ [M+K]+ [M+NH4]+ [M+Na]+
LPC 18:3/0:0	-4.7439142	-6.333456667	-8.679	-6.3302	-5.7611	-7.157	-7.5937	-6.2345	-7.8668	-7.9995	-6.5027	-6.40327	-8.3623	LPC	517.3137	3.275001	[M*]+ [M+H]+ [M+K]+ [M+NH4]+ [M+Na]+
LPC 0:0/18:2	-9.928522	-11.008565	-11.346	-9.836	-9.4835	-9.8872	-12.05	-9.9165	-11.445	-12.291	-8.8771	-9.4964757	-11.909	LPC	519.3314	2.7600005	[M*]+ [M+H]+ [M+K]+ [M+NH4]+ [M+Na]+
LPC 18:1/0:0	-5.018600433	-6.0795569	-5.0116	-5.2675	-5.3729	-4.8647	-4.1181	-5.3345	-5.8368	-6.9928	-3.8184	-5.4565646	-5.7104	LPC	521.3474	3.4879994	[M*]+ [M+H]+ [M+K]+ [M+NH4]+ [M+Na]+
LPC 18:0/0:0	-3.892318733	-4.9854788	-5.7336	-4.6218	-4.4512	-4.9111	-4.2024	-4.1066	-5.2707	-5.6771	-3.7379	-4.1727199	-5.3654	LPC	523.3631	4.0820003	[M*]+ [M+H]+ [M+K]+ [M+NH4]+ [M+Na]+
LPC 0:0/20:4	-9.646931333	-10.46902967	-9.6692	-10.169	-9.0704	-9.4543	-9.4113	-8.893	-10.03	-10.549	-8.7069	-9.5219652	-10.226	LPC	543.3316	2.7709985	[M*]+ [M+H]+ [M+K]+ [M+NH4]+ [M+Na]+
LPC 0:0/26:0	-10.19417967	-11.09257167	-11.519	-10.791	-10.545	-10.847	-12.07	-10.289	-11.679	-11.879	-11.213	-10.515894	-11.754	LPC	635.4877	6.574002	[M*]+ [M+H]+ [M+K]+ [M+NH4]+ [M+Na]+
LPE 16:0	-2.0062224	-3.029271433	-5.6672	-3.1911	-2.7338	-3.836	-5.3264	-3.8339	-5.1998	-5.0283	-3.8321	-4.345047	-5.7094	LPE	453.2846	3.356001	[M*]+ [M+H]+ [M+K]+ [M+NH4]+ [M+Na]+
LPE 18:0	0.905792227	-0.088486355	-0.8279	0.2449	0.5285	0.0875	-0.4402	0.0289	-1.1369	-0.68	0.6192	-0.0744362	-1.6418	LPE	481.3179	4.1490016	[M+CH3COO]+ [M+HCOO]+ [M-H]-
MGDG 13:1_18:1	-3.163759233	-4.1661182	-4.4581	-4.0786	-2.5186	-3.3088	-2.9007	-0.9248	-2.1421	-2.8646	-3.2619	-2.6438796	-2.721	MGDG	712.5068	7.195004	[M+CH3COO]+ [M+HCOO]+ [M-H]-
PC 29:0	-4.973501867	-5.953510867	-7.577	-5.8728	-5.389	-6.7075	-9.0429	-6.5926	-8.7952	-7.6298	-7.475	-7.0536951	-9.1499	PC	691.5136	7.705997	[M*]+ [M+H]+ [M+K]+ [M+NH4]+ [M+Na]+
PC 30:1	-2.5212307	-3.5219059	-4.2896	-3.6676	-2.7905	-4.0347	-5.8131	-4.2293	-6.0764	-5.1094	-4.5277	-4.8953629	-6.2282	PC	703.5141	7.4140005	[M*]+ [M+H]+ [M+K]+ [M+NH4]+ [M+Na]+
PC 14:0_16:0	-0.524428687	-1.2546393	-2.9966	-1.2432	-0.7726	-1.651	-4.5534	-2.3065	-4.1803	-3.1878	-3.1951	-2.7660351	-4.243	PC	705.5323	8.446003	[M+CH3COO]+ [M+HCOO]+ [M-H]-
PC 31:4	-3.618881233	-4.642981767	-4.2646	-4.2763	-3.6707	-4.6929	-4.1656	-3.776	-5.7341	-5.2506	-4.2371	-4.3499718	-5.2931	PC	711.4866	7.851001	[M*]+ [M+H]+ [M+K]+ [M+NH4]+ [M+Na]+
PC 31:2	-6.834901867	-6.878185967	-7.916	-7.0142	-6.8589	-7.6592	-8.6425	-6.9598	-8.2841	-7.7629	-7.5231	-7.2473321	-8.1233	PC	715.5187	9.376005	[M*]+ [M+H]+ [M+K]+ [M+NH4]+ [M+Na]+
PC 31:1	-4.527287167	-5.812531733	-5.6027	-6.0748	-5.3185	-6.0693	-6.1747	-5.7673	-6.4565	-5.7926	-5.2923	-5.2989463	-6.6454	PC	717.5295	8.135	[M*]+ [M+H]+ [M+K]+ [M+NH4]+ [M+Na]+
PC 31:0	-5.508591933	-6.242148033	-7.5762	-6.2152	-5.7938	-6.8557	-7.7467	-6.272	-7.5949	-7.1638	-7.1482	-6.8660908	-8.0279	PC	719.545	9.002004	[M*]+ [M+H]+ [M+K]+ [M+NH4]+ [M+Na]+
PC 32:3	-3.3457966	-3.7317797	-5.2051	-3.6837	-3.488	-4.0477	-6.5804	-4.5472	-6.3207	-5.3438	-5.2902	-4.943065	-6.5341	PC	727.5136	8.441	[M*]+ [M+H]+ [M+K]+ [M+NH4]+ [M+Na]+
PC 32:2	-3.041985833	-4.118274667	-3.5264	-3.572	-3.0362	-3.9377	-3.5194	-3.1552	-5.1092	-4.6046	-3.4568	-3.793045	-4.6753	PC	729.53	7.8080015	[M*]+ [M+H]+ [M+K]+ [M+NH4]+ [M+Na]+
PC 16:0_16:1	-0.00572777	-0.291955313	-1.5449	-0.6358	0.2342	-1.1301	-1.563	-0.8066	-1.7268	-1.5577	-1.0526	-1.7280464	-1.8442	PC	731.5461	8.921998	[M*]+ [M+H]+ [M+K]+ [M+Na]+
PC 16:0_16:0	0.155184427	-0.329565053	-1.2267	-0.3074	0.0528	-0.4146	-1.2389	-0.3652	-1.4443	-1.1298	-0.8041	-0.4888369	-1.3652	PC	733.5638	10.131998	[M+CH3COO]+ [M+HCOO]+ [M-H]-
PC 33:5	-4.8104064	-5.6624749	-7.3621	-6.0836	-5.0701	-6.7713	-8.4892	-6.069	-8.2948	-6.5155	-7.5937	-6.3043327	-8.9814	PC	737.4992	11.513	[M*]+ [M+H]+ [M+K]+ [M+NH4]+ [M+Na]+
PC 33:2	-6.444989567	-6.6010303	-6.6281	-6.0043	-6.196	-6.853	-7.1777	-5.3314	-7.4876	-6.8106	-6.2511	-5.6736265	-8.252	PC	743.5449	11.4800005	[M*]+ [M+H]+ [M+K]+ [M+NH4]+ [M+Na]+
PC 33:1	-2.8641103	-3.5091133	-4.2952	-3.3912	-3.063	-4.1644	-4.6687	-3.2155	-4.8085	-4.2676	-3.9049	-3.5668882	-5.2812	PC	745.5609	12.673003	[M*]+ [M+H]+ [M+K]+ [M+NH4]+ [M+Na]+
PC 33:0	-4.578703567	-5.147235767	-6.0391	-4.6897	-5.1254	-5.9317	-5.6185	-4.7589	-6.8777	-6.2249	-5.1655	-4.781292	-6.1859	PC	747.5752	11.091002	[M*]+ [M+H]+ [M+NH4]+ [M+Na]+
PC 34:5	-5.671017267	-6.721566467	-8.4114	-6.8541	-5.7736	-7.3754	-10.146	-6.7747	-9.1598	-7.8265	-8.2311	-7.4124787	-9.5977	PC	751.5143	6.9029994	[M*]+ [M+H]+ [M+K]+ [M+NH4]+ [M+Na]+
PC 16:0_18:5	-9.009981333	-9.049995	-9.0279	-9.0908	-8.9736	-9.2548	-9.9985	-7.3123	-9.2282	-8.3571	-8.2379	-7.6499989	-10.048	PC	751.5164	8.410003	[M*]+ [M+H]+ [M+K]+ [M+NH4]+ [M+Na]+
PC 34:4	-4.0346527	-5.165941833	-5.2508	-5.0312	-4.1248	-5.4545	-6.4312	-4.2913	-6.3645	-5.4869	-4.9971	-4.9332433	-6.5338	PC	753.529	7.721999	[M*]+ [M+H]+ [M+K]+ [M+NH4]+ [M+Na]+
PC 16:1_18:2	-6.1174087	-6.8188895	-6.7648	-5.7144	-5.8778	-7.4152	-7.0078	-5.9118	-7.0257	-6.4734	-7.1904	-5.9496783	-6.8427	PC	755.5452	8.138	[M*]+ [M+H]+ [M+K]+ [M+NH4]+ [M+Na]+
PC 16:1_18:1	-1.068601613	-1.780785233	-0.854	-0.8831	-1.0903	-1.2156	-1.1236	-1.6439	-2.2854	-2.4389	-1.1379	-1.5981757	-2.1058	PC	757.5616	9.3150015	[M*]+ [M+H]+ [M+K]+ [M+NH4]+ [M+Na]+
PC 16:0_18:1	1.180470133	0.376780183	0.2075	0.5669	1.0316	0.5747	0.1147	0.6437	-0.1467	-0.04	0.4335	0.24639003	-0.2352	PC	759.5798	10.493003	[M+CH3COO]+ [M+HCOO]+ [M-H]-
PC 35:7	-4.808724733	-4.9811007	-4.075	-4.611	-4.9148	-4.6706	-4.4784	-5.1378	-5.2733	-5.7464	-4.5805	-5.3803895	-5.1834	PC	761.4971	9.347995	[M*]+ [M+H]+ [M+K]+ [M+NH4]+ [M+Na]+
PC 16:0_18:0	-1.0000337	-1.6464939	-2.2761	-1.1204	-1.1687	-1.6574	-1.7114	-0.8525	-2.4975	-2.1876	-1.3223	-1.0088781	-2.1409	PC	761.5947	12.075996	[M+CH3COO]+ [M+HCOO]+ [M-H]-
PC 17:2_18:3	-8.674787	-9.562651333	-10.769	-9.9319	-9.0976	-10.916	-11.237	-8.8855	-11.554	-9.5259	-9.0391	-8.984324	-11.974	PC	765.5292	7.554996	[M*]+ [M+H]+ [M+K]+ [M+NH4]+ [M+Na]+
PC 19:1_16:3	-7.893075333	-8.205411333	-7.765	-7.7808	-7.4319	-8.1338	-8.2229	-6.064	-7.7311	-6.9354	-6.8845	-6.3248361	-8.2119	PC	767.5431	8.4190035	[M*]+ [M+H]+ [M+K]+ [M+NH4]+ [M+Na]+
PC 16:1_19:2	-8.138139667	-8.830266833	-10.503	-7.0926	-7.9076	-8.4725	-9.9958	-7.1321	-9.2136	-8.6433	-9.4253	-7.317388	-9.1245	PC	769.5589	8.838998	[M*]+ [M+H]+ [M+K]+ [M+NH4]+ [M+Na]+
PC 35:3	-2.024775767	-2.841888433	-1.1548	-2.657	-2.4977	-1.5203	-1.3358	-2.7591	-2.9887	-3.9369	-1.5722	-3.3380299	-2.3642	PC	769.5689	10.885003	[M*]+ [M+H]+ [M+K]+ [M+NH4]+ [M+Na]+
PC 35:2	-4.3170744	-4.943442	-4.0597	-4.3558	-4.1547	-4.7253	-4.7645	-4.4054	-5.2992	-5.397	-4.1952	-4.8020675	-5.5576	PC	771.5766	10.001005	[M*]+ [M+H]+ [M+K]+ [M+NH4]+ [M+Na]+
PC 35:1	-2.969044333	-3.9980068	-4.5744	-3.6478	-3.3444	-4.1629	-4.9464	-3.4932	-5.5784	-4.8251	-4.3078	-4.1381327	-5.4405	PC	773.5922	11.498995	[M*]+ [M+H]+ [M+K]+ [M+NH4]+ [M+Na]+
PC 36:7	-6.4122835	-7.6376272	-8.993	-7.9025	-6.6575	-8.7323	-10.507	-6.7674	-9.3867	-7.9422	-7.9668	-8.0479367	-9.9187	PC	775.5142	7.705997	[M*]+ [M+H]+ [M+K]+ [M+NH4]+ [M+Na]+
PC 35:0	-7.081329367	-7.9294599	-9.4961	-7.6807	-7.3325	-8.5654	-8.4701	-7.1975	-9.0521	-8.9008	-8.2045	-7.5851682	-9.063	PC	775.6061	12.744997	[M*]+ [M+H]+ [M+NH4]+ [M+Na]+
PC 36:6	-8.950028667	-10.35005	-11.325	-8.7208	-9.3776	-10.394	-9.7277	-8.8441	-10.971	-11.376	-11.129	-9.8735	-9.3305	PC	777.5272	8.1170025	[M*]+ [M+H]+ [M+K]+ [M+NH4]+ [M+Na]+
PC 16:0_20:5	-4.4993146	-4.636970467	-3.543	-4.2147	-4.6407	-4.573	-3.9669	-5.262	-4.9144	-5.2926	-4.2785	-5.0483983	-4.7379	PC	779.5435	9.343996	[M*]+ [M+H]+ [M+K]+ [M+NH4]+ [M+Na]+
PC 36:5	-1.569529833	-2.2552433	-4.2995	-2.702	-1.6723	-2.9755	-4.685	-2.2411	-3.8495	-2.7403	-3.1217	-2.6902345	-4.5973	PC	779.546	8.291995	[M*]+ [M+H]+ [M+K]+ [M+NH4]+ [M+Na]+
PC 16:0_20:4	-0.937205	-1.5684363	-2.8524	-1.8576	-1.0883	-2.0233	-2.6923	-0.8302	-2.145	-1.4491	-1.8265	-1.2116559	-2.7262	PC	781.5612	9.222995	[M*]+ [M+H]+ [M+K]+ [M+NH4]+ [M+Na]+
PC 36:3	-2.7435551	-4.031998933	-4.9661	-3.6219	-2.8818	-4.2552	-5.1737	-3.3267	-5.1006	-4.6391	-5.2943	-3.8696957	-6.2469	PC	783.577	9.898996	[M*]+ [M+H]+ [M+K]+ [M+NH4]+ [M+Na]+
PC 18:1_18:2	-3.543910333	-4.165362	-3.3477	-3.0986	-3.3594	-3.1909	-3.9121	-3.2105	-4.3557	-5.0358	-3.7347	-3.6976871	-4.3043	PC	783.5789	9.691002	[M+CH3COO]+ [M+HCOO]+ [M-H]-
PC 18:1_18:1	-0.661539083	-1.500562067	0.3575	-1.271	-0.7852	-0.1775	0.1635	-1.3964	-1.346	-2.4929	-0.1449	-1.9617418	-0.71	PC	785.5929	10.885995	[M+CH3COO]+ [M+HCOO]+ [M-H]-
PC 18:0_18:2	-2.174745567	-2.8782959	-3.2863	-2.9222	-2.3278	-2.7658	-2.9299	-1.9832	-3.3875	-3.1618	-2.7511	-2.5669549	-3.3513	PC	785.5948	11.144997	[M+CH3COO]+ [M+HCOO]+ [M-H]-
PC 18:0_18:1	-0.21555964	-1.014160167	-1.1586	-1.1164	-0.3411	-0.7272	-1.1017	-0.652	-1.6643	-1.6015	-0.8355	-1.2206535	-1.7093	PC	787.6109	12.447999	[M+CH3COO]+ [M+HCOO]+ [M-H]-
PC 36:0	-6.1525581	-6.909819233	-7.4931	-6.6493	-6.6929	-7.3175	-7.0063	-6.0334	-7.8641	-7.3543	-6.6131	-6.1123428	-7.404	PC	789.623	14.125	[M*]+ [M+H]+ [M+K]+ [M+NH4]+ [M+Na]+
PC 37:6	-7.524574133	-8.95523	-9.7238	-8.3924	-7.4117	-9.3158	-10.034	-7.101	-9.9671	-8.3242	-8.9567	-7.6182118	-10.361	PC	791.5449	7.8589993	[M*]+ [M+H]+ [M+K]+ [M+NH4]+ [M+Na]+
PC 19:1_18:3	-5.8833549	-6.490598	-6.2952	-6.4464	-5.871	-6.7627	-6.2569	-5.3449	-6.6843	-6.1433	-5.6237	-5.5676573	-7.0478	PC	795.5745	10.096001	[M*]+ [M+H]+ [M+K]+ [M+NH4]+ [M+Na]+

PC 37:2	-5.7106462	-6.7493566	-6.8071	-6.2246	-5.8391	-6.7588	-7.4684	-6.4236	-7.7841	-8.3161	-6.9344	-6.9678053	-8.0573	PC	799.6071	11.802001	[M*]+ [M+H]+ [M+K]+ [M+NH4]+ [M+Na]+
PC 38:8	-4.692733833	-4.9624368	-6.8315	-5.4251	-4.718	-5.7297	-7.2388	-5.2526	-6.4834	-5.3649	-5.8576	-5.5907727	-7.2242	PC	801.5283	8.303997	[M*]+ [M+H]+ [M+K]+ [M+NH4]+ [M+Na]+
PC 37:1	-6.796261433	-7.590304767	-8.8506	-7.5239	-6.8106	-8.0516	-8.1947	-7.3218	-8.7605	-8.5245	-8.0696	-7.8737386	-9.4017	PC	801.6229	13.454003	[M*]+ [M+H]+ [M+NH4]+ [M+Na]+
PC 38:7	-5.640144367	-6.905060433	-6.7297	-6.5656	-5.7406	-6.8253	-7.0241	-5.3166	-7.3534	-6.9903	-5.9963	-6.0589491	-7.3548	PC	803.5446	7.8699975	[M*]+ [M+H]+ [M+K]+ [M+NH4]+ [M+Na]+
PC 38:6	-2.697914733	-3.387147933	-5.8685	-3.6958	-2.9719	-4.1008	-5.986	-3.2219	-4.841	-4.4803	-4.235	-3.6470992	-5.3905	PC	805.5609	9.010001	[M*]+ [M+H]+ [M+K]+ [M+NH4]+ [M+Na]+
PC 38:5	-3.152729033	-3.670880667	-4.2599	-4.2569	-3.0031	-3.5862	-4.2218	-3.1093	-4.2759	-3.4764	-3.0311	-3.4913705	-4.7808	PC	807.5768	9.967003	[M*]+ [M+H]+ [M+K]+ [M+NH4]+ [M+Na]+
PC 38:4	-3.0559584	-3.9963754	-4.2337	-4.2327	-3.3759	-3.7491	-4.3019	-3.8069	-5.0514	-5.0283	-3.9531	-4.6161582	-5.0755	PC	809.5907	12.476996	[M*]+ [M+H]+ [M+K]+ [M+NH4]+ [M+Na]+
PC 38:3	-4.8531837	-5.968172633	-7.2157	-6.0367	-5.1183	-6.3137	-6.7162	-5.1269	-6.3816	-5.6569	-5.9699	-5.1595389	-7.2317	PC	811.6077	11.820005	[M*]+ [M+H]+ [M+K]+ [M+NH4]+ [M+Na]+
PC 38:2	-4.657076467	-6.109438033	-4.3893	-5.7185	-5.5489	-5.0328	-5.7076	-5.9243	-7.0031	-7.9127	-6.1808	-7.0235869	-7.2542	PC	813.6228	12.756004	[M*]+ [M+H]+ [M+K]+ [M+NH4]+ [M+Na]+
PC 38:1	-6.078606367	-7.706855867	-7.6631	-7.0193	-6.1947	-6.8754	-8.0364	-6.7519	-8.2383	-8.0701	-7.8288	-7.5858046	-8.4071	PC	815.6381	14.347996	[M*]+ [M+H]+ [M+K]+ [M+NH4]+ [M+Na]+
PC 39:7	-7.5236957	-8.641609	-7.1125	-7.6907	-7.6756	-7.8759	-8.1152	-7.7865	-9.3072	-9.2084	-8.4428	-8.393841	-9.5119	PC	817.5591	12.727998	[M*]+ [M+H]+ [M+K]+ [M+NH4]+ [M+Na]+
PC 39:5	-9.287883167	-10.70663567	-10.129	-9.9678	-9.449	-10.849	-11.931	-10.419	-12.156	-11.258	-10.77	-11.642966	-11.529	PC	821.59	11.812004	[M*]+ [M+H]+ [M+K]+ [M+NH4]+ [M+Na]+
PC 40:8	-6.0866032	-6.498014467	-6.6203	-7.0243	-6.0717	-6.5869	-6.676	-5.9261	-7.0974	-6.3244	-5.7348	-6.2720508	-7.2638	PC	829.5586	9.971995	[M*]+ [M+H]+ [M+K]+ [M+NH4]+ [M+Na]+
PC 40:7	-5.3880405	-5.911922367	-4.5358	-5.5763	-5.3307	-4.8163	-4.8219	-5.1849	-5.4888	-5.8569	-4.2823	-5.4347019	-5.3301	PC	831.5758	9.335	[M*]+ [M+H]+ [M+K]+ [M+NH4]+ [M+Na]+
PC 40:6	-4.7683512	-5.458351767	-6.7729	-5.5539	-4.8912	-5.686	-6.4775	-4.1823	-5.6574	-5.2417	-5.166	-4.5675214	-6.1919	PC	833.5924	10.773995	[M*]+ [M+H]+ [M+K]+ [M+NH4]+ [M+Na]+
PC 40:5	-5.621662233	-5.967948933	-6.1283	-5.4469	-5.8341	-6.1889	-6.1554	-4.884	-6.2528	-6.0882	-5.6971	-5.2115771	-6.6522	PC	835.6051	10.791994	[M*]+ [M+H]+ [M+K]+ [M+NH4]+ [M+Na]+
PC 40:4	-9.708627667	-9.952198667	-11.064	-10.082	-9.1438	-10.206	-11.368	-10.284	-11.413	-11.12	-10.851	-11.092862	-11.387	PC	837.6216	14.424005	[M*]+ [M+H]+ [M+NH4]+
PC 40:1	-9.383152	-10.349262	-11.022	-9.4662	-9.7513	-10.324	-10.262	-9.9706	-10.851	-11.13	-10.449	-10.037007	-11.483	PC	843.6695	15.471992	[M*]+ [M+H]+ [M+K]+ [M+NH4]+ [M+Na]+
PC 20:5_22:6	-12.44724467	-12.12377833	-11.587	-11.309	-11.966	-11.993	-10.522	-10.053	-11.021	-10.493	-10.962	-11.102074	-10.679	PC	851.5415	8.313997	[M*]+ [M+H]+ [M+K]+ [M+NH4]+ [M+Na]+
PC 42:10	-8.985815	-9.819631	-10.758	-9.5227	-8.8368	-9.9637	-7.7616	-6.9739	-8.7821	-8.9044	-8.9797	-8.6323293	-8.6224	PC	853.5596	8.075001	[M*]+ [M+H]+ [M+K]+ [M+NH4]+ [M+Na]+
PC 42:9	-9.470298	-10.50584567	-9.8775	-9.529	-9.2473	-10.503	-8.2584	-7.5894	-9.5128	-8.9314	-9.0813	-8.3756657	-9.0796	PC	855.5756	8.561003	[M*]+ [M+H]+ [M+K]+ [M+NH4]+ [M+Na]+
PC 42:8	-8.018766233	-9.239509667	-8.2084	-8.3994	-8.5112	-8.6636	-8.2683	-7.6398	-8.7517	-8.3167	-8.1002	-8.0695197	-9.1812	PC	857.5895	11.376005	[M*]+ [M+H]+ [M+K]+ [M+NH4]+ [M+Na]+
PC 42:7	-9.522387	-10.55508467	-10.282	-9.171	-9.8683	-10.365	-10.997	-9.6109	-10.89	-10.776	-10.351	-9.8757047	-11.091	PC	859.6023	10.9920025	[M*]+ [M+H]+ [M+K]+ [M+Na]+
PC 42:6	-9.128386333	-9.797175667	-8.3425	-8.1658	-9.6674	-10.155	-9.2532	-9.6197	-11.025	-11.58	-10.028	-10.376316	-11.474	PC	861.6208	11.342004	[M*]+ [M+H]+ [M+K]+ [M+Na]+
PC 42:5	-8.627882333	-9.258225333	-9.1496	-8.0456	-8.6149	-10.07	-9.5647	-8.0703	-10.257	-9.9071	-9.5339	-9.1705043	-10.433	PC	863.6379	12.932005	[M*]+ [M+H]+ [M+K]+ [M+NH4]+ [M+Na]+
PC 22:6_22:6	-9.728112	-11.21340633	-11.083	-10.351	-9.5623	-11.128	-10.475	-8.6002	-10.949	-10.149	-10.122	-9.7671173	-10.442	PC	877.561	7.881	[M*]+ [M+H]+ [M+K]+ [M+NH4]+ [M+Na]+
PE 32:1	0.796294537	-0.184661863	-1.6005	-0.7896	-0.1222	-0.6754	-2.1173	-1.4167	-2.2065	-1.3642	-1.906	-1.8024323	-2.1353	PE	689.5005	9.049001	[M*]+ [M+H]+ [M+K]+ [M+NH4]+ [M+Na]+
PE 32:0	-1.698307033	-2.148609767	-3.7142	-2.3468	-2.1055	-2.688	-3.6361	-2.5226	-3.7913	-2.7517	-3.3446	-2.8034064	-3.5788	PE	691.5136	10.362	[M*]+ [M+H]+ [M+K]+ [M+Na]+
PE 16:0_18:1	2.5925115	1.275574967	0.829	1.9003	1.912	1.546	0.1268	1.0029	0.0714	0.5432	0.4573	0.74096999	0.0138	PE	717.5313	10.759002	[M+H]+ [M+K]+ [M+NH4]+ [M+Na]+
PE 34:0	-0.672688147	-1.3634796	-2.0285	-1.0149	-1.1678	-1.2747	-1.8509	-0.7294	-2.1706	-1.454	-1.1972	-1.1063595	-1.9017	PE	719.5448	12.278998	[M*]+ [M+H]+ [M+K]+ [M+NH4]+ [M+Na]+
PE 17:1_18:1	-1.590422967	-2.028258633	-1.4229	-1.2992	-1.4045	-1.8882	-2.6392	-1.7435	-2.7612	-2.4903	-1.9613	-2.0115547	-2.9832	PE	729.5312	10.283998	[M+CH3COO]+ [M+HCOO]+ [M-H]-
PE 18:0_17:1	-0.703805923	-1.3310534	-0.9191	-0.9892	-0.7075	-1.2316	-2.1056	-1.3928	-2.6625	-2.4529	-1.5923	-1.9827512	-2.7265	PE	731.5466	11.72	[M+CH3COO]+ [M+HCOO]+ [M-H]-
PE 16:0_20:5	2.0952606	1.275951367	-1.6979	0.4959	1.9063	0.1801	-2.7705	0.2248	-1.5928	-0.1277	-0.8826	-0.3176066	-2.3295	PE	737.5011	8.5180025	[M+CH3COO]+ [M+HCOO]+ [M-H]-
PE 16:0_20:4	3.412125267	2.522645933	-0.2825	1.7908	3.2827	1.6398	-0.7714	2.2387	0.2173	1.5055	0.8829	1.65838493	-0.4777	PE	739.517	9.506998	[M+CH3COO]+ [M+HCOO]+ [M-H]-
PE 18:1_18:2	0.579761493	-0.094572066	0.0488	0.5984	0.9765	0.624	-0.6906	0.3284	-0.7835	-1.1134	-0.1281	-0.1486613	-1.1208	PE	741.5313	9.916005	[M+CH3COO]+ [M+HCOO]+ [M-H]-
PE 16:0_20:3	-1.8078429	-2.497966167	-4.1335	-3.0056	-2.5577	-3.2628	-5.0686	-3.0271	-4.3655	-3.0406	-4.5704	-3.2454367	-4.4889	PE	741.5318	10.696006	[M+CH3COO]+ [M+HCOO]+ [M-H]-
PE 18:1_18:1	2.4450785	1.21210608	3.5181	2.2285	1.9209	3.0828	2.4667	0.4332	0.8614	0.5194	2.527	0.52440834	1.7736	PE	743.5468	8.852006	[M*]+ [M+H]+ [M+K]+ [M+NH4]+ [M+Na]+
PE 18:0_18:2	2.2658138	1.436136233	1.1734	2	1.2295	1.7517	0.6056	-0.2228	0.369	0.2347	2.6734	0.26736132	1.3514	PE	743.5469	8.165002	[M*]+ [M+H]+ [M+K]+ [M+NH4]+ [M+Na]+
PE 18:0_18:1	2.611607267	1.9297339	2.7796	1.9033	2.5774	2.5038	1.719	1.8142	0.9364	0.9287	2.0673	1.18075437	0.9728	PE	745.5638	9.661004	[M+CH3COO]+ [M+HCOO]+ [M-H]-
PE 17:0_20:5	-1.520302467	-1.979280467	-2.318	-1.9187	-1.3039	-2.2038	-2.9201	-2.2514	-3.0053	-2.5693	-2.1568	-2.5232309	-3.1512	PE	751.5162	9.369997	[M+CH3COO]+ [M+HCOO]+ [M-H]-
PE 17:0_20:4	-0.514451343	-0.931523643	-0.988	-0.807	-0.5353	-1.2624	-1.6599	-0.5427	-1.6947	-1.113	-0.8484	-0.5372219	-2.2569	PE	753.5321	10.349	[M+CH3COO]+ [M+HCOO]+ [M-H]-
PE 18:1_20:5	0.51398405	0.13869031	0.6394	0.2797	0.0506	1.0222	-1.4329	-2.3807	-1.92	-0.1786	-0.4918	-1.5220807	-2.0501	PE	763.5149	8.826997	[M*]+ [M+H]+ [M+K]+ [M+NH4]+ [M+Na]+
PE 38:6	5.522274033	4.6245461	3.6529	4.7238	4.7886	4.4006	3.2645	4.9084	3.6576	4.8806	4.3056	4.79155537	3.058	PE	763.5176	9.226998	[M*]+ [M+H]+ [M+K]+ [M+NH4]+ [M+Na]+
PE 38:5	3.125025433	1.964930867	0.5637	2.1193	1.7304	2.0866	0.6103	1.6688	0.0017	1.9102	1.9627	1.8070704	0.1855	PE	765.5302	10.243003	[M*]+ [M+H]+ [M+K]+ [M+NH4]+ [M+Na]+
PE 18:1_20:4	3.2662233	2.563053767	2.5533	2.4908	3.2756	2.8189	1.5426	2.6417	1.2858	1.844	2.6353	2.34215737	1.098	PE	765.5328	9.817995	[M+CH3COO]+ [M+HCOO]+ [M-H]-
PE 18:0_20:4	4.3851903	3.738690033	3.3293	3.6255	4.4381	3.7483	2.7429	4.0693	2.7176	3.3327	3.5749	3.7794094	2.3169	PE	767.5488	11.299996	[M+HCOO]+ [M-H]-
PE 38:3	-0.397587433	-1.502598467	-1.1686	-1.2682	-0.8097	-1.283	-2.4758	-2.0903	-2.8009	-1.2335	-1.3264	-1.9749782	-2.9736	PE	769.5616	12.019005	[M*]+ [M+H]+ [M+K]+ [M+NH4]+ [M+Na]+
PE 18:0_20:3	-0.001709618	-0.6239376	-1.129	-1.1266	-0.0505	-0.6101	-1.6034	-0.837	-1.8282	-1.0339	-1.1794	-1.0198167	-2.1467	PE	769.5633	11.985994	[M+CH3COO]+ [M+HCOO]+ [M-H]-
PE 19:1_20:4	-3.085265167	-3.755396533	-3.6335	-3.0903	-2.9037	-4.0938	-4.9724	-3.0638	-5.0172	-3.6503	-3.7632	-3.0622508	-5.4001	PE	779.5476	10.651	[M+CH3COO]+ [M+HCOO]+ [M-H]-
PE 18:1_22:6	1.3111477	0.49303627	1.4778	0.5366	1.3859	1.2875	-0.1747	0.8111	-0.3743	-0.0885	1.3535	0.45272764	-0.1667	PE	789.5328	9.579997	[M+CH3COO]+ [M-H]-
PE 18:0_22:6	2.373620333	1.756642	0.4841	1.5822	2.3037	1.4437	-0.4665	1.4309	0.1401	0.9087	1.0311	1.31536293	-0.0791	PE	791.5481	11.03	[M+CH3COO]+ [M+HCOO]+ [M-H]-
PE 40:5	1.641064967	0.956031143	0.7305	1.309	1.1833	1.277	-0.0548	0.7907	-0.5695	0.5384	0.728	0.98847647	-1.0191	PE	793.5613	11.566995	[M*]+ [M+H]+ [M+K]+ [M+NH4]+ [M+Na]+
PE 40:4	1.705581	0.014455167	2.2199	1.6042	0.738	1.4604	0.5413	0.4563	-0.6689	-0.453	0.4	0.21442986	-0.8637	PE	795.5762	12.726996	[M*]+ [M+H]+ [M+K]+ [M+NH4]+ [M+Na]+
PE 20:0_20:4	-2.9485032	-3.7315098	-3.6892	-4.3077	-2.7583	-4.5208	-5.2255	-3.5712	-5.5173	-4.4361	-4.5295	-4.2914905	-5.9374	PE	795.5788	13.209998	[M+CH3COO]+ [M+HCOO]+ [M-H]-
PE 18:0_22:4	-0.37496185	-0.961945863	0.1757	-0.003	-0.1891	-0.7943	-0.7393	0.0795	-1.5295	-1.1945	-0.6485	-0.1953952	-1.9899	PE	795.579	12.678993	[M+CH3COO]+ [M+HCOO]+ [M-H]-
PE 18:0_22:3	-2.766907367	-3.593838033	-3.5867	-3.3005	-2.7341	-3.4815	-3.9351	-2.7899	-4.1382	-3.9136	-3.3108	-3.4133396	-4.6761	PE	797.5932	13.936001	[M+CH3COO]+ [M+HCOO]+ [M-H]-
PE 42:5	-1.92261249	-2.982043	-4.0134	-1.8989	-2.4733	-3.1547	-4.8227	-3.1023	-4.5809	-3.3897	-3.5791	-3.5522274	-5.0166	PE	821.5944	13.093996	[M*]+ [M+H]+ [M+K]+ [M+NH4]+ [M+Na]+
PG 16:0_16:0	-6.6616846	-6.9603482	-7.9598	-6.4202	-6.9203	-7.4555	-7.1365	-6.5637	-8.1932	-8.0847	-7.2736	-6.5752461	-8.0382	PG	722.5105	7.5399985	[M+CH3COO]+ [M+HCOO]+ [M-H]-
PG 16:0_18:1	-0.20875295	-0.980041507	-2.2478	-0.8097	-0.3241	-1.1906	-2.035	-0.9372	-2.7758	-2.7391	-1.3063	-0.9073582	-3.1389	PG	748.5272	7.820004	[M+CH3COO]+ [M+HCOO]+ [M-H]-
PG 18:1_18:2	-7.917675667	-7.938094333	-8.0575	-7.2475	-6.6891	-6.0927	-7.9088	-7.615	-8.6157	-9.9276	-8.4305	-8.3131137	-8.8963	PG	772.5247	6.5520015	[M+CH3COO]+ [M+HCOO]+ [M-H]-
PG 18:1_18:1	-4.311640433	-4.470933933	-4.078	-4.0212	-3.9256	-4.173	-3.3866	-3.6852	-4.5112	-5.1138	-3.2937	-3.420337	-4.4263	PG	774.5426	8.079998	[M+CH3COO]+ [M+HCOO]+ [M-H]-
PG 20:4_22:6	-5.873947767	-6.3579082	-5.9488	-6.4584	-5.7825	-6.5855	-7.6238	-5.5829	-7.3215	-6.2211	-5.7966	-5.1603158	-8.3835	PG	842.508	5.7799993	[M+CH3COO]+ [M+HCOO]+ [M-H]-
PI 16:0_20:5	-4.333879467	-4.8439932	-5.9522	-5.8522	-4.1235	-5.7807	-6.3899	-4.034	-5.4344	-5.9463	-6.4192	-6.1705312	-6.7258	PI	856.5123	6.1159983	[M+CH3COO]+ [M+HCOO]+ [M-H]-
PI 16:0_20:4	-0.588905333	-0.984922433	-3.1531	-1.5759	-0.4104	-1.8644	-3.0506	-0.4592	-1.9037	-2.0111	-2.5749	-1.4759134	-2.9585	PI	858.5273	6.6869974	[M+CH3COO]+ [M+HCOO]+ [M-H]-
PI 18:1_18:1	-0.860358533	-1.2074108	0.2485	-1.1166	-0.7247	-0.1425	1.1634	0.3229	-0.266	-2.0556	-0.474	-0.9496523	-0.2755	PI	862.5582	7.796	[M+CH3COO]+ [M+HCOO]+ [M-H]-
PI 17:0_20:4	-3.586609533	-3.7866974	-3.3709	-3.4256	-2.8879	-3.5616	-3.3097	-2.1441	-3.1588	-3.696	-3.3244	-2.9729792	-4.278	PI	872.5433	7.2650013	[M+CH3COO]+ [M+HCOO]+ [M-H]-
PI 18:1_20:4	-0.45658238	-0.7094962	0.1182	-0.2157	-0.0132	-0.1224	0.5258	0.6097	-0.2161	-1.3847	-0.2429	-0.192227	-0.3492	PI	884.5425	6.8999977	[M+CH3COO]+ [M+HCOO]+ [M-H]-
PI 18:0_20:4	3.725740467	3.118250533	3.6233	3.2281	4.2215	3.8595	3.5726	5.3903	3.6871	3.0806	3.9443	4.3440069	2.8901	PI	886.5595	7.8699975	[M+CH3COO]+ [M+HCOO]+ [M-H]-
PI 18:0_20:3	-2.917015067	-2.852082567	-2.4056	-3.4022	-2.1372	-1.7	-2.2889	-0.6875	-1.4385	-2.8587	-2.4937	-2.1502183	-2.5882	PI	888.5737	8.385998	[M+CH3COO]+ [M+HCOO]+ [M-H]-
PI 18:0_22:6	-0.341982523	-0.62826855	-1.9329	-1.2412	-0.1388	-1.0055	-1.9124	-0.0131	-1.0373	-1.6552	-1.5087	-1.2309577	-1.8119	PI	910.5568	7.786999	[M+CH3COO]+ [M+HCOO]+ [M-H]-
PI 18:0_22:5	-1.2114512	-1.2936821	-2.0704	-1.6462	-0.8512	-1.4076	-2.0012	-0.5819	-1.5599	-2.1619	-2.1724	-1.6010513	-2.4053	PI	912.5743	8.085001	[M+CH3COO]+ [M+HCOO]+ [M-H]-
SM d30:1	-8.239311167	-9.203416667	-9.2533	-9.6439	-8.6554	-9.0665	-10.526	-9.5028	-10.735	-10.485	-9.5539	-9.9759718	-11.018	SM	646.5027	5.765	[M*]+ [M+H]+ [M+K]+ [M+NH4]+ [M+Na]+
SM d30:0	-9.007675	-9.798843	-8.133	-9.3888	-9.1413	-9.8023	-10.807	-9.2813	-10.486	-9.4396	-8.6895	-9.6888752	-11.059	SM	648.5185	6.1519966	[M*]+ [M+H]+ [M+K]+ [M+NH4]+ [M+Na]+
SM d31:1	-7.896750867	-8.667762	-8.1709	-8.7863	-8.0599	-8.9966	-9.7697	-8.5569	-9.8812	-9.4117	-8.3461	-8.720264	-9.8542	SM	660.518	6.265	[M*]+ [M+H]+ [M+K]+ [M+Na]+
SM d32:2	-5.819601	-6.88679	-7.5225	-7.3975	-6.3648	-7.0272	-8.0856	-7.1693	-8.3517	-8.3773	-7.8054	-8.010571	-8.6314	SM	672.5185	6.014003	[M*]+ [M+H]+ [M+K]+ [M+NH4]+ [M+Na]+
SM d32:1	-2.1412156	-3.206136067	-3.4963	-3.2411	-2.4906	-3.2004	-4.2564	-2.7068	-4.4833	-4.1734	-3.2853	-3.4517842	-4.4551	SM	674.5351	6.849	[M*]+ [M+H]+ [M+K]+ [M+NH4]+ [M+Na]+
SM d32:0	-3.758221933	-4.689247133	-3.4845	-4.1787	-3.8151	-4.338	-4.0187	-3.1938	-4.8344	-4.6128	-3.3081	-3.8849322	-4.9445	SM	676.5505	7.3520007	[M*]+ [M+H]+ [M+K]+ [M+NH4]+ [M+Na]+
SM d33:1	-2.271787	-3.184321067	-3.1846	-2.9537	-2.5047	-3.3734	-3.7402	-2.7119	-4.1838	-4.0294	-2.8121	-3.2776782	-4.2434	SM	688.5501	7.4900002	[M*]+ [M+H]+ [M+K]+ [M+NH4]+ [M+Na]+
SM d33:0	-4.2717171	-5.063845333	-4.0743	-4.6791	-4.1224	-3.4937	-4.2085	-4.0695	-5.3607	-5.0067	-3.7135	-4.2348373	-5.241	SM	690.5655	8.062	[M*]+ [M+H]+ [M+K]+ [M+NH4]+ [M+Na]+
SM d34:3	-5.454601933	-6.396499667	-5.3542	-6.2336	-5.8767	-6.3632	-6.2505	-5.2122	-6.8848	-6.5085	-5.221	-6.1488558	-6.9104	SM	698.5339	7.346999	[M*]+ [M+H]+ [M+K]+ [M+NH4]+ [M+Na]+
SM d34:2	0.40426572	-0.51217461	-1.4203	-0.6151	0.0183	-0.7817	-1.824	-0.313	-1.7548	-1.658	-1.0146	-1.2291177	-1.8024	SM	700.5512	7.1649976	[M*]+ [M+H]+ [M+K]+ [M+NH4]+ [M+Na]+
SM d34:1	1.304407133	0.643411617	0.6431	0.8103	1.1298	0.8092	0.4424	1.1557	0.1985	0.2154	0.9095	0.77064387	0.2284	SM	702.5694	8.2109995	[M+CH3COO]+ [M+HCOO]+ [M-H]-
SM d34:0	-1.014541	-1.5697231	0.0483	-0.9804	-1.0643	-0.9719	0.2717	-0.067	-0.813	-0.937	0.2133	-0.3425166	-0.6642	SM	704.5845	8.847995	[M+CH3COO]+ [M+HCOO]+ [M-H]-
SM d35:2	-3.396244667	-3.599983867	-3.5046	-3.8581	-3.067	-4.5587	-4.4548	-3.8192	-5.6433	-4.0202	-3.3189	-4.3331585	-4.7128	SM	714.5623	7.842999	[M*]+ [M+H]+ [M+K]+ [M+Na]+
SM d35:1	-1.771004367	-2.112705233	-3.5348	-2.1741	-1.7698	-3.2676	-4.1668	-2.9645	-4.0716	-3.4248	-3.7916	-3.2624321	-3.8356	SM	716.5803	8.744997	[M*]+ [M+H]+ [M+K]+ [M+Na]+
SM d36:2	-3.3332907	-4.1778889	-4.1179	-5.2274	-3.4704	-4.1383	-3.7983	-3.8922	-5.3683	-5.1328	-4.2408	-4.6346609	-4.6265	SM	728.5809	8.639005	[M*]+ [M+H]+ [M+K]+ [M+Na]+
SM d36:0	-5.322330567	-5.550348933	-4.4666	-5.4493	-4.8866	-5.3895	-3.8922	-3.4429	-4.5755	-4.2733	-3.9524	-3.8432184	-4.6308	SM	732.6128	10.647002	[M+H]+ [M+K]+ [M+Na]+
SM d37:4	-5.965965167	-5.887277667	-6.6433	-6.2805	-5.8913	-6.1768	-7.2065	-6.0171	-6.7018	-6.4511	-6.2794	-6.4138062	-6.8295	SM	738.5711	8.725003	[M*]+ [M+H]+ [M+K]+ [M+Na]+
SM d38:1	-4.363278033	-5.412425433	-6.1902	-5.9523	-4.6245	-5.9137	-6.165	-4.6535	-6.3279	-5.9475	-5.5701	-5.0367743	-6.3187	SM	758.6277	11.893003	[M+H]+ [M+K]+ [M+Na]+
SM d39:1	-6.0692958	-7.666427667	-7.7063	-7.4186	-6.3853	-7.5084	-8.0479	-6.9784	-8.804	-8.1486	-8.26	-7.0667648	-9.0349	SM	772.6424	12.958006	[M+H]+ [M+K]+ [M+NH4]+ [M+Na]+
SM d40:2	-4.389179833	-5.4999592	-5.5589	-5.4491	-4.6674	-4.9318	-6.0528	-4.8224	-6.6241	-6.2846	-5.4855	-5.2465153	-6.5659	SM	784.6429	12.091002	[M+H]+ [M+K]+ [M+Na]+
SM d40:1	-2.894263567	-3.808856333	-3.9176	-3.8357	-3.0655	-3.8477	-3.8744	-2.8535	-4.473	-4.1988	-3.5161	-3.2980207	-4.4696	SM	786.6599	13.911004	[M*]+ [M+H]+ [M+K]+ [M+NH4]+ [M+Na]+
SM d40:0	-5.062789867	-5.8401973	-5.6466	-5.7611	-5.1199	-6.2318	-4.7955	-4.3689	-5.9125	-5.4078	-4.7247	-4.8680891	-6.0197	SM	788.6756	14.690001	[M*]+ [M+H]+ [M+K]+ [M+NH4]+ [M+Na]+
SM d41:3	-3.643069567	-4.416621433	-4.2728	-4.2507	-4.21	-4.0373	-4.5458	-3.2044	-4.9181	-4.4075	-3.887	-3.7760003	-5.5554	SM	796.6397	11.3359995	[M*]+ [M+H]+ [M+K]+ [M+Na]+
SM d41:2	-5.429044067	-6.351362233	-6.1633	-6.3664	-5.8194	-6.0843	-6.839	-5.4467	-7.2697	-6.8467	-6.1556	-5.784017	-7.2515	SM	798.6585	13.050997	[M*]+ [M+H]+ [M+K]+ [M+NH4]+ [M+Na]+
SM d42:4	-5.049537533	-6.011188467	-6.464	-6.1501	-5.3397	-6.1116	-6.0131	-5.0187	-6.8149	-6.454	-5.6854	-5.7645372	-6.6926	SM	808.6423	13.915002	[M*]+ [M+H]+ [M+K]+ [M+NH4]+ [M+Na]+
SM d42:3	-6.262793833	-6.916516667	-7.2468	-6.5512	-6.2253	-7.647	-6.7639	-5.7951	-7.2999	-7.0215	-6.575	-6.361297	-8.0717	SM	810.6647	14.704997	[M*]+ [M+H]+ [M+K]+ [M+NH4]+ [M+Na]+
SM d42:2	-2.658851633	-3.721714667	-2.7856	-3.4401	-3.0843	-2.9745	-3.5577	-2.6377	-4.3335	-4.0716	-3.1241	-2.9952297	-4.128	SM	812.6755	14.010003	[M*]+ [M+H]+ [M+K]+ [M+Na]+
SM d42:1	-3.394555433	-4.242583533	-4.1189	-3.9906	-3.4227	-4.2628	-3.806	-3.2615	-4.744	-4.6781	-3.5641	-3.7605438	-4.6835	SM	814.6917	15.308003	[M*]+ [M+H]+ [M+K]+ [M+NH4]+ [M+Na]+
SM d42:0	-3.393473933	-4.128014833	-4.0801	-4.1434	-3.5332	-4.6476	-3.4451	-3.0052	-4.3682	-3.8825	-3.2334	-4.2287077	-4.5765	SM	816.7075	15.480998	[M+CH3COO]+ [M+HCOO]+ [M-H]-
SM d43:2	-6.042236233	-6.567119567	-6.6604	-6.6378	-6.0361	-6.5063	-7.9427	-6.0057	-7.7758	-7.2276	-6.7968	-7.0047556	-7.9946	SM	826.6899	14.661994	[M*]+ [M+H]+ [M+K]+ [M+Na]+
SM d43:1	-4.2893311	-3.8128325	-4.2321	-3.9825	-3.5466	-3.9153	-4.492	-3.4389	-4.925	-4.4064	-4.7242	-4.7041639	-4.2234	SM	828.7111	15.445005	[M+CH3COO]+ [M+HCOO]+ [M-H]-
SM d44:2	-7.4089197	-8.810631333	-8.4559	-8.9055	-7.6494	-8.5352	-8.6	-8.188	-10.575	-9.8804	-8.8448	-8.1648521	-8.9368	SM	840.7054	15.299005	[M*]+ [M+H]+ [M+K]+ [M+NH4]+ [M+Na]+
TG 12:0_12:0_16:0	-7.253268	-8.107497167	-8.3479	-7.2868	-6.2957	-5.7417	-8.6692	-4.6495	-7.1575	-7.9413	-8.5011	-6.5364927	-8.3651	TG	694.6094	16.218996	[M*]+ [M+H]+ [M+K]+ [M+NH4]+ [M+Na]+
TG 42:2	-9.369375	-10.41422633	-7.2282	-8.5188	-8.9034	-5.8381	-8.8833	-4.0731	-5.7415	-8.1509	-7.5848	-5.9617278	-6.3318	TG	718.6097	16.013	[M*]+ [M+H]+ [M+K]+ [M+NH4]+ [M+Na]+
TG 42:1	-6.875628767	-7.9855479	-6.1109	-6.5324	-6.085	-3.8211	-7.9763	-3.0121	-4.6489	-6.7189	-6.7427	-5.058396	-5.7315	TG	720.6253	16.263	[M*]+ [M+H]+ [M+K]+ [M+NH4]+ [M+Na]+
TG 12:0_14:0_16:0	-5.575120933	-6.462934533	-7.7439	-7.1906	-4.8499	-5.6688	-7.6905	-5.3201	-6.8215	-7.1491	-8.0528	-6.946326	-7.6462	TG	722.6406	16.564003	[M*]+ [M+H]+ [M+K]+ [M+NH4]+ [M+Na]+
TG 12:0_13:0_18:1	-9.177993	-9.408372333	-8.7872	-9.9078	-8.5981	-7.2363	-8.2177	-6.2573	-7.8585	-8.8138	-8.5225	-8.3979195	-9.1958	TG	734.6402	16.434	[M*]+ [M+H]+ [M+K]+ [M+NH4]+ [M+Na]+
TG 14:0_14:0_15:0	-7.112669	-7.617874667	-7.9228	-7.5423	-6.8626	-7.3006	-6.824	-7.0895	-7.3597	-7.8444	-7.8701	-7.9557444	-7.9643	TG	736.6517	16.739994	[M*]+ [M+H]+ [M+K]+ [M+NH4]+ [M+Na]+
TG 44:3	-10.21632533	-11.41294767	-7.5987	-9.4267	-9.9085	-6.9041	-9.3621	-4.3419	-5.8772	-8.1412	-7.5765	-6.2874343	-6.6553	TG	744.6251	16.068995	[M*]+ [M+H]+ [M+K]+ [M+NH4]+ [M+Na]+
TG 44:2	-7.195438967	-8.201145667	-4.974	-7.2873	-6.6411	-3.3821	-6.5152	-3.5292	-4.0442	-6.7302	-5.6469	-5.4124565	-4.3913	TG	746.641	16.314003	[M*]+ [M+H]+ [M+K]+ [M+NH4]+ [M+Na]+
TG 44:1	-4.8070762	-5.8026758	-5.2473	-6.7335	-4.0149	-4.0241	-6.4954	-4.0581	-4.5881	-5.8645	-5.5291	-5.8527373	-5.1087	TG	748.6566	16.619997	[M*]+ [M+H]+ [M+K]+ [M+NH4]+ [M+Na]+
TG 44:0	-3.929341633	-4.593594867	-7.103	-5.8626	-3.7271	-4.6146	-6.7574	-5.2697	-6.0023	-5.9242	-7.0277	-6.2599342	-6.4871	TG	750.6714	16.989994	[M*]+ [M+H]+ [M+K]+ [M+NH4]+ [M+Na]+
TG 45:2	-8.822560333	-9.457388833	-7.4834	-9.6321	-8.0104	-7.6332	-7.9226	-7.3367	-7.3609	-8.0709	-7.6214	-8.2720212	-7.5308	TG	760.6566	16.484991	[M*]+ [M+H]+ [M+K]+ [M+NH4]+ [M+Na]+
TG 45:1	-6.6011512	-7.3912265	-7.6714	-8.0151	-6.2143	-6.502	-6.9788	-6.161	-6.8177	-7.2655	-7.6287	-7.4099439	-7.0607	TG	762.6713	16.814003	[M*]+ [M+H]+ [M+K]+ [M+NH4]+ [M+Na]+
TG 14:0_15:0_16:0	-6.667738667	-6.697877233	-9.0912	-7.0386	-6.5462	-7.1872	-8.3341	-7.3458	-7.7645	-7.472	-8.8559	-7.6071039	-7.8931	TG	764.6854	17.244001	[M*]+ [M+H]+ [M+K]+ [M+NH4]+ [M+Na]+
TG 46:3	-8.032685667	-8.957464667	-5.9332	-9.194	-7.1274	-6.5796	-7.1808	-4.9545	-5.0875	-6.7296	-5.9061	-6.7843188	-5.2769	TG	772.6562	16.422997	[M*]+ [M+H]+ [M+K]+ [M+NH4]+ [M+Na]+
TG 46:2	-4.691736867	-5.635798733	-3.4368	-6.6486	-4.0133	-3.695	-4.8925	-3.7987	-3.6079	-5.1219	-3.9308	-5.3697491	-3.53	TG	774.6722	16.677	[M*]+ [M+H]+ [M+K]+ [M+NH4]+ [M+Na]+
TG 46:1	-2.139996833	-3.038267133	-4.1085	-4.5433	-1.691	-1.9783	-4.9617	-2.8801	-3.3972	-3.7908	-4.1907	-4.0505822	-3.5857	TG	776.6877	17.04101	[M*]+ [M+H]+ [M+K]+ [M+NH4]+ [M+Na]+
TG 46:0	-3.9697202	-4.35794	-7.3606	-5.3006	-4.3169	-4.8222	-6.1915	-5.4208	-5.7443	-5.7065	-6.634	-5.761036	-6.2326	TG	778.7032	17.491007	[M*]+ [M+H]+ [M+K]+ [M+NH4]+ [M+Na]+
TG 12:0_17:1_18:1	-7.046708433	-7.553340967	-6.8969	-8.3486	-6.5599	-6.5151	-6.6185	-5.9155	-6.8885	-7.0559	-6.5647	-6.78066	-6.9039	TG	788.6867	16.864008	[M*]+ [M+K]+ [M+NH4]+ [M+Na]+
TG 14:0_15:0_18:1	-4.520748833	-5.219618467	-6.6376	-6.2089	-4.2703	-4.9766	-5.9397	-4.7236	-5.8027	-5.739	-6.5399	-5.5341555	-6.1289	TG	790.7018	17.294004	[M*]+ [M+K]+ [M+NH4]+ [M+Na]+
TG 15:0_16:0_16:0	-5.7545178	-5.9533214	-7.9332	-6.1931	-6.0763	-6.879	-7.262	-6.4739	-6.9533	-6.5527	-7.4691	-6.5590681	-7.4573	TG	792.7187	17.825006	[M*]+ [M+K]+ [M+NH4]+ [M+Na]+
TG 48:4	-7.312100667	-7.817295533	-6.4675	-8.7976	-5.3893	-6.9928	-7.2833	-4.2844	-4.7083	-5.7502	-5.3355	-5.4872602	-4.9331	TG	798.6718	16.57099	[M*]+ [M+K]+ [M+NH4]+ [M+Na]+
TG 48:3	-5.053553233	-5.952742267	-4.2382	-6.7421	-4.3665	-4.2823	-4.8432	-3.3701	-3.6283	-4.7458	-4.1601	-4.6275424	-3.3907	TG	800.6872	16.757998	[M*]+ [M+H]+ [M+K]+ [M+NH4]+ [M+Na]+
TG 48:2	-2.159091933	-3.075256967	-2.1031	-4.2744	-1.7627	-1.5082	-3.2089	-2.0528	-2.4106	-3.1164	-2.6009	-3.0412197	-2.1303	TG	802.7028	17.104998	[M*]+ [M+H]+ [M+K]+ [M+NH4]+ [M+Na]+
TG 14:0_16:0_18:1	-1.3054212	-2.1710434	-4.2994	-3.6931	-1.0695	-1.4682	-4.4141	-1.9097	-2.9831	-2.8838	-4.0872	-3.034516	-3.5778	TG	804.7198	17.573996	[M*]+ [M+H]+ [M+K]+ [M+NH4]+ [M+Na]+
TG 48:0	-4.012750667	-3.5376002	-6.3228	-3.559	-4.9192	-4.7586	-5.3447	-4.8959	-4.375	-4.6206	-5.2882	-4.6863302	-4.7752	TG	806.735	18.159994	[M*]+ [M+K]+ [M+NH4]+ [M+Na]+
TG 49:3	-8.075164	-8.965997	-7.3734	-9.5442	-7.794	-7.7683	-7.4207	-5.8884	-6.1819	-7.5562	-6.5548	-6.1784617	-6.1817	TG	814.702	16.960001	[M*]+ [M+H]+ [M+K]+ [M+NH4]+ [M+Na]+

TG 49:2	-4.354924533	-5.187224667	-5.2599	-6.1465	-4	-4.2092	-4.8567	-3.5481	-4.7617	-4.662	-5.317	-4.4456501	-4.8579	TG	816.7177	17.359991	[M*]+ [M+H]+ [M+K]+ [M+NH4]+ [M+Na]+
TG 49:1	-2.7288793	-3.519487367	-5.9459	-4.7117	-2.6037	-3.2811	-4.7455	-2.6919	-3.7887	-3.6255	-4.7701	-3.5718162	-4.5477	TG	818.7351	17.882998	[M*]+ [M+H]+ [M+K]+ [M+NH4]+ [M+Na]+
TG 16:0_16:0_17:0	-7.226681367	-6.968406667	-8.8437	-6.3414	-6.8754	-7.7111	-7.8843	-6.4284	-6.8572	-7.0928	-7.7294	-6.668787	-7.4832	TG	820.7499	18.564005	[M*]+ [M+H]+ [M+K]+ [M+NH4]+ [M+Na]+
TG 50:6	-6.0139225	-6.924164533	-5.6776	-8.5119	-4.8266	-5.6686	-7.5784	-3.8233	-4.463	-5.0768	-4.8994	-4.6292177	-5.117	TG	822.6725	16.442995	[M*]+ [M+K]+ [M+NH4]+ [M+Na]+
TG 50:5	-5.6885351	-6.571187367	-5.1336	-7.7154	-4.9391	-5.1098	-6.3684	-2.8085	-3.695	-4.1302	-4.1811	-2.9084053	-4.0628	TG	824.6877	16.687006	[M*]+ [M+K]+ [M+NH4]+ [M+Na]+
TG 50:4	-6.185574767	-6.787691133	-5.1228	-7.3636	-5.3817	-5.3073	-6.0475	-4.147	-4.6784	-5.2952	-5.0681	-4.9261684	-4.7561	TG	826.7025	16.825994	[M*]+ [M+H]+ [M+K]+ [M+NH4]+ [M+Na]+
TG 50:3	-2.875914867	-3.692303967	-2.3827	-4.5609	-2.4753	-2.1174	-2.2106	-1.5778	-2.243	-2.8197	-3.2893	-2.4050923	-2.6968	TG	828.718	17.174006	[M*]+ [M+H]+ [M+K]+ [M+NH4]+ [M+Na]+
TG 50:2	-0.768362047	-1.568259567	-1.2616	-2.742	-0.4318	-0.1454	-1.4464	-0.349	-0.9622	-1.4073	-1.3581	-1.2671897	-1.1217	TG	830.7357	17.655008	[M*]+ [M+H]+ [M+K]+ [M+NH4]+ [M+Na]+
TG 16:0_16:0_18:1	0.103007633	-0.4573911	-2.7354	-1.7671	0.1041	-0.3189	-2.4997	-0.0719	-0.982	-0.862	-2.1478	-0.9504738	-1.7704	TG	832.7514	18.218996	[M*]+ [M+H]+ [M+K]+ [M+NH4]+ [M+Na]+
TG 16:1_17:1_18:1	-5.2811769	-6.194242467	-4.6405	-6.7339	-4.6268	-4.5196	-4.6937	-3.3263	-4.1258	-4.5458	-4.4855	-4.1487776	-4.0853	TG	842.7341	17.435995	[M*]+ [M+H]+ [M+K]+ [M+NH4]+ [M+Na]+
TG 16:0_17:1_18:1	-2.538505567	-3.310766233	-2.8111	-4.3636	-2.0023	-1.864	-2.4493	-1.4169	-2.2507	-2.6566	-2.5571	-2.4379717	-2.5321	TG	844.7511	17.94399	[M*]+ [M+H]+ [M+K]+ [M+NH4]+ [M+Na]+
TG 51:1	-4.011009833	-5.1587505	-6.8631	-5.923	-3.9488	-4.4	-6.2946	-3.5602	-4.6272	-4.5528	-5.8089	-4.3219929	-5.3959	TG	846.7659	18.585001	[M*]+ [M+H]+ [M+K]+ [M+NH4]+ [M+Na]+
TG 52:7	-6.031499933	-6.945126333	-5.1779	-8.3282	-5.0113	-5.4108	-6.5672	-3.1694	-4.0308	-4.4437	-4.2747	-3.6764126	-4.5353	TG	848.6881	16.489994	[M*]+ [M+K]+ [M+NH4]+ [M+Na]+
TG 14:0_16:0_22:6	-3.4340655	-4.324772533	-3.6586	-6.468	-2.8238	-4.1321	-4.5571	-1.8214	-2.7372	-3.5454	-3.076	-1.3891531	-3.2834	TG	850.7036	16.744997	[M*]+ [M+H]+ [M+K]+ [M+NH4]+ [M+Na]+
TG 16:1_18:1_18:2	-4.325068133	-4.3941613	-2.7512	-4.7848	-3.1962	-2.6281	-3.016	-1.3773	-2.107	-2.9674	-2.7543	-2.1578548	-2.2423	TG	854.7334	17.278994	[M*]+ [M+H]+ [M+K]+ [M+NH4]+ [M+Na]+
TG 16:1_18:1_18:1	-1.256034833	-2.069893533	0.0982	-2.7069	-0.7089	0.0655	0.2834	0.3385	-0.0759	-0.9493	-0.1436	-0.5556075	0.0289	TG	856.7513	17.728006	[M*]+ [M+H]+ [M+K]+ [M+NH4]+ [M+Na]+
TG 16:0_18:1_18:1	0.24974823	-0.43859418	-0.2013	-1.3613	0.5783	0.6868	-0.1315	0.967	0.4693	-0.0695	0.1702	0.0408713	0.2639	TG	858.767	18.295	[M*]+ [M+H]+ [M+K]+ [M+NH4]+ [M+Na]+
TG 17:1_18:1_18:1	-4.275062467	-4.820599867	-2.2864	-5.6157	-3.5416	-2.1733	-2.1876	-1.9711	-2.2493	-3.6492	-2.2966	-2.9714979	-2.2943	TG	870.7666	18.007998	[M*]+ [M+H]+ [M+K]+ [M+NH4]+ [M+Na]+
TG 53:2	-5.55579	-6.636232967	-5.1336	-7.8277	-5.1879	-4.5632	-4.9937	-4.3271	-4.634	-5.7505	-4.9711	-5.421189	-4.8232	TG	872.7812	18.58	[M*]+ [M+H]+ [M+K]+ [M+NH4]+ [M+Na]+
TG 54:8	-6.6846192	-7.483688867	-5.4388	-8.2588	-5.8076	-5.8479	-6.1681	-2.7788	-3.9825	-4.1502	-4.2233	-2.9704444	-4.6263	TG	874.703	16.552	[M*]+ [M+H]+ [M+K]+ [M+NH4]+ [M+Na]+
TG 54:7	-3.551961233	-4.3120829	-3.0821	-5.8379	-2.6745	-2.7574	-3.6706	-1.3702	-2.0489	-2.1781	-2.3037	-2.4828269	-2.6611	TG	876.7189	16.849	[M*]+ [M+H]+ [M+K]+ [M+NH4]+ [M+Na]+
TG 16:0_18:1_20:5	-3.771104867	-4.847317367	-2.2546	-5.9422	-3.2985	-2.9996	-2.2971	-0.4643	-1.0624	-1.7124	-1.4147	-1.2531719	-1.6793	TG	878.7343	17.15301	[M*]+ [M+H]+ [M+K]+ [M+NH4]+ [M+Na]+
TG 54:4	-2.4985638	-3.331675167	-0.4436	-3.419	-1.8458	-0.3617	-0.5465	0.0082	-0.1945	-1.5721	-0.3582	-0.9320997	-0.2718	TG	882.7669	17.808	[M*]+ [M+K]+ [M+NH4]+ [M+Na]+
TG 18:1_18:1_18:1	-0.656089793	-1.828090667	1.3847	-2.118	-0.3818	1.2324	1.5119	0.5935	0.8692	-0.8522	1.1739	-0.4626439	1.1095	TG	884.7826	18.401999	[M*]+ [M+H]+ [M+K]+ [M+NH4]+ [M+Na]+
TG 18:1_18:2_19:1	-8.262153	-8.59385	-5.8459	-8.3477	-6.4102	-5.5931	-6.0842	-4.0711	-5.4767	-6.0472	-5.6295	-5.2834669	-5.9946	TG	896.7803	18.119001	[M*]+ [M+H]+ [M+K]+ [M+NH4]+ [M+Na]+
TG 56:8	-4.6594531	-5.3208815	-3.4478	-6.4469	-3.6663	-3.4204	-4.0338	-1.3256	-3.0283	-2.1785	-2.4887	-2.009372	-3.6918	TG	902.734	16.943995	[M*]+ [M+H]+ [M+K]+ [M+NH4]+ [M+Na]+
TG 56:7	-2.139832167	-2.905471833	-2.9213	-4.5293	-1.1976	-1.4558	-3.1395	-0.4396	-1.3398	-1.1425	-1.8012	-1.2694384	-2.4349	TG	904.7496	17.336	[M*]+ [M+K]+ [M+NH4]+ [M+Na]+
TG 56:5	-4.739195533	-4.063999767	-2.4503	-6.0685	-3.274	-2.8059	-2.7759	-2.2175	-2.579	-3.8648	-2.567	-2.9384721	-3.0252	TG	908.7819	17.958006	[M*]+ [M+H]+ [M+K]+ [M+NH4]+ [M+Na]+
TG 18:1_18:1_20:2	-5.483694067	-6.343381267	-3.9373	-6.2226	-4.6466	-3.5993	-4.5184	-2.9716	-4.0237	-4.6531	-4.3733	-4.0659943	-4.694	TG	910.7967	18.55601	[M*]+ [M+H]+ [M+K]+ [M+NH4]+ [M+Na]+
TG 58:12	-9.333785333	-9.8729145	-8.2985	-11.494	-7.9227	-8.5118	-9.9625	-4.6085	-6.0316	-5.6824	-6.0719	-5.3230393	-7.8382	TG	922.7037	16.312994	[M*]+ [M+K]+ [M+NH4]+ [M+Na]+
TG 58:11	-7.327782867	-8.065139	-5.9162	-8.8261	-6.6288	-6.5758	-6.4581	-2.2774	-3.8472	-3.5757	-4.0221	-3.242787	-5.3288	TG	924.7192	16.549006	[M*]+ [M+H]+ [M+K]+ [M+NH4]+ [M+Na]+
TG 58:10	-7.163925167	-8.688527	-4.8635	-9.8067	-6.0457	-5.5712	-4.9617	-2.2108	-3.4273	-3.6394	-3.0976	-3.1797288	-4.3085	TG	926.7344	16.729002	[M*]+ [M+H]+ [M+K]+ [M+NH4]+ [M+Na]+
TG 58:9	-5.435875433	-6.236124667	-4.1262	-6.5008	-4.5915	-4.3035	-3.9429	-1.276	-2.4138	-2.6445	-2.5978	-2.2452888	-3.476	TG	928.7492	17	[M*]+ [M+H]+ [M+K]+ [M+NH4]+ [M+Na]+
TG 18:1_18:2_22:5	-7.3356972	-8.145928667	-5.6099	-8.5367	-6.3613	-5.6787	-6.8257	-3.2306	-4.9605	-4.8811	-5.3992	-4.0318037	-6.1136	TG	930.7628	17.174006	[M*]+ [M+H]+ [M+K]+ [M+NH4]+ [M+Na]+
TG 58:7	-4.689435233	-5.2281487	-4.9442	-6.6029	-3.4862	-3.4694	-3.9822	-2.085	-3.2308	-3.4692	-3.7411	-3.2866402	-4.4277	TG	932.7817	17.939005	[M*]+ [M+K]+ [M+NH4]+ [M+Na]+
TG 58:6	-5.387346933	-5.948433567	-2.703	-6.2226	-4.3915	-3.3961	-2.9055	-2.3534	-4.1274	-4.0326	-3.9828	-3.284193	-3.904	TG	934.7972	18.183	[M*]+ [M+H]+ [M+K]+ [M+NH4]+ [M+Na]+
TG 60:12	-7.618525667	-8.933768667	-5.5274	-9.7794	-7.2009	-6.7882	-6.4925	-3.1351	-4.5167	-4.5866	-3.7509	-4.0228087	-5.5766	TG	950.7345	16.588007	[M*]+ [M+H]+ [M+K]+ [M+NH4]+ [M+Na]+
TG 62:13	-8.213945	-8.938683	-6.6404	-9.6743	-7.0073	-6.7207	-7.9461	-4.1247	-5.5773	-5.0779	-5.043	-5.0991077	-7.3708	TG	976.7489	16.726007	[M*]+ [M+NH4]+
TG 18:1_22:5_22:6	-8.198062167	-9.040402	-6.3037	-9.8045	-6.9682	-6.6993	-7.7899	-4.3272	-5.9451	-5.7095	-5.5828	-5.1209686	-7.3122	TG	978.7639	16.877995	[M*]+ [M+H]+ [M+NH4]+

Suppl. Table S2. Number of differentially expressed lipids in the different treatments.

Treatments in which MLT cytoprotective effects were investigated are shown in bold. Statistical significance was investigated by moderated t-test and differential expression was identified by fold-change values as described in the text.
Cell conditions where MLT was used are highlighted in bold.

	Treatment	Upregulated Lipids	Downregulated Lipids	Total lipids
**Individual treatments**	**MLT Vs CTL**	**1**	**158**	**159**
	Cd Vs CTL	28	69	97
	PA Vs CTL	5	148	153
	OA Vs CTL	40	100	140
	OAPA Vs CTL	49	135	184
**Combinatorial treatments**	**Cd+MLT Vs CTL**	**28**	**154**	**182**
	Cd+PA Vs CTL	72	80	152
	Cd+OA Vs CTL	40	141	181
	Cd+OAPA Vs CTL	49	163	212
	**Cd+PA+MLT Vs CTL**	**66**	**100**	**166**
	**Cd+OA+MLT Vs CTL**	**59**	**101**	**160**
	**Cd+OAPA+MLT Vs CTL**	**37**	**182**	**219**
**Combinatorial treatments (MLT effect on lipotoxicity agents)**	**Cd+MLT Vs Cd**	**16**	**168**	**184**
	**Cd+PA+MLT Vs Cd+PA**	**14**	**56**	**70**
	**Cd+OA+MLT Vs Cd+OA**	**113**	**11**	**124**
	**Cd+OAPA+MLT Vs Cd+OAPA**	**4**	**55**	**59**

Suppl. Table S3. Top 20 significantly down- and upregulated lipid species revealed by pairwise expression analysis between lipotoxicity treatments and MLT cytoprotective effect compared to control cell condition.

Suppl. Table S3A. MLT Vs CTL top 20 downregulated lipids		
Compound	-Log10 adj pvalue	Log2 FC
LPC 18:3/0:0	5.3295	-1.5895
PC 22:6_22:6	2.7547	-1.4853
PC 38:2	2.1914	-1.4524
PC 37:6	2.3960	-1.4307
FAHFA 18:0_20:2	5.1722	-1.2933
PC 31:1	2.1914	-1.2852
PC 38:7	2.7371	-1.2649
PC 36:7	3.2313	-1.2253
TG 44:3	4.0111	-1.1966
LPC 16:1/0:0	5.3295	-1.1885
LPC 16:0/0:0	5.0148	-1.1735
TG 18:1_18:1_18:1	5.3295	-1.1720
PC 34:4	4.7022	-1.1313
SM d43:2	3.6002	-1.1286
PC 39:7	4.4016	-1.1179
SM d40:2	4.0201	-1.1108
TG 42:1	4.4109	-1.1099
LPC 18:0/0:0	4.9645	-1.1011
ACar 20:0	4.1101	-1.0814
TG 53:2	5.2533	-1.0804

Suppl. Table S3B. MLT Vs CTL top 20 upregulated lipids		
Compound	-Log10 adj pvalue	Log2 FC
TG 56:5	2.5795	0.6752

Suppl. Table S3C. Cd Vs CTL top 20 downregulated lipids		
Compound	-Log10 adj pvalue	Log2 FC
FAHFA 18:1_20:3	4.7649	-3.3972
PG 18:1_18:2	4.7649	-3.2384
Cer_NS d18:2_14:0	4.6448	-3.0131
PC 14:0_16:0	6.0084	-2.4152
PG 16:0_18:1	6.4431	-2.4150
Cer_NS d18:2_22:0	3.8472	-2.2837
PE 18:1_18:2	2.9452	-2.0899
Cer_NS d18:2_16:0	4.0203	-2.0556
PE 16:0_20:5	5.9135	-2.0339
Cer_NDS d16:0_26:2	3.6990	-1.9635
FA 19:1	3.6990	-1.9419
PI 16:0_20:5	4.8878	-1.8228
Cer_NS d16:1_16:0	3.8604	-1.8093
FA 18:1	3.7599	-1.7879
PE 16:0_20:4	6.0485	-1.7772
PE 18:0_17:1	5.2108	-1.7454
PE 20:0_20:4	3.2415	-1.6778
PC 18:1_18:2	3.8604	-1.6764
ACar 20:0	4.2254	-1.6579
LPC 16:0/0:0	3.3531	-1.6553

Suppl. Table S3D. Cd Vs CTL top 20 upregulated lipids		
Compound	-Log10 adj pvalue	Log2 FC
Cer_NDS d18:0_18:0	3.6990	2.1398
TG 48:4	2.3241	1.9228
TG 58:12	3.9920	1.4111
FA 16:0	2.5438	1.2854
TG 18:1_22:5_22:6	4.1867	1.2299
TG 62:13	4.6658	1.2067
TG 58:7	3.6047	1.2033
FA 18:0	3.0271	1.1911
TG 50:6	4.7622	1.1874
TG 52:7	4.6658	1.0202
TG 45:2	2.3999	1.0009
TG 58:6	2.2496	0.9959
TG 56:8	4.1541	0.9932
TG 12:0_12:0_16:0	4.1417	0.9576
TG 56:7	4.2254	0.9422
TG 46:3	4.1867	0.9053
TG 54:7	4.4469	0.8774
TG 54:8	4.7622	0.8770
DG 18:1_22:6	2.2160	0.8758
TG 58:9	4.4243	0.8444

Suppl. Table S3E. Cd+MLT Vs CTL top 20 downregulated lipids		
Compound	-Log10 adj pvalue	Log2 FC
PC 38:2	2.0047	-3.2556
LPC 18:3/0:0	6.7447	-3.2555
Cer_NS d18:2_14:0	4.4574	-3.0545
LPE 16:0	6.6691	-3.0221
LPC 16:0/0:0	6.9137	-2.9725
PG 16:0_18:1	4.8418	-2.7117
Cer_NS d18:2_24:1	5.5517	-2.6681
PC 14:0_16:0	5.9660	-2.6634
PC 29:0	6.4657	-2.6563
BMP 18:1_22:6	5.6807	-2.5976
PC 30:1	6.2692	-2.5882
SM d32:2	6.6103	-2.5577
PC 42:6	3.5508	-2.4521
FAHFA 18:1_20:3	4.1267	-2.3993
LPC 0:0/18:2	3.9626	-2.3629
Cer_NS d18:2_22:0	3.9081	-2.3607
ACar 20:0	5.1991	-2.2959
SM d41:2	5.8721	-2.2676
Cer_NDS d16:0_26:2	3.9341	-2.2495
SM d30:1	5.0515	-2.2453
ACar 20:1	5.3954	-2.2404
PE 16:0_20:5	5.6955	-2.2229

Suppl. Table S3F. Cd+MLT Vs CTL top 20 upregulated lipids		
Compound	-Log10 adj pvalue	Log2 FC
TG 58:11	2.6668	3.7521
TG 58:12	6.5282	3.6514
TG 58:10	3.2476	3.5244
TG 62:13	6.6103	3.1360
TG 60:12	2.0883	3.0318
Cer_NDS d18:0_18:0	4.0633	2.8333
TG 58:9	6.6103	2.7914
TG 54:8	5.0449	2.5344
TG 18:1_22:5_22:6	5.7072	2.4886
TG 56:8	6.5282	2.4809
TG 18:1_18:2_22:5	2.8627	2.4545
TG 44:3	5.6084	2.0751
TG 16:0_18:1_20:5	3.2832	2.0587
PC 20:5_22:6	2.1853	1.9540
TG 52:7	6.0615	1.5878
TG 48:4	2.1537	1.5619
TG 50:5	5.1335	1.5583
TG 54:7	5.8721	1.3738
TG 58:6	2.9129	1.3547
TG 46:3	5.8782	1.3030
TG 58:7	3.1571	1.2201
TG 42:2	3.6020	1.2184

Suppl. Table S3G. PA Vs CTL top 20 downregulated lipids		
Compound	-Log10 adj pvalue	Log2 FC
TG 14:0_16:0_22:6	3.5834	-3.0340
ACar 20:0	5.5291	-2.6893
TG 58:10	2.7193	-2.6428
TG 50:6	6.0322	-2.4980
TG 46:1	6.0553	-2.4033
TG 56:7	6.1643	-2.3895
TG 14:0_16:0_18:1	6.4478	-2.3876
TG 52:7	5.6989	-2.2967
TG 54:7	4.9279	-2.2859
TG 53:2	2.9415	-2.2719
TG 16:0_18:1_20:5	2.8117	-2.1711
TG 58:12	3.0553	-2.1603
TG 48:2	5.6268	-2.1153
TG 50:5	3.2568	-2.0268
TG 49:1	6.4478	-1.9828
TG 50:2	6.1643	-1.9736
TG 46:2	5.7031	-1.9568
TG 44:0	5.7599	-1.9333
TG 44:1	5.7632	-1.9264
TG 58:7	4.3666	-1.9134

Suppl. Table S3H. PA Vs CTL top 20 upregulated lipids		
Compound	-Log10 adj pvalue	Log2 FC
CE 22:6	3.5973	1.1691
PC 20:5_22:6	2.3734	1.1379
PC 16:1_19:2	2.3685	1.0456
TG 42:2	2.8489	0.8506
TG 44:3	3.2520	0.7897

Suppl. Table S3I. OA Vs CTL top 20 downregulated lipids		
Compound	-Log10 adj pvalue	Log2 FC
ACar 20:0	6.4769	-5.5967
ACar 18:0	6.1169	-4.3779
LPC 18:3/0:0	7.0020	-3.9351
LPE 16:0	6.7676	-3.6609
Cer_NS d18:2_22:0	5.1340	-3.4357
TG 46:0	3.1864	-3.3909
ACar 16:0	6.0794	-3.3637
PC 38:6	3.3489	-3.3219
TG 49:1	7.0020	-3.2170
TG 44:0	6.3060	-3.1737
TG 14:0_16:0_18:1	6.1409	-2.9940
TG 51:1	6.5540	-2.8521
TG 16:0_16:0_18:1	6.5540	-2.8384
TG 50:1	6.7256	-2.8245
PG 16:0_18:1	5.2944	-2.7782
PC 34:5	6.1105	-2.7404
PC 36:5	5.7335	-2.7300
PC 29:0	6.5043	-2.6035
PC 36:7	2.1464	-2.5807
TG 14:0_15:0_16:0	2.4838	-2.4234

Suppl. Table S3J. OA Vs CTL top 20 upregulated lipids		
Compound	-Log10 adj pvalue	Log2 FC
TG 58:6	4.1784	2.6843
TG 44:3	4.4617	2.6176
DG 18:1_18:1	4.9180	2.4484
TG 58:10	2.5679	2.3005
TG 56:5	4.6739	2.2889
TG 44:2	6.5043	2.2214
TG 42:2	4.7212	2.1412
TG 46:3	5.8813	2.0994
TG 54:4	6.5043	2.0550
TG 18:1_18:1_18:1	6.5043	2.0408
TG 17:1_18:1_18:1	3.4702	1.9887
DG 18:1_18:2	5.9003	1.9870
TG 18:1_22:5_22:6	4.2531	1.8944
TG 18:1_18:2_22:5	2.3365	1.7258
TG 62:13	4.6045	1.5736
DG 18:1_20:3	2.8329	1.5536
TG 18:1_18:1_20:2	4.1265	1.5464
TG 16:0_18:1_20:5	2.8204	1.5165
DG 18:1_22:5	3.5571	1.5017
SM d42:1	2.9138	1.4998

Suppl. Table S3K. OAPA Vs CTL top 20 downregulated lipids		
Compound	-Log10 adj pvalue	Log2 FC
FAHFA 18:1_20:3	4.7649	-3.3972
PG 18:1_18:2	4.7649	-3.2384
Cer_NS d18:2_14:0	4.6448	-3.0131
PC 14:0_16:0	6.0084	-2.4152
PG 16:0_18:1	6.4431	-2.4150
LPC 18:3/0:0	6.1306	-2.4131
PC 36:7	2.2184	-2.3200
Cer_NS d18:2_22:0	3.8472	-2.2837
PC 17:2_18:3	4.2086	-2.2412
LPC 16:0/0:0	6.3013	-2.2198
PE 18:1_18:2	2.9452	-2.0899
Cer_NS d18:2_16:0	4.0615	-2.0485
PE 16:0_20:5	5.9135	-2.0339
Cer_NDS d16:0_26:2	3.6990	-1.9635
PC 33:5	2.8494	-1.9609
FA 19:1	3.6990	-1.9419
LPE 16:0	5.8438	-1.8298
PI 16:0_20:5	4.8878	-1.8228
Cer_NS d16:1_16:0	3.8604	-1.8212
PC 37:6	2.6395	-1.7912

Suppl. Table S3L. OAPA Vs CTL top 20 upregulated lipids		
Compound	-Log10 adj pvalue	Log2 FC
TG 44:2	6.5994	3.8133
TG 42:2	4.8126	3.5313
TG 44:3	5.5056	3.3123
TG 42:1	5.8438	3.0545
TG 18:1_18:2_19:1	2.1068	2.6691
DG 18:1_18:1	4.5145	2.1891
Cer_NDS d18:0_18:0	3.6990	2.1398
TG 54:4	6.1306	2.1369
TG 17:1_18:1_18:1	3.5901	2.1018
TG 58:6	3.3111	1.9912
TG 12:0_13:0_18:1	2.8098	1.9417
TG 56:5	4.1687	1.9333
TG 18:1_18:1_18:1	6.1306	1.8885
TG 18:1_18:1_20:2	4.3182	1.8844
DG 18:1_18:2	3.9869	1.7195
TG 18:1_18:2_22:5	2.0652	1.6570
TG 45:2	3.1367	1.5691
TG 12:0_12:0_16:0	4.8852	1.5115
TG 18:1_22:5_22:6	4.6633	1.4987
TG 62:13	4.5432	1.4933

Suppl. Table S3M. Cd+PA Vs CTL top 20 downregulated lipids		
Compound	-Log10 adj pvalue	Log2 FC
PE 16:0_20:5	7.5887	-4.865718
SM d43:2	2.0160	-4.670447
PE 16:0_20:4	7.2186	-4.1835527
PC 14:0_16:0	7.5440	-4.029003
Cer_NS d18:2_22:0	7.5427	-3.7361817
Cer_NS d18:2_14:0	6.5975	-3.5434818
PE 16:0_20:3	2.9905	-3.2607598
PC 16:0_20:5	6.8196	-2.8711705
PE 18:0_22:6	4.6421	-2.8400853
Cer_NS d18:2_24:1	7.5887	-2.6244915
PI 16:0_20:4	6.6418	-2.4616947
LPC 16:0/0:0	5.9720	-2.370727
SM d34:2	7.5887	-2.3612847
Cer_NDS d16:0_26:2	5.2857	-2.3291416
PE 20:0_20:4	2.0821	-2.277036
ACar 20:0	4.4299	-2.2221107
PE 32:1	2.1229	-2.2130044
Cer_NS d18:2_16:0	7.5887	-2.1960049
SM d32:1	7.5887	-2.1107304
PI 16:0_20:5	2.2842	-2.056026

Suppl. Table S3N. Cd+PA Vs CTL top 20 upregulated lipids		
Compound	-Log10 adj pvalue	Log2 FC
TG 44:3	6.9079	5.8743753
TG 42:2	6.9608	5.2962346
TG 58:11	3.1620	5.050407
TG 58:10	3.7848	4.953147
TG 58:12	5.6994	4.7253075
TG 60:12	2.7096	4.4834433
TG 18:1_18:2_19:1	2.8147	4.191053
TG 58:9	6.9608	4.1599236
TG 18:1_18:2_22:5	3.7079	4.1051083
TG 62:13	6.9608	4.0892615
TG 54:8	6.8154	3.900481
TG 18:1_22:5_22:6	6.3234	3.8708644
TG 42:1	6.4442	3.863572
TG 44:2	6.9608	3.666224
TG 56:8	6.9079	3.3338966
TG 16:0_18:1_20:5	4.0500	3.3068135
TG 46:3	6.9608	3.0782251
TG 58:6	4.0543	3.0339837
TG 48:4	3.2515	3.0277185
TG 16:1_18:1_18:2	2.6960	2.947785

Suppl. Table S3O. Cd+OA Vs CTL top 20 downregulated lipids		
Compound	-Log10 adj pvalue	Log2 FC
BMP 22:6_22:6	-52.6117	-5.7173
PE 16:0_20:5	-29.1559	-4.8657
ACar 20:0	-25.8415	-4.6916
PC 34:5	-22.2310	-4.4745
PC 36:7	-17.0863	-4.0948
PC 29:0	-16.7884	-4.0694
PC 14:0_16:0	-16.3249	-4.0290
Cer_NS d18:2_22:0	-13.3261	-3.7362
PC 33:5	-12.8065	-3.6788
Cer_NS d18:2_14:0	-11.6599	-3.5435
ACar 18:0	-10.3724	-3.3747
LPE 16:0	-9.9880	-3.3202
PC 30:1	-9.7936	-3.2918
PE 16:0_20:3	-9.5849	-3.2608
PC 32:3	-9.4129	-3.2346
PC 36:5	-8.6663	-3.1154
TG 14:0_16:0_18:1	-8.6257	-3.1086
PE 32:1	-7.5352	-2.9136
PE 42:5	-7.4645	-2.9000
PC 16:0_20:5	-7.3166	-2.8712

Suppl. Table S3P. Cd+OA Vs CTL top 20 upregulated lipids		
Compound	-Log10 adj pvalue	Log2 FC
Cer_NDS d18:0_16:0	7.5887	2.7288
FAHFA 18:1_20:3	7.5252	2.5127
TG 58:6	3.7078	2.4819
DG 18:1_18:1	4.6725	2.2912
TG 58:10	2.3692	2.2022
TG 18:1_18:1_18:1	6.9010	2.1680
TG 17:1_18:1_18:1	3.6471	2.0875
PI 18:1_18:1	6.7007	2.0238
TG 56:5	3.7407	1.9633
TG 54:4	6.4496	1.9521
PC 20:5_22:6	2.4673	1.9250
TG 16:1_18:1_18:1	6.3516	1.5394
CE 22:6	3.4525	1.4967
TG 58:9	4.8300	1.4930
TG 16:0_18:1_20:5	2.6545	1.4740
SM d36:0	2.2198	1.4302
SM d34:0	6.3821	1.2862
DG 18:1_18:2	4.5993	1.2859
PC 42:10	2.9897	1.2242
PC 42:9	4.7636	1.2119

Suppl. Table S3Q. Cd+OAPA Vs CTL top 20 downregulated lipids		
Compound	-Log10 adj pvalue	Log2 FC
ACar 20:0	5.7186	-4.9251
ACar 18:0	6.1937	-4.2153
ACar 16:0	6.2177	-4.1042
PC 29:0	6.5217	-3.8217
PE 16:0_20:5	7.3330	-3.6881
ACar 20:1	5.8451	-3.6751
PC 14:0_16:0	7.8111	-3.6559
BMP 22:6_22:6	3.1876	-3.5581
PC 30:1	5.7323	-3.5552
PC 34:5	4.1731	-3.4888
PC 33:5	2.8206	-3.4844
PE 16:0_20:4	6.6816	-3.1948
LPE 16:0	6.8979	-3.1936
SM d44:2	3.4926	-3.1663
Cer_NS d18:2_14:0	6.3433	-3.1395
LPC 18:3/0:0	6.8979	-3.1229
PE 32:1	2.6443	-3.0028
PC 32:3	6.5991	-2.9749
PC 36:7	4.6634	-2.9744
BMP 18:1_22:6	3.6414	-2.8993

Suppl. Table S3R. Cd+OAPA Vs CTL top 20 upregulated lipids		
Compound	-Log10 adj pvalue	Log2 FC
Cer_NDS d18:0_16:0	6.0973	4.3391
Cer_NDS d18:0_22:0	3.4040	3.7366
DG 18:1_18:1	6.8979	3.6279
DG 18:1_18:2	2.6070	3.4806
DG 18:1_22:5	6.2022	3.3022
DG 18:1_22:6	6.5217	3.1513
FAHFA 18:1_20:3	2.1809	3.1018
MGDG 13:1_18:1	6.5217	3.0220
PC 20:5_22:6	5.9905	2.9452
PI 18:0_20:3	2.1156	2.7854
PI 18:1_18:1	3.7642	2.7087
TG 14:0_16:0_22:6	4.9654	2.7045
TG 16:0_18:1_20:5	6.5217	2.6367
TG 16:1_17:1_18:1	3.0647	2.6038
TG 16:1_18:1_18:1	2.8599	2.3752
TG 16:1_18:1_18:2	6.5217	2.3041
TG 17:1_18:1_18:1	5.6925	2.2530
TG 18:1_18:1_18:1	5.5583	2.2267
TG 18:1_18:1_20:2	2.2997	2.2181
TG 18:1_18:2_19:1	3.2299	2.1602

Supplementary Table S4. Significantly downregulated and upregulated lipid species during the MLT cytoprotective effect in HepaRG hepatocytes exposed to Cd and FFA-induced lipotoxicity (Cd+FFA) *.

* Top-20 significantly different annotations are listed as identified by pairwise expression analysis.

A

Cd+PA+MLT Vs Cd+PA					
Upregulated lipids			Downregulated lipids		
Compound	-Log10 adj pvalue	Log2 FC	Compound	-Log10 adj pvalue	Log2 FC
FA 20:1	5.8986	2.3724	PI 16:0_20:5	4.3101	-2.1366
FA 22:0	5.8987	2.1377	TG 58:12	2.6860	-1.8066
PC 36:6	2.6727	1.6400	TG 62:13	5.0169	-1.7935
SM d44:2	2.3347	1.6385	TG 58:11	4.2600	-1.4817
FAHFA 18:1_20:3	5.1450	1.2562	PI 18:0_20:3	4.8556	-1.4628
FAHFA 18:0_20:2	5.8967	1.2531	TG 18:1_22:5_22:6	2.8085	-1.3671
FAHFA 16:0_18:2	5.3682	1.0251	PI 18:1_18:1	4.1106	-1.2726
ACar 20:1	3.6522	0.9835	TG 12:0_12:0_16:0	4.2501	-1.2238
PE 18:1_18:1	2.5298	0.9122	SM d42:0	4.0603	-1.2235
FA 20:3	4.9264	0.8541	PI 18:0_22:6	5.1949	-1.2178
FA 16:1	4.2618	0.7636	CE 22:6	2.8795	-1.2039
ACar 20:0	2.2353	0.7020	TG 58:7	3.6530	-1.1968
PC 33:0	3.0266	0.6918	TG 18:1_18:2_22:5	2.9064	-1.1530
PC 18:1_18:1	4.2607	0.6360	TG 56:7	4.0662	-1.0950
			TG 42:1	4.0251	-1.0826
			TG 58:9	3.6975	-1.0622
			TG 60:12	2.0039	-1.0599
			PI 18:0_20:4	5.7088	-1.0463
			DG 16:0_20:5	2.3635	-1.0335
			PI 18:0_22:5	4.7376	-1.0192

B

Cd+OA+MLT Vs Cd+OA

Upregulated lipids			Downregulated lipids		
Compound	-Log10 adj pvalue	Log2 FC	Compound	-Log10 adj pvalue	Log2 FC
BMP 22:6_22:6	3,5077	3,9842	CE 22:6	3,0204	-1,6910
TG 58:12	3,6466	3,8905	PI 18:1_18:1	6,0597	-1,6374
LPC 0:0/18:2	3,8389	3,1734	PC 42:10	3,4246	-1,2181
TG 62:13	5,9206	2,9031	TG 58:6	2,1921	-1,0773
TG 60:12	3,1393	2,7416	TG 14:0_14:0_15:0	3,3160	-1,0461
TG 50:6	6,0727	2,6791	FA 20:0	6,0597	-0,8421
PC 36:7	3,8945	2,5403	PC 42:9	4,1437	-0,8229
TG 58:11	5,2237	2,4360	PC 42:6	2,1253	-0,7747
TG 52:7	5,9818	2,2925	ACar 16:0	3,6415	-0,7044
TG 18:1_22:5_22:6	6,0727	2,2071	TG 14:0_15:0_18:1	2,0751	-0,6002
DG 16:0_20:5	3,4791	2,1926	FA 22:0	5,3441	-0,5948
TG 50:5	5,1799	2,1873			
SM d30:0	5,2962	2,1175			
DG 18:1_22:6	5,2838	2,0672			
ACar 20:0	4,1319	1,9808			
Cer_NS d18:2_14:0	5,6555	1,9529			
TG 48:4	2,8715	1,9478			
TG 54:8	6,2293	1,9447			
PC 34:5	4,2566	1,9144			
PE 16:0_20:5	4,5404	1,8878			

C

Cd+OAPA+MLT Vs Cd+OAPA					
Upregulated lipids			Downregulated lipids		
Compound	-Log10 adj pvalue	Log2 FC	Compound	-Log10 adj pvalue	Log2 FC

SM d44:2	2,3347	1,6385	FAHFA 16:0_18:2	5,5855	-2,1853
ACar 20:1	3,6525	0,9835	TG 58:12	2,6861	-1,8066
ACar 20:0	2,2135	0,7020	TG 62:13	5,0169	-1,7935
PC 18:1_18:1	4,2601	0,6360	TG 58:11	4,2601	-1,4817
			FAHFA 18:1_20:3	5,1562	-1,3989
			TG 18:1_22:5_22:6	2,8085	-1,3671
			TG 12:0_12:0_16:0	4,2601	-1,2075
			CE 22:6	2,8796	-1,2039
			TG 58:7	3,6531	-1,1968
			TG 18:1_18:2_22:5	2,9065	-1,1530
			FAHFA 2:0_16:2	5,1562	-1,1493
			PI 17:0_20:4	2,8933	-1,1192
			TG 56:7	4,0662	-1,0950
			TG 42:1	4,0251	-1,0826
			TG 58:9	3,6975	-1,0622
			TG 60:12	2,0040	-1,0599
			PI 16:0_20:4	4,2935	-1,0548
			FAHFA 2:0_20:4	4,6785	-1,0386
			DG 16:0_20:5	2,3635	-1,0335
			DG 18:1_22:6	2,9281	-0,9855

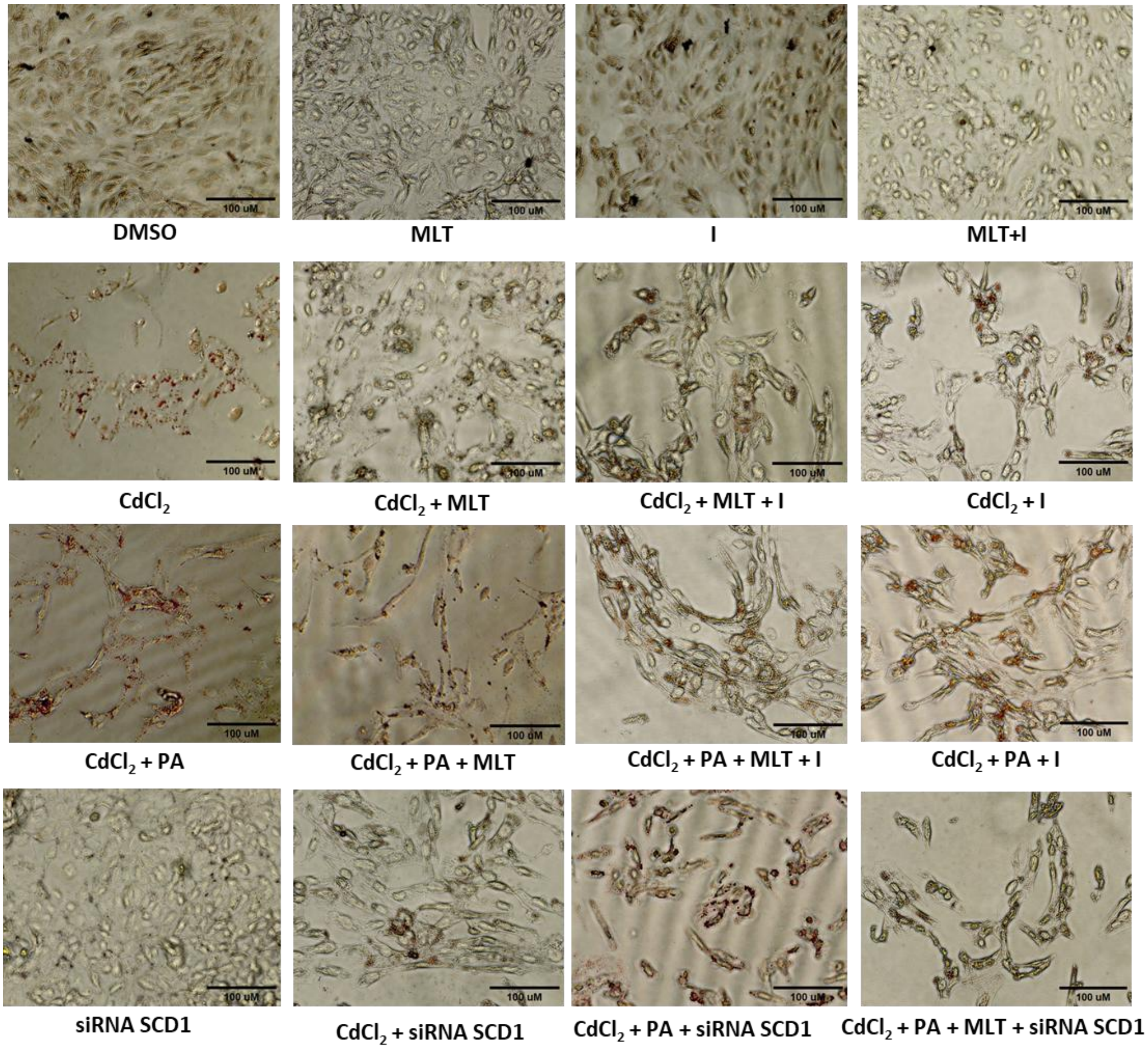

Suppl. Figure S4. Oil Red O Staining of HepaRG Cells Treated with CD and PA in the Presence of SCD1 Inhibitor or siRNA. Microscopy images of Light microscopy images of Oil Red O (ORO) and hematoxylin stained cells to identify lipid droplets and cell nucleus, respectively (40X magnification).
